# Supplementary material for: An in-silico analysis of experimental designs to study ventricular function: A focus on the right ventricle
Source: PLoS Comput Biol. 2022 Sep 20;18(9):e1010017. doi: 10.1371/journal.pcbi.1010017 (PMC9524687; doi:10.1371/journal.pcbi.1010017)
Supplement: S1 Text — (DOCX) [file pcbi.1010017.s001.docx]

Supplementary Material For: *An in-silico analysis of experimental designs to study ventricular function: a focus on the right ventricle*

Mitchel J. Colebank^1,*^, Naomi C. Chesler^1^

^1^University of California, Irvine – Edwards Lifesciences Foundation Cardiovascular Innovation and Research Center, and Department of Biomedical Engineering, University of California, Irvine, Irvine, California, United States of America

*[mjcolebank@gmail.com](mailto:mjcolebank@gmail.com)

**Sarcomere model**

We consider the sarcomere model based on prior work (1,2). The sarcomere is modeled as two series passive elements (describing extracellular matrix (ECM) and Titin contributions) in parallel with a series combination of an active, contractile element and a series, elastic element. Both the ventricles and atria are modeled using identical formulations and deviate only in their parameter values.

The sarcomere length is calculated as a function of the myocardial strain

| $L_{s}=L_{s,ref}\exp\left( \varepsilon_{f} \right)$ | (S1) |
| --- | --- |

where $L_{s,ref}=2.0\mu$m is the reference sarcomere length at zero strain (i.e., $\varepsilon_{f}=0$). The exponential function mimics the nonlinear behavior of sarcomere length under load. Assuming that the change in contractile element length, $L_{sc}$, depends linearly on the change in sarcomere length, the dynamics of $L_{sc}$ ($\mu$m) are

| $\frac{dL_{sc}}{dt}=\left( \frac{L_{s}-L_{sc}}{L_{s,iso}}-1 \right)v_{0}$ | (S2) |
| --- | --- |

where $L_{s,iso}$ ($\mu$m) is the length of the elastic series element in an isometrically stressed state, and $v_{0}$ ($\mu$m/s) is the velocity of sarcomere shortening with zero load (1).

The mechanical activation of the sarcomere is heuristically modeled as separate “rise” and “decay” functions. The former is given by

| $\Gamma_{rise}=\frac{1}{\tau_{rise}}C_{L}\left( L_{sc} \right)F_{rise}.$ | (S3) |
| --- | --- |

$C_{L}$ and $F_{rise}$ represent the increase in contractility with sarcomere length and with time, respectively. They are given by

| $C_{L}\left( L_{sc} \right)=\tanh\left( 4\left( L_{sc}-L_{sc,0} \right)^{2} \right),$ $F_{rise}\left( t \right)=0.02 x^{3}\left( 8-x^{2} \right)e^{-x} , x=\left( 8,\left( \frac{t}{\tau_{rise}} \right) \right)$ | (S4) |
| --- | --- |

where $\tau_{rise}$ (s) scales the rise in contractility and $L_{sc,0}$ ($\mu$m) represents the contractile element length with zero active stress. The decay function is

| $\Gamma_{decay}=\frac{1}{\tau_{decay}}\left( \frac{\Gamma_{rest}-\Gamma\left( t \right)}{1+\exp\left( T\left( L_{sc} \right)-t)/\tau_{decay} \right)} \right)$ | (S5) |
| --- | --- |

where $\Gamma_{rest}$ is the diastolic resting level of activation, $\tau_{decay}$ (s) is the decay time and

| $T\left( L_{sc} \right)=\tau_{sys}\left( 0.29+0.3L_{sc} \right)$ | (S6) |
| --- | --- |

represents the decay in activation with decreasing sarcomere length, which depends on the duration of systole, $\tau_{sys}$ (s). Both rise and decay functions are combined to represent the change in contractility

| $\frac{d\Gamma}{dt}=\Gamma_{rise}+\Gamma_{decay}.$ | (S7) |
| --- | --- |

The active stress, $G_{act}$ is finally calculated as the product of contractility, contractile element displacement, and the strain of the series elastic element

| $G_{act}=\sigma_{act} \Gamma\left( L_{sc}-L_{sc,0} \right)\left( \frac{L_{s}-L_{sc}}{L_{se,iso}} \right)$ | (S8) |
| --- | --- |

where $\sigma_{act}$ (KPa) is a parameter that scales the active stress contribution.

For the passive stress, we consider a new, *passive* reference length $L_{s,pas,ref}$ ($\mu$m), giving the passive strain

| $\lambda_{s,pas}=\exp\left( \varepsilon_{f} \right)\frac{L_{s,ref}}{L_{s,pas,ref}}=\frac{L_{sc}}{L_{s,pas,ref}} .$ | (S9) |
| --- | --- |

The two passive stresses are as defined in Walmsley et al. (3)

| $G_{ECM}=0.0349 \sigma_{pas} \left( {\lambda_{s,pas}}^{k_{1}}-1 \right)$ | (S10) |
| --- | --- |
| $G_{Titin}=0.01 \sigma_{act} \left( {\lambda_{s,pas}}^{2L_{s,ref}/\beta_{pas}}-1 \right)$ | (S11) |

where  $\sigma_{pas}$ (KPa) is the factor scaling the passive stress of the ECM, $k_{1}$ (dimensionless) is an exponential scaling parameter, and $\beta_{pas}$ ($\mu$m^-1^) is a measure of Titin stiffness (3).

**Cardiac chamber equations**

For the atria, the mid-wall volume, $V_{m}$, mid-wall curvature, $C_{m}$, and mid-wall cross-sectional area, $A_{m}$, are determined by

| $V_{m}=V\left( t \right)+\frac{1}{2}V_{wall},$ | (S12) |
| --- | --- |
| $C_{m}=\left( \frac{4\pi}{3V_{m}} \right)^{1/3},$ | (S13) |
| $A_{m}=\frac{4\pi}{C_{m}^{2}}.$ | (S14) |

Since the LV, RV, and S are mechanically coupled, a separate formulation for $V_{m}$, $C_{m}$, and $A_{m}$ is required. Utilizing the common radius of mid-wall junction point $y_{m}$ and denoting the maximal axial distance from each chamber wall surface to the origin as $x_{m}$ (1,4), we get

| $V_{m}=\frac{\pi}{6}x_{m}\left( x_{m}^{2}+3y_{m}^{2} \right)$ | (S15) |
| --- | --- |
| $C_{m}=\frac{2x_{m}}{\left( x_{m}^{2}+y_{m}^{2} \right)},$ | (S16) |
| $A_{m}=\pi\left( x_{m}^{2}+y_{m}^{2} \right),$ | (S17) |

for the LV, RV, and S. As in the original work by Lumens (1), we can also related $V_{m}$ to the blood volume $V\left( t \right)$ in the chamber. Specifically, $V_{m,LV}$ and $V_{m,RV}$ are

| $V_{m,LV}=-V_{LV}\left( t \right)-\frac{1}{2}V_{wall,LV}-\frac{1}{2}V_{wall,S}+V_{m,S}$ | (S18) |
| --- | --- |
| $V_{m,RV}=-V_{RV}\left( t \right)+\frac{1}{2}V_{wall,LV}+\frac{1}{2}V_{wall,S}+V_{m,S},$ | (S19) |

which is updated at each time point. Hence, we solve for $x_{m}$ using in equation (S15) to deduce $A_{m}$ and $C_{m}$ for each TriSeg component. The atrial transmural pressure is determined from the wall tension and mid-wall curvature

| $p=2T_{m}C_{m}.$ | (S20) |
| --- | --- |

The ventricular mid-wall tension is broken up into the axial ($T_{x}$) and radial ($T_{y}$) tensions based on the geometry of the spherical chambers and the angle of the sphere opening (1), giving

| $T_{x}=T_{m}\sin\left( \frac{2x_{m}y_{m}}{x_{m}^{2}+y_{m}^{2}} \right), T_{y}=T_{m}\cos\left( \frac{-x_{m}^{2}+y_{m}^{2}}{x_{m}^{2}+y_{m}^{2}} \right).$ | (S21) |
| --- | --- |

These tensions must be balanced across the LV, RV, and septal wall, and serve as the algebraic constraints for the system. The axial tensions in the ventricular cavities are then used to calculate the transmural pressure

| $p=\frac{2T_{x}}{y_{m}}.$ | (S22) |
| --- | --- |

**Nominal Parameter Values**

The parameters utilized in the manuscript are based off of several works, with a majority of the calibration coming from data in sham mice obtained in Philip et al. (5). Total blood volume in the mouse is determined by

| $V_{tot}=BW\cdot84.7\frac{\mathrm{kg}}{\mathrm{ml}}$ | (S23) |
| --- | --- |

as postulated by Riches et al. (6). Here, the bodyweight is set to 29.719 grams (giving a total blood volume $V_{tot}=2.517$ ml) and the heart rate is 554 beats per minute, giving a cardiac cycle length $T=0.1083$ (5). Systolic and diastolic pressures in the left ventricle (LV) were set at 76 and 2 mmHg, respectively. The corresponding systolic and diastolic values in the right ventricle (RV) were set to 19.6 and 0.74 mmHg (5). The cardiac output (CO) is 10.6 ml/min, and converted to $\mu$l/s. The assumed nominal pressures in the other cardiovascular compartment are calculated as a function of LV and RV pressures, as detailed in Table S1. The unstressed volumes (i.e., the blood not ejected during a cardiac cycle) and stressed volumes in each compartment are based on previous work (7–9) and provided in Table S2. The compartment pressures and stressed volumes are used to construct nominal estimates of vascular resistance and compliance, shown in Table S3. Tables S4 and S5 show the calculated values of the Triseg geometry and the sarcomere model, respectively. Consistent units were necessary for multiscale interactions between model components, hence pressures were converted to KPa using the relation 1 mmHg = 0.133322 KPa, areas were converted from mm^2^ to cm^2^, and volumes were converted from $\mu$l to ml. Outputs from the model are subsequently scaled back to their original units for clarity in the results.

**Results from the Morris screening**

The results the Morris screening are provided in Fig S1. Table S6 shows the upper and lower bounds for each parameter expressed as the percent change from their nominal value.

**Fig S1:** Comparison plot of the modified average elementary effect, $\mu^{*}$, against elementary effect variance, $s^{2},$ for each of the output quantities of interest. Results from the Morris screening show a trend of higher mean effects and higher variance in elementary effects, which indicate an influential parameter.

**Results from Bayesian Inference**

To assess the accuracy of the Markov chain Monte Carlo (MCMC) techniques used in the main text, we plot the relative difference between the true, data generating parameter, $\boldsymbol{\theta}^{\boldsymbol{*}}$, and the median values from the posterior distributions, $\boldsymbol{\theta}_{med}^{MCMC}$, i.e.

| $Relative Difference=\frac{\boldsymbol{\theta}_{med}^{MCMC}-\boldsymbol{\theta}^{\boldsymbol{*}}}{\boldsymbol{\theta}^{\boldsymbol{*}}}.$ | (S24) |
| --- | --- |

Boxplots depicting this quantity across all four experimental designs are shown in Figure S2. As noted in the text, wall volume parameters ($V_{wall}$) tended to be the most volatile during inference, as well as resistance and compliance elements. This is especially true for designs $\boldsymbol{f}_{1}$ and $\boldsymbol{f}_{2}$, where no LV data is available. However, designs with LV data, $\boldsymbol{f}_{3}$ and $\boldsymbol{f}_{4}$, reduce this variability.

In addition to the methods of the main texts, pairwise plots were analyzed for any strong correlations. This may be indicative of practical identifiability issues. All pairwise plots are provided in Figures S3 through S50 in S2 text. Of these, only one exhibits some issues with practical identifiability (see Figure S35).

**Fig S2:** Relative error in the median posterior values compared to the true, data generating parameters. Black squares represent the true parameter values while black circles indicate the mean value across the median posterior value for the 12 instances of MCMC.

**Table S1.** Formulas for nominal pressure values (presented in mmHg for convenience).

| **Variable** | **Method** | **Reference** |
| --- | --- | --- |
| $p_{LV,sys}$ | 76.00 | Data from (5) |
| $p_{LV,dias}$ | 2.00 | Data from (5) |
| $p_{RV,sys}$ | 19.60 | Data from (5) |
| $p_{RV,dias}$ | 0.74 | Data from (5) |
| $p_{LA,sys}$ | $1.5\cdot p_{LV,dias}$ | Scaled from normotensive humans (7) |
| $p_{LA,dias}$ | $0.9\cdot p_{sv,mean}$ | Scaled from normotensive humans (7) |
| $p_{RA,sys}$ | $1.25\cdot p_{RV,dias}$ | Scaled from normotensive humans (7) |
| $p_{RA,dias}$ | $0.9\cdot p_{pv,mean}$ | Scaled from normotensive humans (7) |
| $p_{sa,sys}$ | $0.99\cdot p_{LV,sys}$ | Scaled from normotensive humans (7) |
| $p_{sa,dias}$ | $0.66\cdot p_{LV,sys}$ | Scaled from normotensive humans (7) |
| $p_{sa,mean}$ | $\left( p_{sa,sys}+2p_{sa,dias} \right)/3$ | Clinical definition (7) |
| $p_{pa,sys}$ | $0.99\cdot p_{RV,sys}$ | Scaled from normotensive humans (7) |
| $p_{pa,dias}$ | $0.27\cdot p_{RV,sys}$ | Scaled from normotensive humans (7) |
| $p_{pa,mean}$ | $\left( p_{pa,sys}+2p_{pa,dias} \right)/3$ | Clinical definition (7) |
| $p_{sys,cap}$ | $0.26\cdot p_{sa,mean}$ | Scaled from normotensive humans (7) |
| $p_{pulm,cap}$ | $0.66\cdot p_{pa,mean}$ | Scaled from normotensive humans (7) |
| $p_{sv,mean}$ | $0.12\cdot p_{sa,mean}$ | Scaled from normotensive humans (7) |
| $p_{pv,mean}$ | $0.33\cdot p_{pa,mean}$ | Scaled from normotensive humans (7) |

**Table S2.** Formulas for nominal unstressed ($V_{i,un}$) and stressed volume ($V_{i}$) values (converted from $\mu l$ to ml$\cdot{10}^{-3}$).

| **Variable** | **Method** | **Reference** |
| --- | --- | --- |
| $V_{sa,un}$ | $0.14\cdot V_{tot}$ | Based on (7) |
| $V_{sv,un}$ | $0.70\cdot V_{tot}$ | Based on (7) |
| $V_{RA,un}$ | $0.01\cdot V_{tot}$ | Based on (7) |
| $V_{RV,un}$ | $0.026\cdot V_{tot}$ | Based on (7) |
| $V_{pa,un}$ | $0.026\cdot V_{tot}$ | Based on (7) |
| $V_{pv,un}$ | $0.062\cdot V_{tot}$ | Based on (7) |
| $V_{LA,un}$ | $0.010\cdot V_{tot}$ | Based on (7) |
| $V_{LV,un}$ | $0.026\cdot V_{tot}$ | Based on (7) |
| $V_{sa}$ | $0.27\cdot V_{sa,un}$ | Based on (8) |
| $V_{sv}$ | $0.075\cdot V_{sv,un}$ | Based on (8) |
| $V_{RA}$ | $1.0\cdot V_{RA,un}$ | Based on (8) |
| $V_{RV}$ | $1.0\cdot V_{RV,un}$ | Based on (8) |
| $V_{pa}$ | $0.58\cdot V_{pa,un}$ | Based on (8) |
| $V_{pv}$ | $0.11\cdot V_{pv,un}$ | Based on (8) |
| $V_{LA}$ | $1.0\cdot V_{LA,un}$ | Based on (8) |
| $V_{LV}$ | $1.0\cdot V_{LV,un}$ | Based on (8) |
| $V_{m,s}$ | $0.0084\cdot V_{tot}$ | Hand tuned |

**Table S3.** Formulas for nominal hemodynamic resistances ($R$, KPa$\cdot$s/ml) and compliances ($C$, ml/KPa).

| **Parameter** | **Method/Value** | **Description** | **Reference** |
| --- | --- | --- | --- |
| $R_{m,val}$ | $\frac{p_{LA,sys} -p_{LV,dias}}{CO}$ | Mitral valve resistance | Poiseuille/Ohm’s Law |
| $R_{a,val}$ | $\frac{p_{LV,sys} -p_{sa,sys}}{CO}$ | Aortic valve resistance | Poiseuille/Ohm’s Law |
| $R_{t,val}$ | $\frac{p_{RA,sys} -p_{RV,dias}}{CO}$ | Tricuspid valve resistance | Poiseuille/Ohm’s Law |
| $R_{p,val}$ | $\frac{p_{RV,sys} -p_{pa,sys}}{CO}$ | Pulmonic valve resistance | Poiseuille/Ohm’s Law |
| $R_{vc}$ | $\frac{p_{sv,mean} -p_{ra,dias}}{CO}$ | Vena Cava resistance | Poiseuille/Ohm’s Law |
| $R_{pv}$ | $\frac{p_{pv,mean} -p_{la,dias}}{CO}$ | Pulmonary venous resistance | Poiseuille/Ohm’s Law |
| $R_{sys}$ | $\frac{p_{sa,mean} -p_{sys,cap}}{CO}$ | Systemic vascular resistance | Poiseuille/Ohm’s Law |
| $R_{pulm}$ | $\frac{p_{pa,mean} -p_{pulm,cap}}{CO}$ | Pulmonary vascular resistance | Poiseuille/Ohm’s Law |
| $C_{sa}$ | $\frac{V_{sa}}{p_{sa,sys}}$ | Systemic arterial compliance | Based on (10,11) |
| $C_{sv}$ | $\frac{V_{sv}}{p_{sv,mean}}$ | Systemic venous compliance | Based on (10,11) |
| $C_{pa}$ | $\frac{V_{pa}}{p_{pa,sys}}$ | Pulmonary arterial compliance | Based on (10,11) |
| $C_{pv}$ | $\frac{V_{pv}}{p_{pv,mean}}$ | Pulmonary venous compliance | Based on (10,11) |

**Table S4.** Formulas for nominal TriSeg geometry parameter values (presented in $\mu l$ and mm^2^ for convenience).

| **Parameter** | **Method/Value** | **Description** | **Reference** |
| --- | --- | --- | --- |
| $V_{wall,LA}$ | 11.00 $\mathrm{mm}^{3}$ | Wall volume of left atrium | Hand tuned from (12) |
| $V_{wall,LV}$ | 78.10 $\mathrm{mm}^{3}$ | Wall volume of left ventricle | Hand tuned from (12) |
| $V_{wall,RA}$ | 11.00 $\mathrm{mm}^{3}$ | Wall volume of right atrium | Hand tuned from (12) |
| $V_{wall,RV}$ | 31.24 $\mathrm{mm}^{3}$ | Wall volume of right ventricle | Hand tuned from (12) |
| $V_{wall,S}$ | 42.13 $\mathrm{mm}^{3}$ | Wall volume of septum | Hand tuned from (12) |
| $A_{m,ref,LA}$ | 35 mm^2^ | Reference mid-wall area of left atrium | Hand tuned |
| $A_{m,ref,LV}$ | 59 mm^2^ | Reference mid-wall area of left ventricle | From (12) |
| $A_{m,ref,RA}$ | 35 mm^2^ | Reference mid-wall area of right atrium | Hand tuned |
| $A_{m,ref,RV}$ | 50 mm^2^ | Reference mid-wall area of right ventricle | From (12) |
| $A_{m,ref,S}$ | 10 mm^2^ | Reference mid-wall area of septum | From (12) |

**Table S5.** Formulas and values for nominal sarcomere model parameters. All parameters and values are shown as atrium/ventricle.

| **Parameter** | **Value** | **Description** | **Reference** |
| --- | --- | --- | --- |
| $L_{s,ref,A}/ L_{s,ref,V}$ | 2.0/2.0 $\mu$m | Reference sarcomere length at zero strain | From (1) |
| $L_{s,iso,A}/L_{s,iso,V}$ | 0.04/0.04 $\mu$m | Length of elastic series element in isometric stress | From (1) |
| $v_{0,A}/ v_{0,V}$ | 30/12 $\mu$m/s | Velocity of sarcomere shortening | Hand tuned from (12) |
| $L_{sc,0,A}/ L_{sc,0,V}$ | 1.51/1.51 $\mu$m | Reference length of contractile element | From (1) |
| $\Gamma_{rest,A}/\Gamma_{rest,V}$ | $0.02/0.02$ | Resting contractility | From (1) |
| $\tau_{rise,A}/\tau_{rise,V}$ | $4.06/2.17\cdot{10}^{-3}$ s | Scaling parameter for contractility rise time | Hand tuned from (1,5) |
| $\tau_{decay,A}/\tau_{decay,V}$ | $0.54/8.90\cdot{10}^{-3}$ s | Scaling parameter for contractility decay time | Hand tuned from (1,5) |
| $\tau_{sys,A}/\tau_{sys,V}$ | $1.62/4.87\cdot{10}^{-2}$ s | Parameter describing systolic length | Hand tuned from (1,5) |
| $\tau_{offset,A}$ | $1.95\cdot{10}^{-2}$ s | Time delay in atrial and ventricular contraction | Hand tuned |
| $\bar{\sigma}_{act,A}/\bar{\sigma}_{act,V}$ | 38.28/25.52 KPa | Scaling parameter for active stress | Hand tuned from (13) |
| $\bar{\sigma}_{pas,A}/\bar{\sigma}_{pas,V}$ | 2.24/1.49 KPa | Scaling parameter for passive stress | Hand tuned from (13) |
| $L_{s,ref,pas,A}/L_{s,ref,pas,V}$ | 1.8/1.8 $\mu$m | Reference length of passive wall constituents | From (1) |
| $\beta_{pas,A}/\beta_{pas,V}$ | 0.6/0.6 $\mu$m | Measure of Titin stiffness | From (1) |
| $k_{1,A}/k_{1,V}$ | 10/10 | Parameter describing nonlinear ECM strain | From (1) |

**Table S6.** Parameter bounds for Morris screening.

| **Parameter** | **Bounds as % around nominal** | **Parameter** | **Bounds as % around nominal** |
| --- | --- | --- | --- |
| $V_{la,wall}$ | $\left[ 50,150 \right]$ | $\tau_{rise,V}$ | $\left[ 75,125 \right]$ |
| $V_{lv,wall}$ | $\left[ 50,150 \right]$ | $\tau_{decay,V}$ | $\left[ 75,125 \right]$ |
| $V_{ra,wall}$ | $\left[ 50,150 \right]$ | $\tau_{sys,V}$ | $\left[ 75,125 \right]$ |
| $V_{rv,wall}$ | $\left[ 50,150 \right]$ | $\bar{\sigma}_{act,V}$ | $\left[ 75,125 \right]$ |
| $V_{s,wall}$ | $\left[ 50,150 \right]$ | $\bar{\sigma}_{pas,V}$ | $\left[ 75,125 \right]$ |
| $A_{m,ref,la}$ | $\left[ 75,125 \right]$ | $\beta_{pas,V}$ | $\left[ 75,125 \right]$ |
| $A_{m,ref,lv}$ | $\left[ 75,125 \right]$ | $k_{1,V}$ | $\left[ 75,125 \right]$ |
| $A_{m,ref,ra}$ | $\left[ 75,125 \right]$ | $R_{a,val}$ | $\left[ 50,300 \right]$ |
| $A_{m,ref,rv}$ | $\left[ 75,125 \right]$ | $R_{m,val}$ | $\left[ 50,300 \right]$ |
| $A_{m,ref,s}$ | $\left[ 75,125 \right]$ | $R_{p,val}$ | $\left[ 50,300 \right]$ |
| $v_{max,A}$ | $\left[ 75,125 \right]$ | $R_{t,val}$ | $\left[ 50,300 \right]$ |
| $\tau_{rise,A}$ | $\left[ 75,125 \right]$ | $R_{vc}$ | $\left[ 50,300 \right]$ |
| $\tau_{decay,A}$ | $\left[ 75,125 \right]$ | $R_{pv}$ | $\left[ 50,300 \right]$ |
| $\tau_{sys,A}$ | $\left[ 75,125 \right]$ | $R_{sys}$ | $\left[ 50,300 \right]$ |
| $\bar{\sigma}_{act,A}$ | $\left[ 75,125 \right]$ | $R_{pulm}$ | $\left[ 50,300 \right]$ |
| $\bar{\sigma}_{pas,A}$ | $\left[ 75,125 \right]$ | $C_{sa}$ | $\left[ 10,300 \right]$ |
| $\beta_{pas,A}$ | $\left[ 75,125 \right]$ | $C_{sv}$ | $\left[ 10,300 \right]$ |
| $k_{1,A}$ | $\left[ 75,125 \right]$ | $C_{pa}$ | $\left[ 10,300 \right]$ |
| $v_{max,V}$ | $\left[ 75,125 \right]$ | $C_{pv}$ | $\left[ 10,300 \right]$ |

References

1. Lumens J, Delhaas T, Kirn B, Arts T. Three-Wall Segment (TriSeg) Model Describing Mechanics and Hemodynamics of Ventricular Interaction. Ann Biomed Eng. 2009;37: 2234–2255. doi:10.1007/s10439-009-9774-2

2. Walmsley J, Arts T, Derval N, Bordachar P, Cochet H, Ploux S, et al. Fast Simulation of Mechanical Heterogeneity in the Electrically Asynchronous Heart Using the MultiPatch Module. McCulloch AD, editor. PLOS Comput Biol. 2015;11: e1004284. doi:10.1371/journal.pcbi.1004284

3. Walmsley J, Huntjens PR, Prinzen FW, Delhaas T, Lumens J. Septal flash and septal rebound stretch have different underlying mechanisms. Am J Physiol Circ Physiol. 2016;310: H394–H403. doi:10.1152/ajpheart.00639.2015

4. Marzban B, Lopez R, Beard DA. Computational Modeling of Coupled Energetics and Mechanics in the Rat Ventricular Myocardium. Physiome. 2020. doi:10.36903/physiome.12964970

5. Philip JL, Murphy TM, Schreier DA, Stevens S, Tabima DM, Albrecht M, et al. Pulmonary vascular mechanical consequences of ischemic heart failure and implications for right ventricular function. Am J Physiol Circ Physiol. 2019;316: H1167–H1177. doi:10.1152/ajpheart.00319.2018

6. Riches AC, Sharp JG, Thomas DB, Smith SV. Blood volume determination in the mouse. J Physiol. 1973;228(2):279–84.

7. Boron WF, Boulpaep EL. Medical physiology. Philadelphia, PA : Elsevier; 2017.

8. Beneken JEW, DeWit B. A physical approach to hemodynamic aspects of the human cardiovascular system. In: Guyton A, Reeve E, editors. Physical Bases of Circulatory Transport: Regulation and Exchange. Philadelphia: Saunders; 1966. p. 1–45.

9. Williams ND, Brady R, Gilmore S, Gremaud P, Tran HT, Ottesen JT, et al. Cardiovascular dynamics during head-up tilt assessed via pulsatile and non-pulsatile models. J Math Biol. 2019;79(3):987–1014. doi:10.1007/s00285-019-01386-9

10. Marquis AD, Arnold A, Dean-Bernhoft C, Carlson BE, Olufsen MS. Practical identifiability and uncertainty quantification of a pulsatile cardiovascular model. Math Biosci. 2018;304: 9–24. doi:10.1016/j.mbs.2018.07.001

11. Colunga AL, Kim KG, Woodall NP, Dardas TF, Gennari JH, Olufsen MS, et al. Deep phenotyping of cardiac function in heart transplant patients using cardiovascular system models. J Physiol. 2020;598: 3203–3222. doi:10.1113/JP279393

12. Tewari SG, Bugenhagen SM, Wang Z, Schreier DA, Carlson BE, Chesler NC, et al. Analysis of cardiovascular dynamics in pulmonary hypertensive C57BL6/J mice. Front Physiol. 2013;4: 1–9. doi:10.3389/fphys.2013.00355

13. Wang Z, Patel JR, Schreier DA, Hacker TA, Moss RL, Chesler NC. Organ-level right ventricular dysfunction with preserved Frank-Starling mechanism in a mouse model of pulmonary arterial hypertension. J Appl Physiol. 2018;124: 1244–1253. doi:10.1152/japplphysiol.00725.2017
