# Supplementary material for: An in-silico analysis of experimental designs to study ventricular function: A focus on the right ventricle
Source: PLoS Comput Biol. 2022 Sep 20;18(9):e1010017. doi: 10.1371/journal.pcbi.1010017 (PMC9524687; doi:10.1371/journal.pcbi.1010017)

Supplementary Material For: *An in-silico analysis of experimental designs to study ventricular function: a focus on the right ventricle*

Mitchel J. Colebank^1,*^, Naomi C. Chesler^1^

^1^University of California, Irvine – Edwards Lifesciences Foundation Cardiovascular Innovation and Research Center, and Department of Biomedical Engineering, University of California, Irvine, Irvine, California, United States of America

*[mjcolebank@gmail.com](mailto:mjcolebank@gmail.com)

Figure S3: Residual 1 – Iteration 1


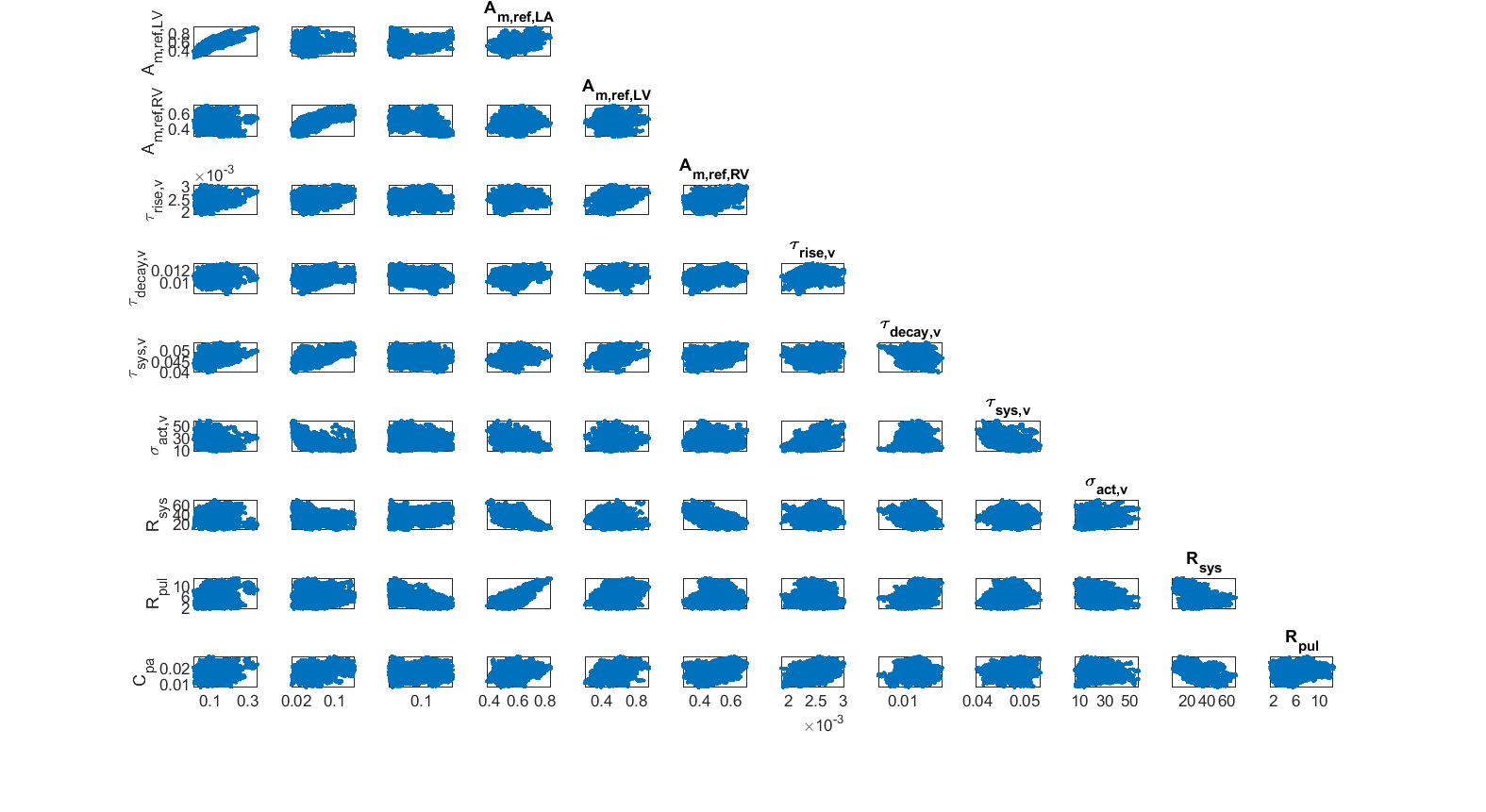

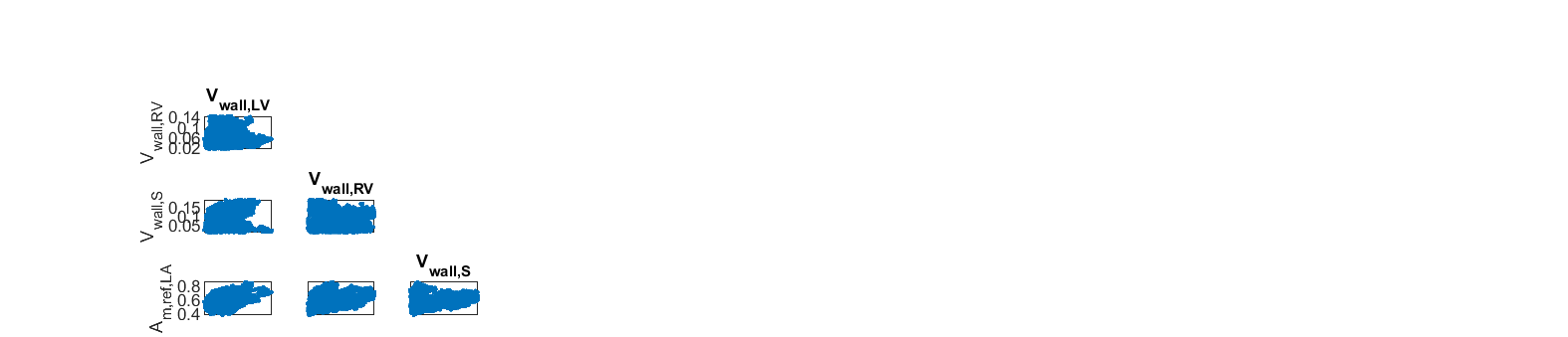


Figure S4: Residual 1 – Iteration 2


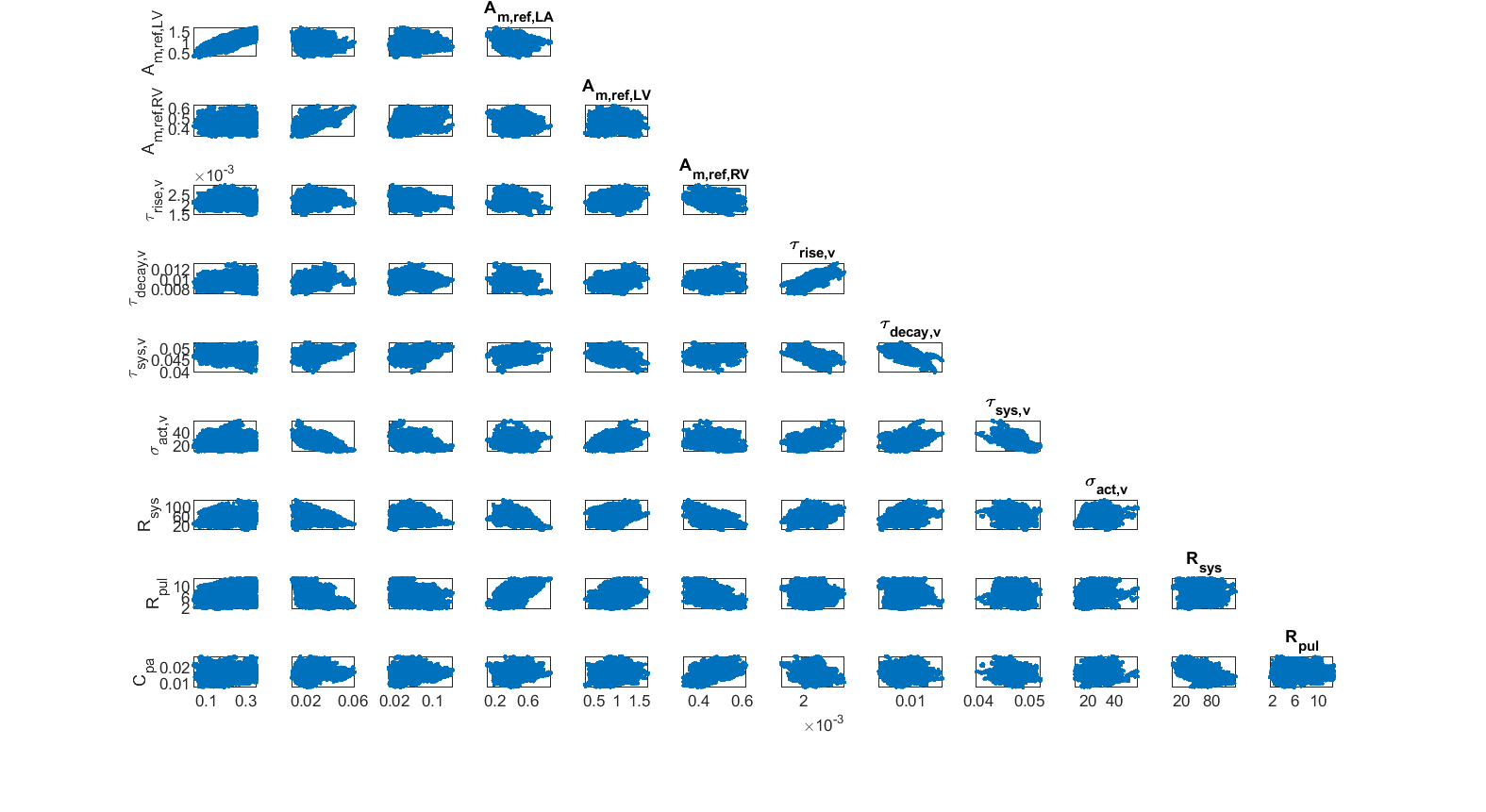

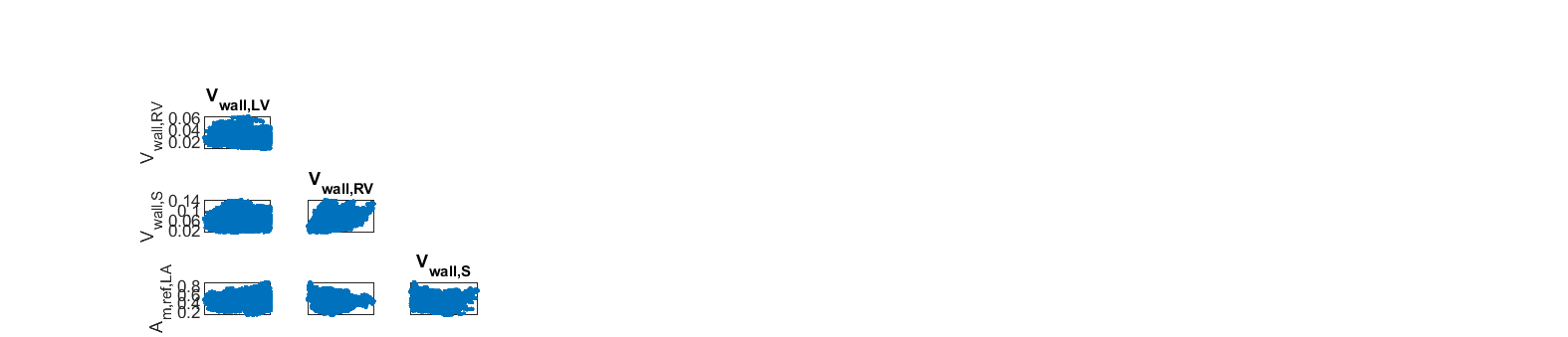


Figure S5 Residual 1 – Iteration 3


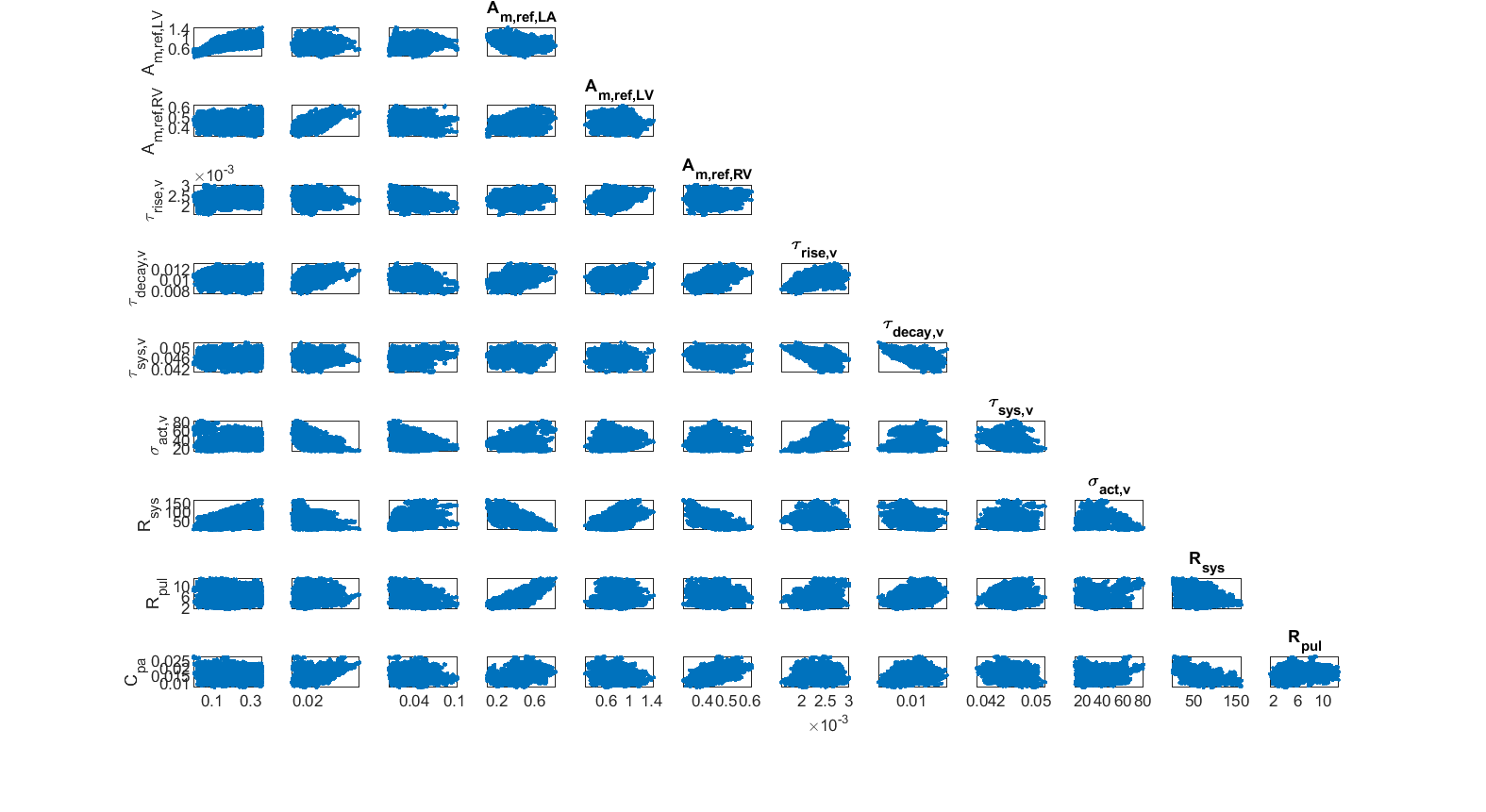

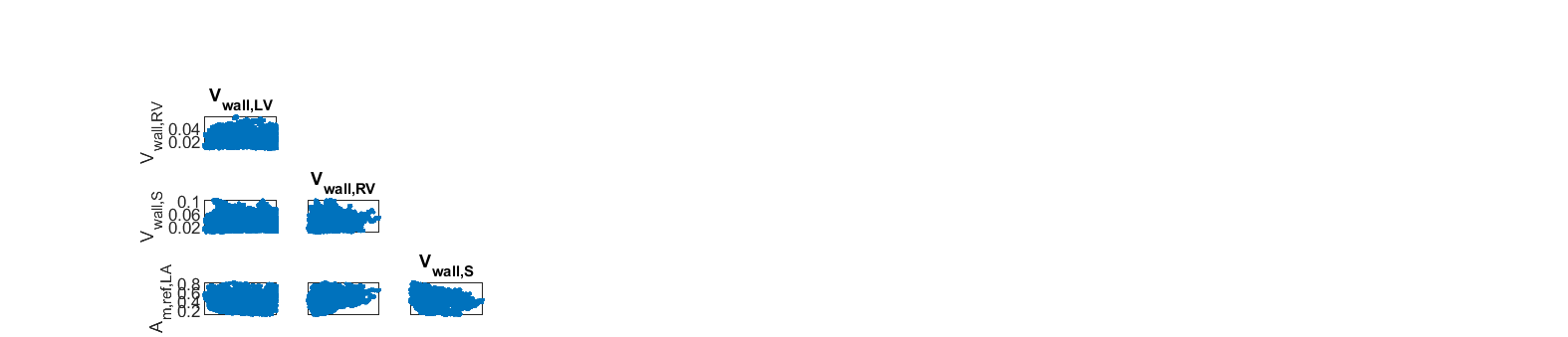


Figure S6 Residual 1 – Iteration 3


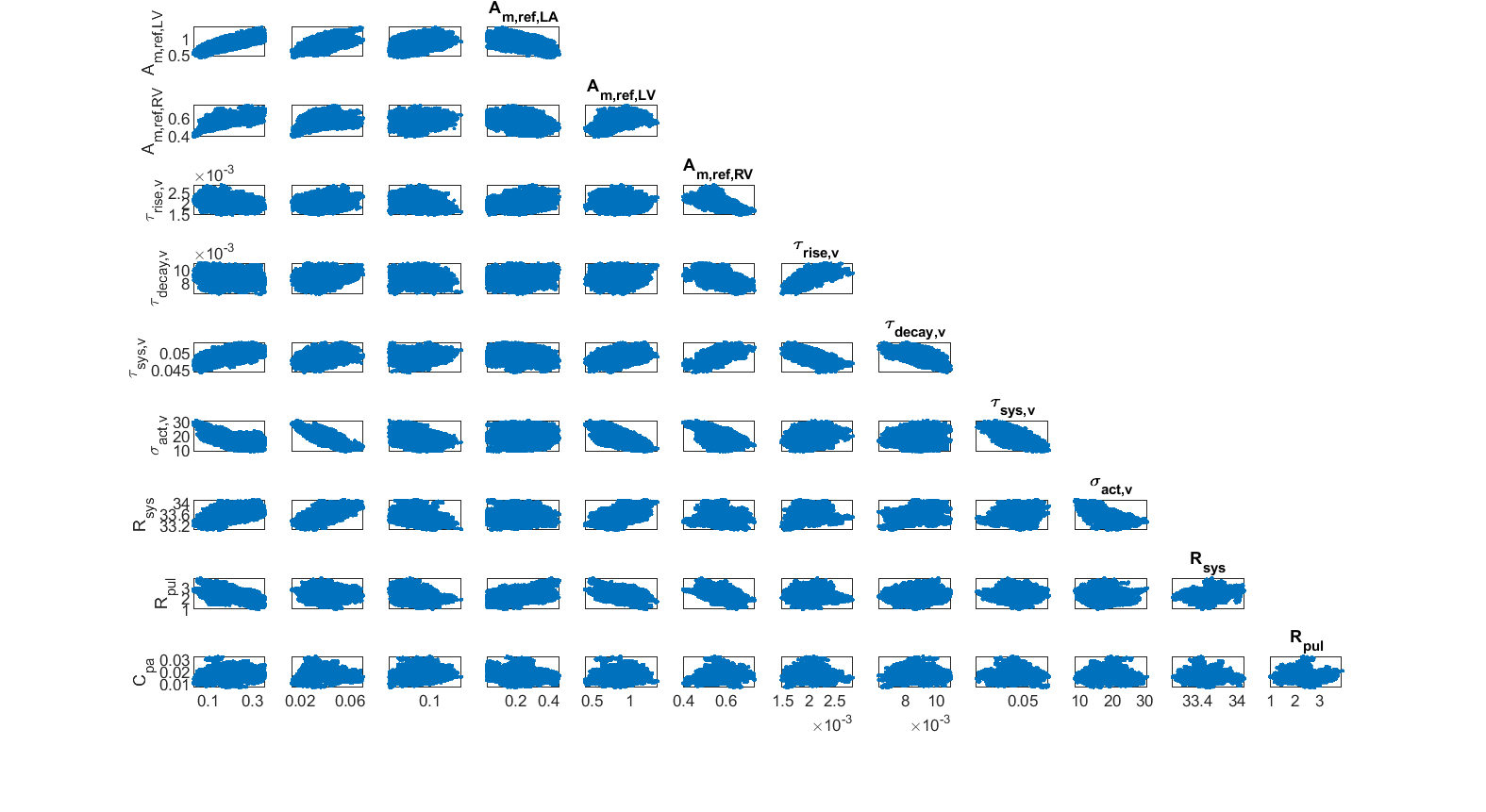

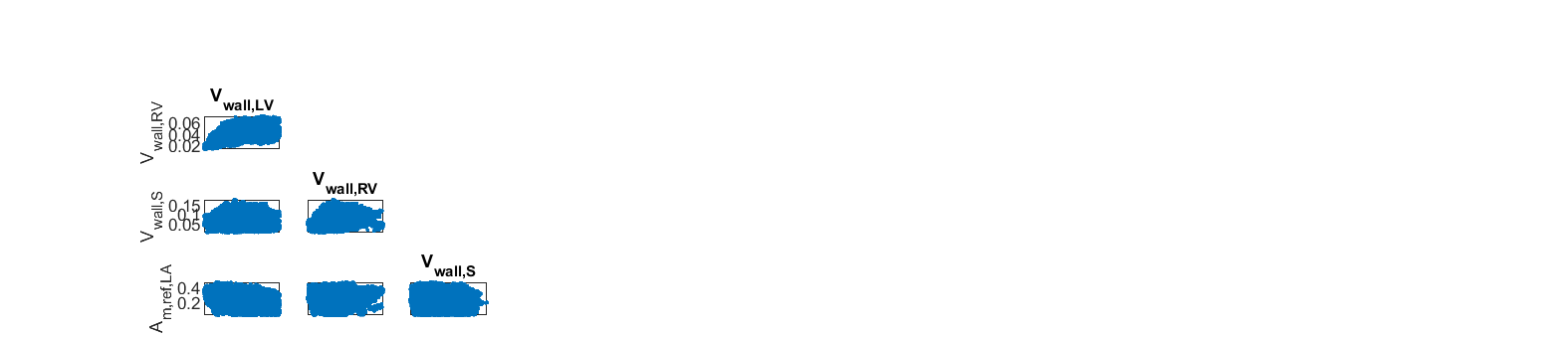

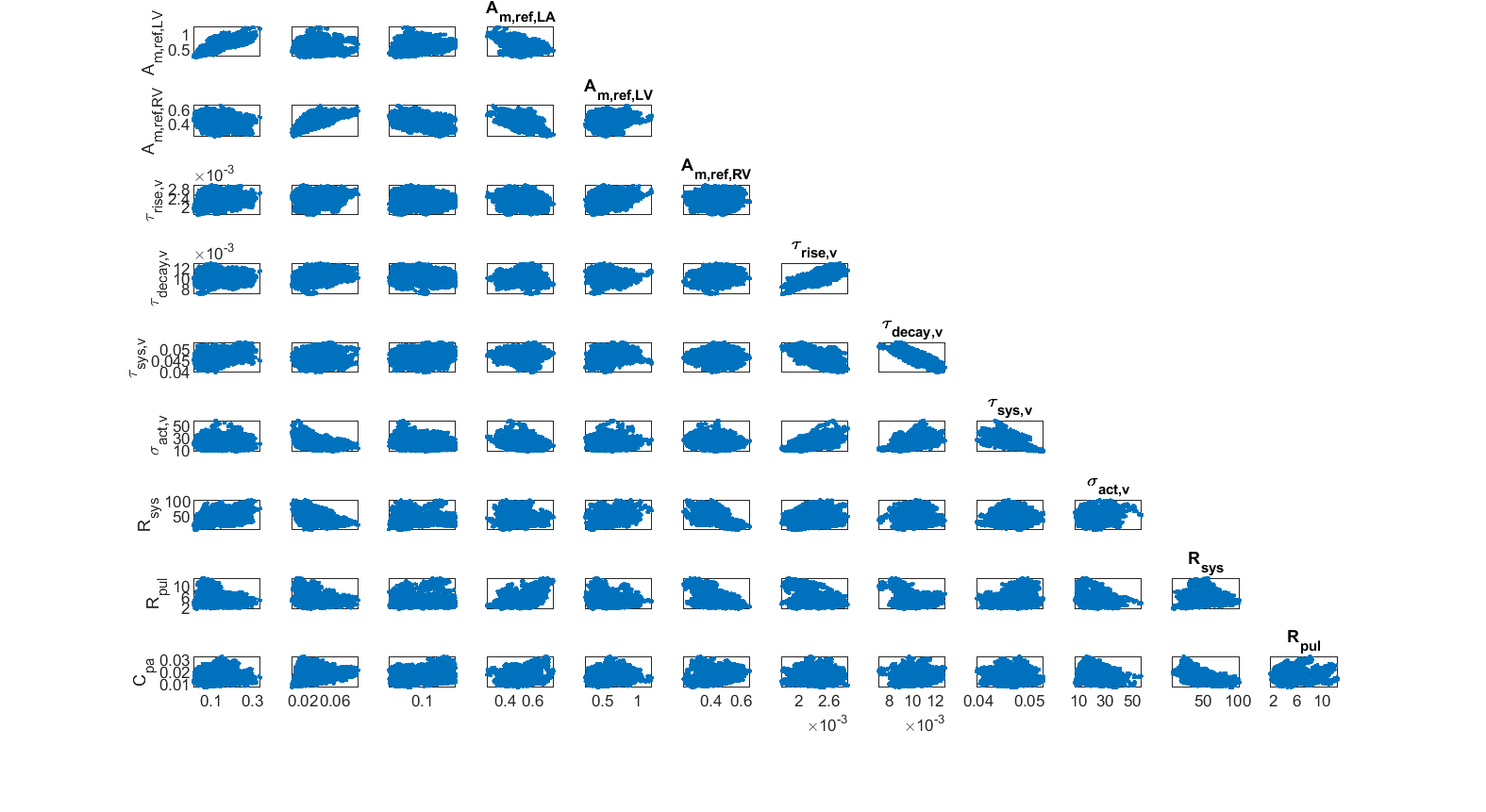

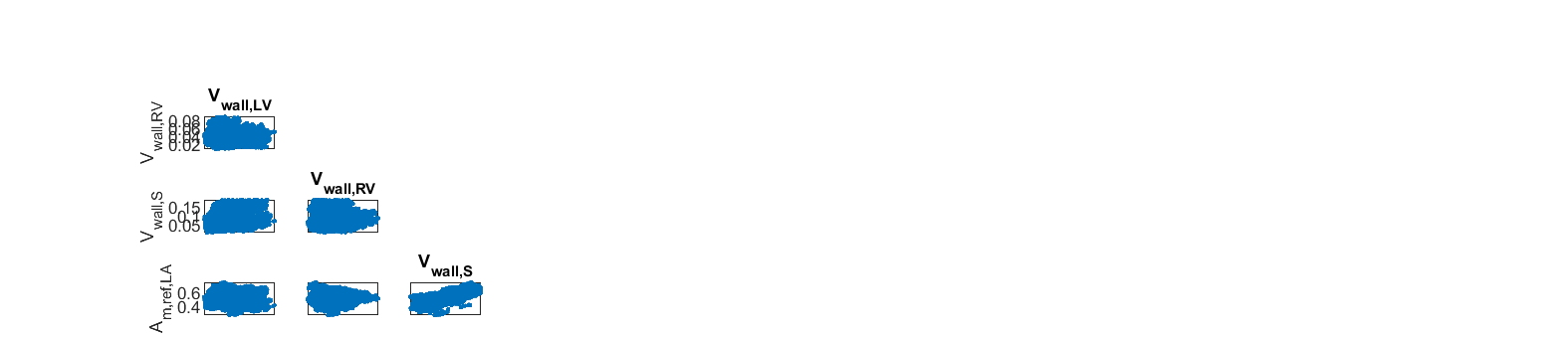


Figure S7: Residual 1 – Iteration 5


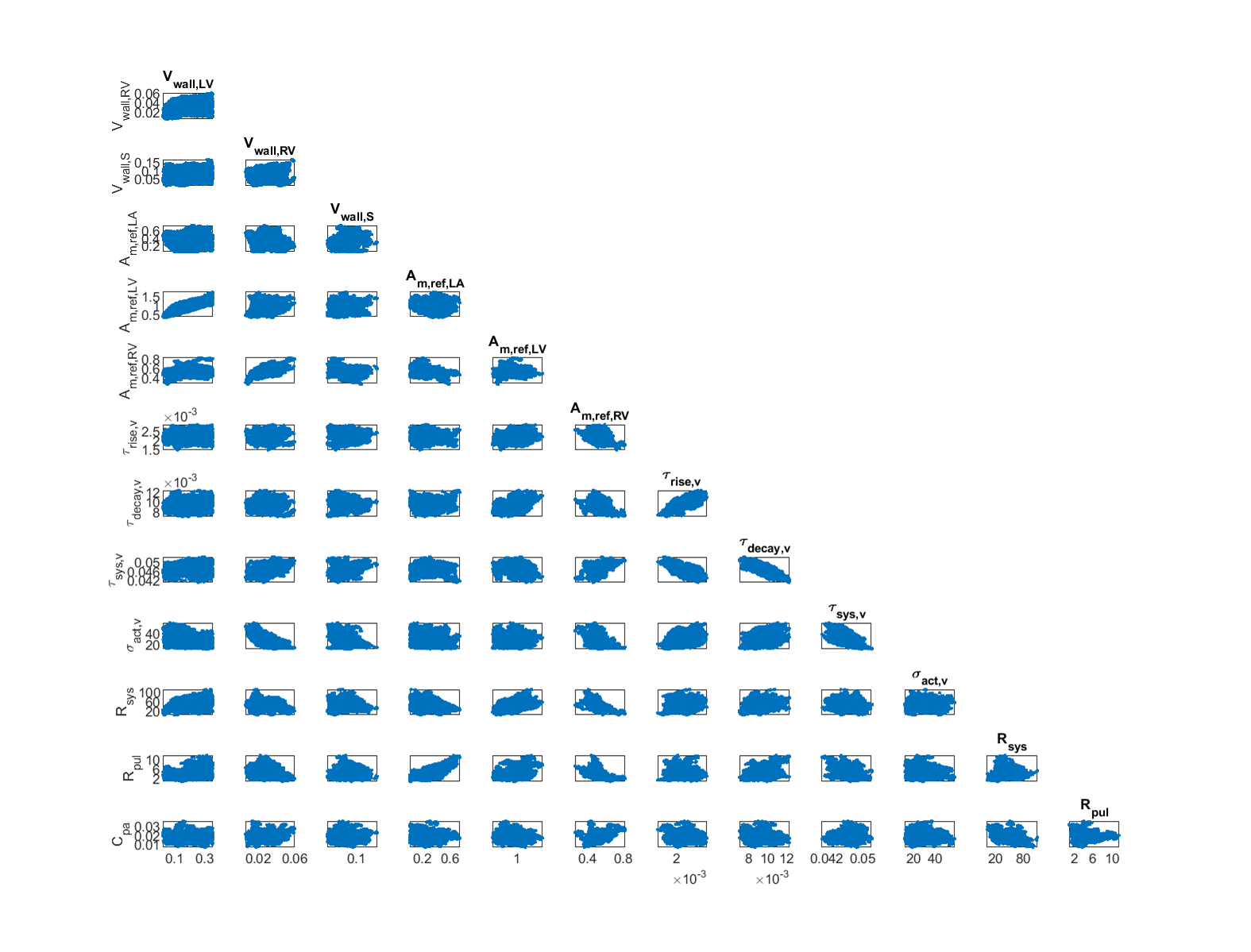


Figure S8: Residual 1 – Iteration 6


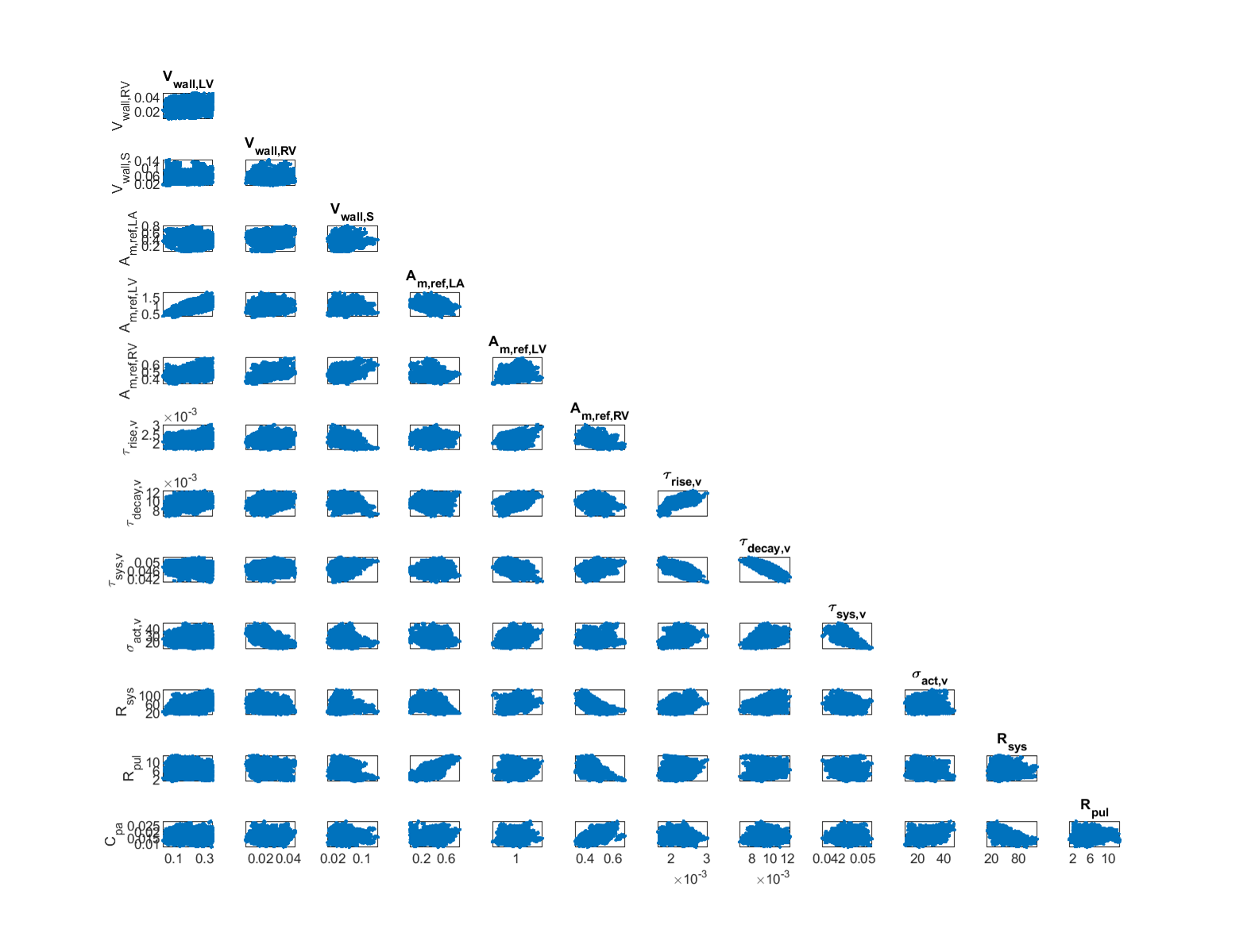


Figure S9: Residual 1 – Iteration 7


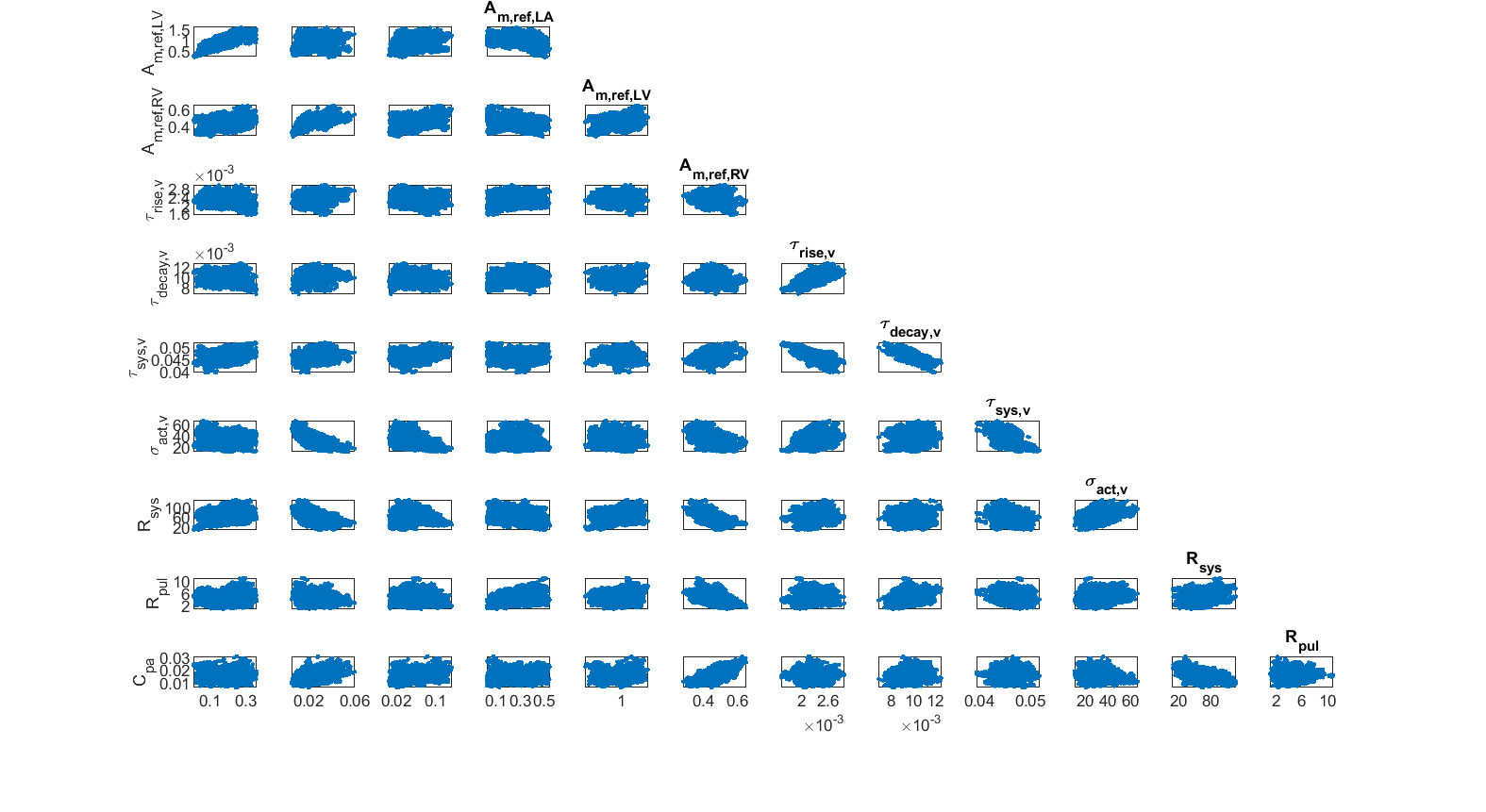

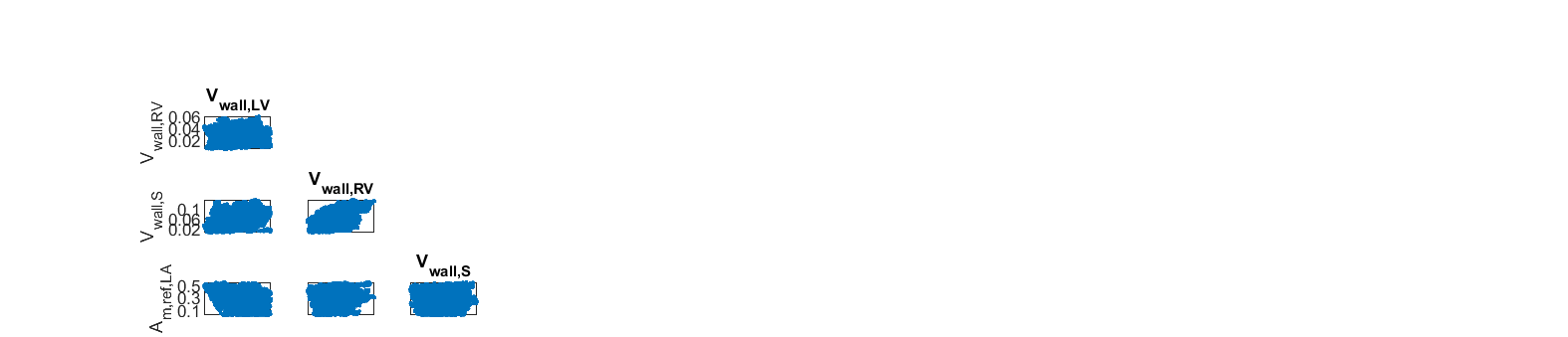


Figure S10: Residual 1 – Iteration 8

Figure S11: Residual 1 – Iteration 9


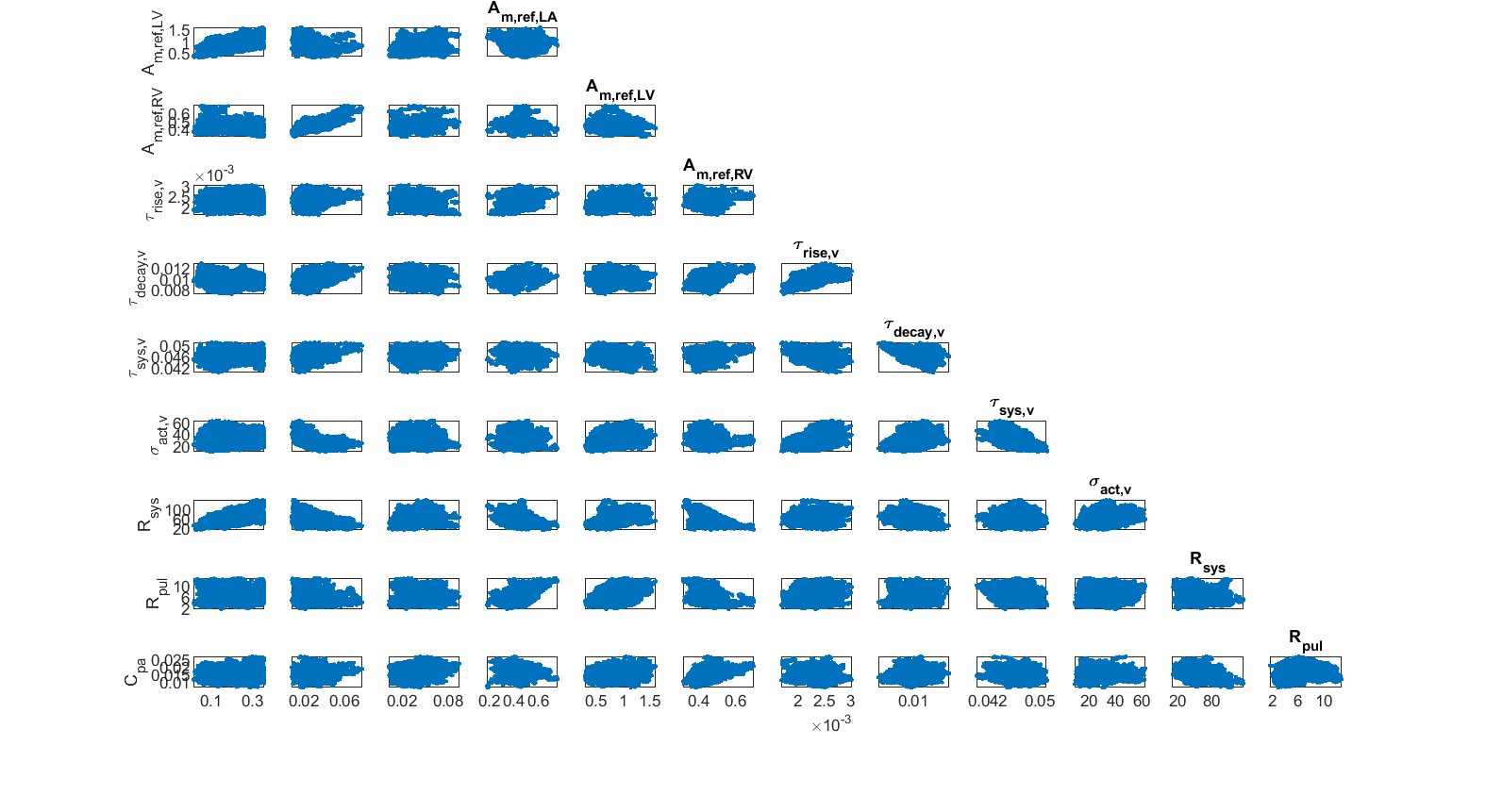

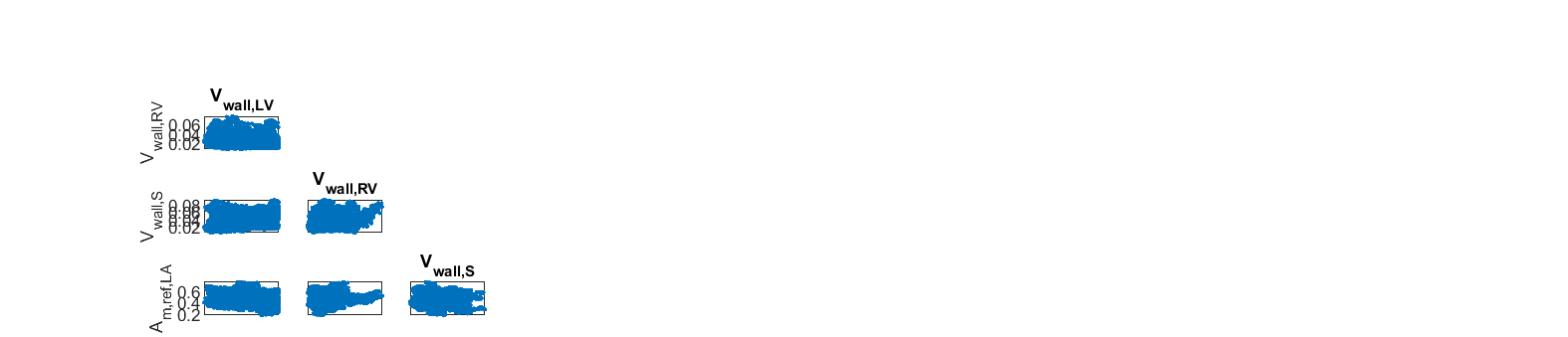


Figure S12: Residual 1 – Iteration 10


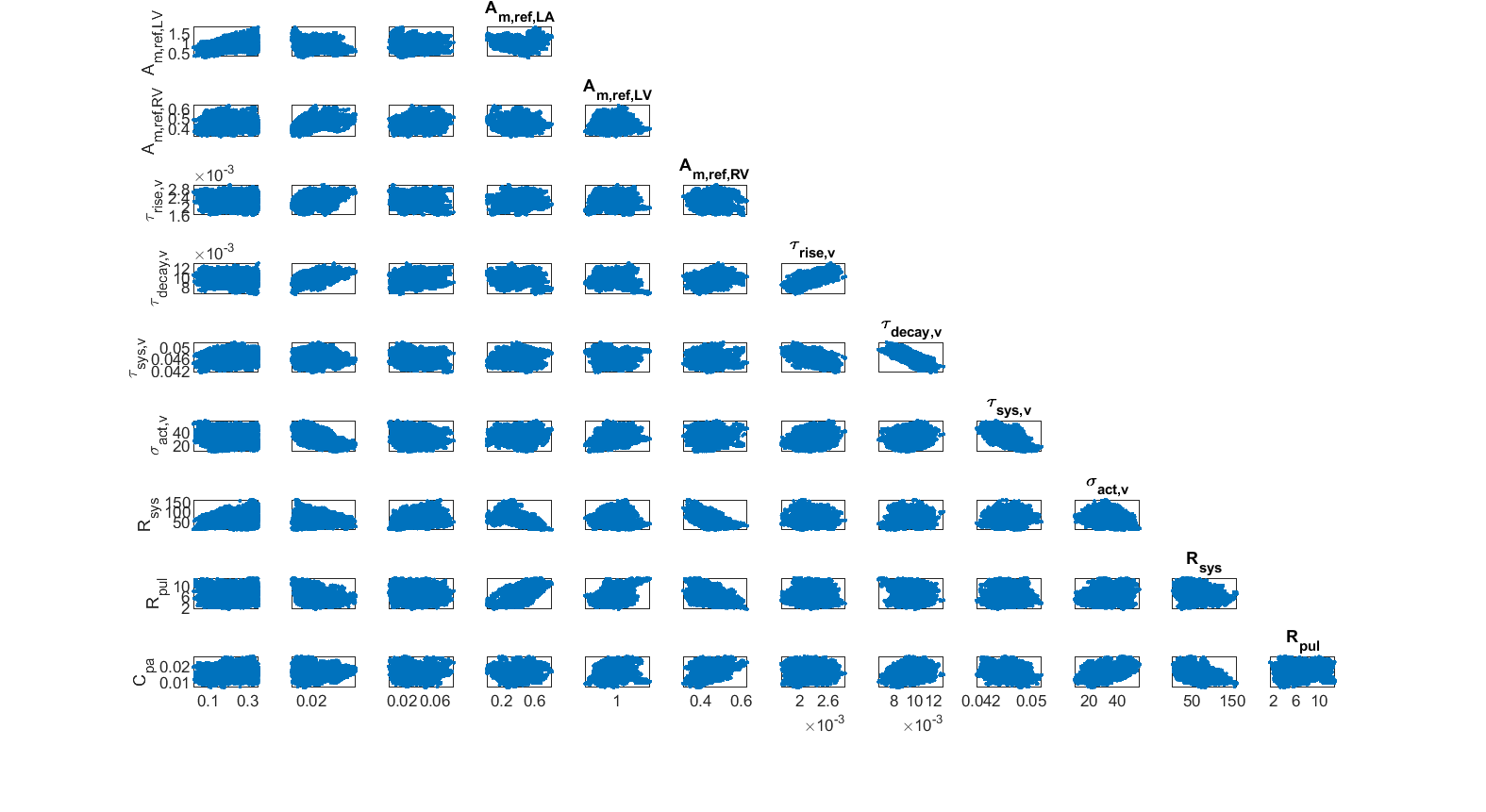

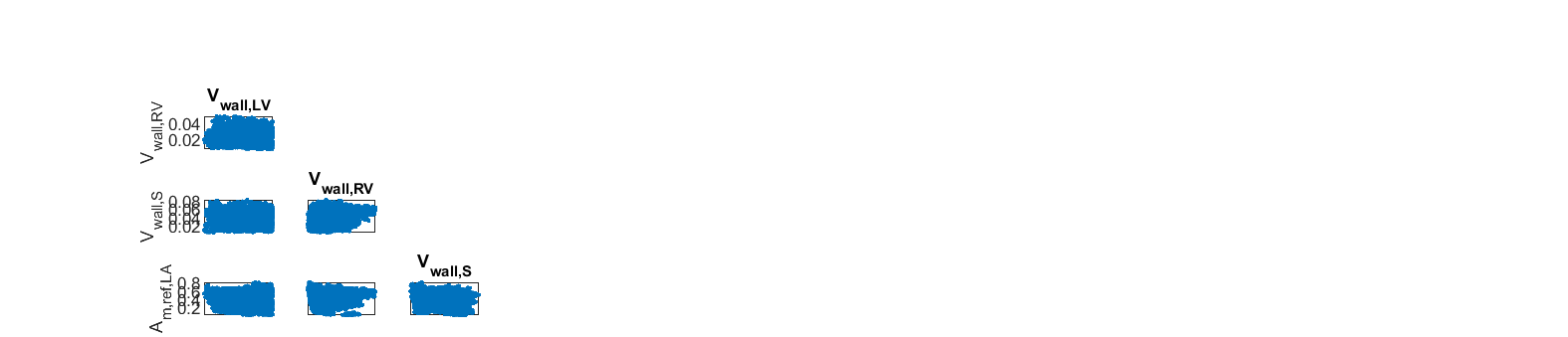


Figure S13: Residual 1 – Iteration 11


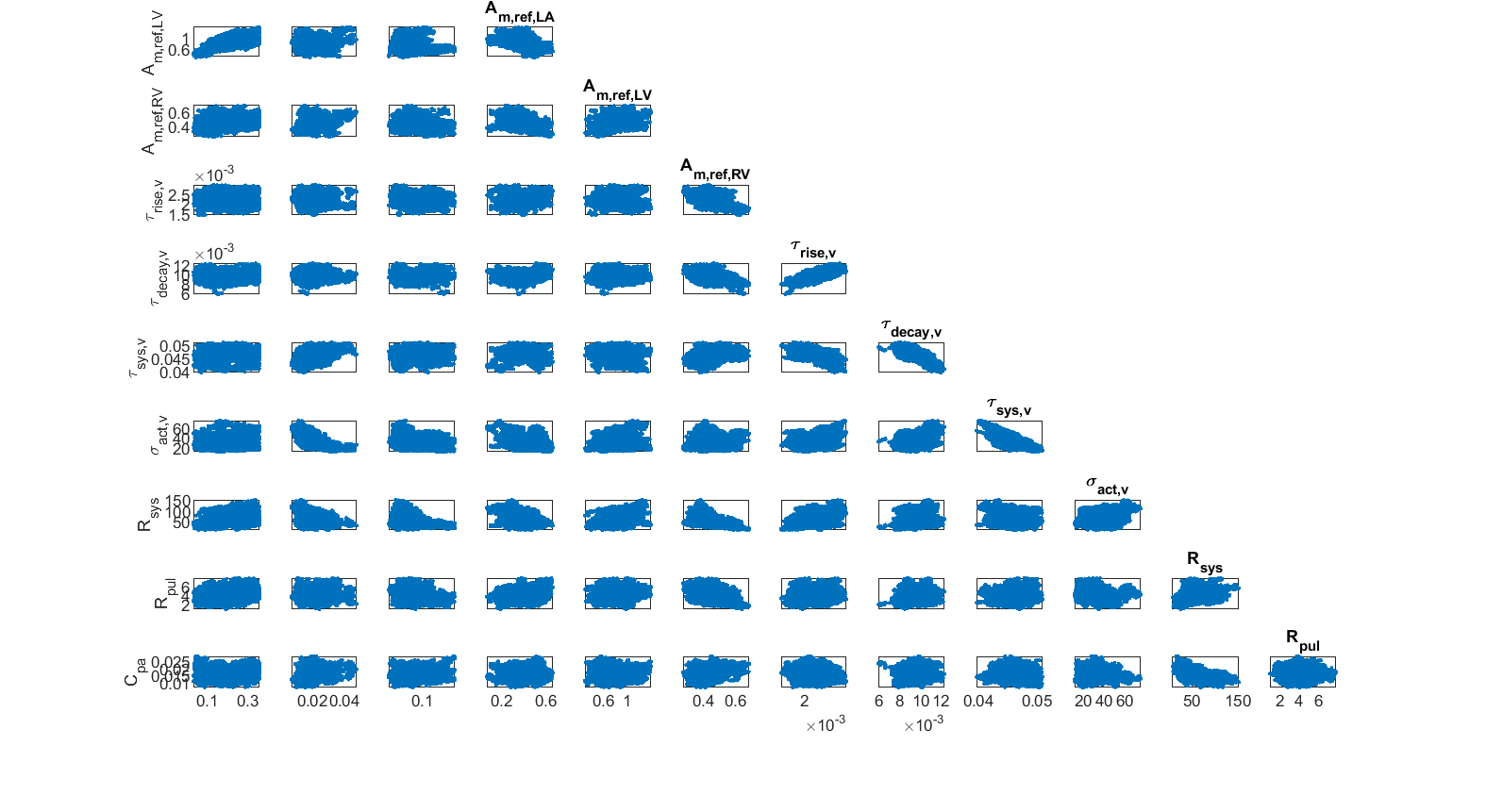

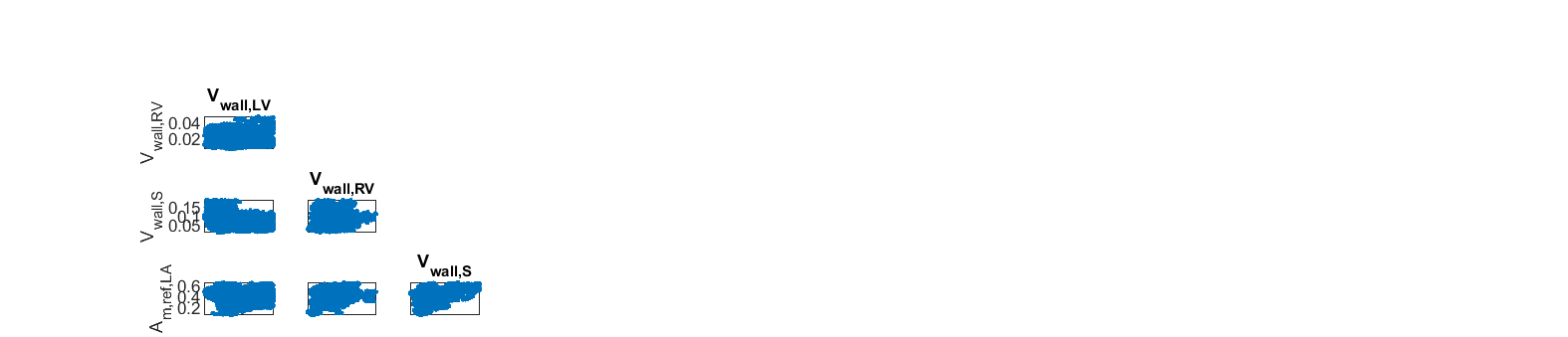

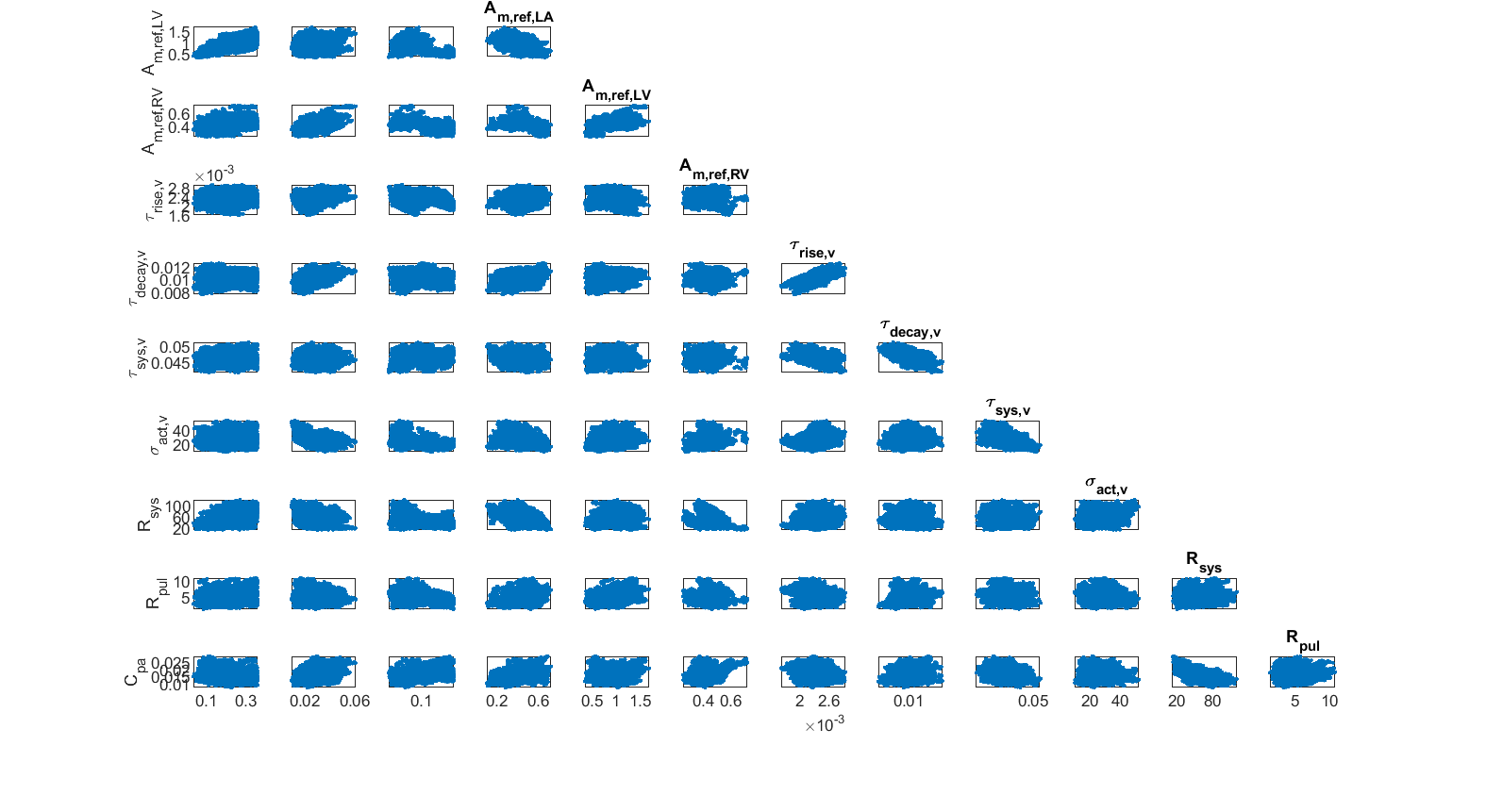

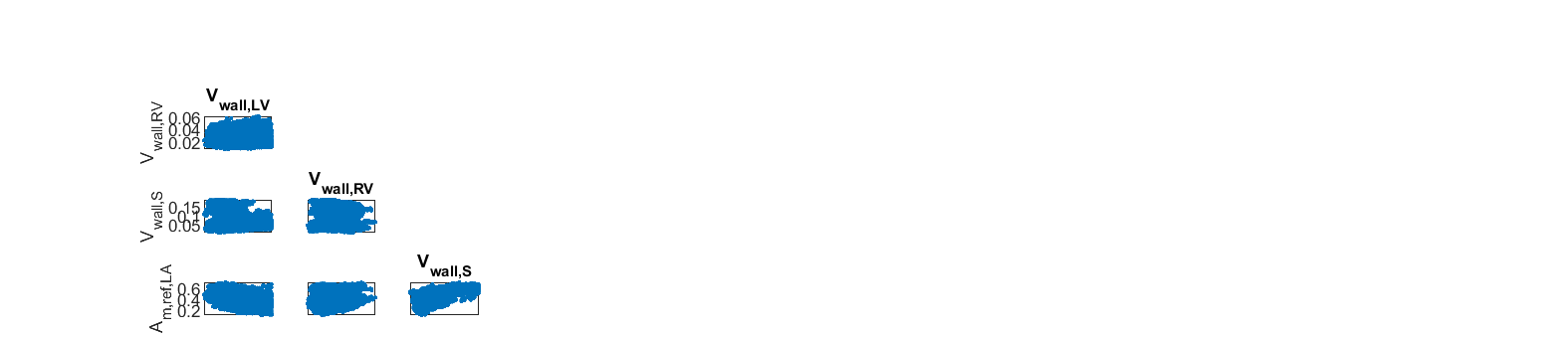


Figure S14: Residual 1 – Iteration 12


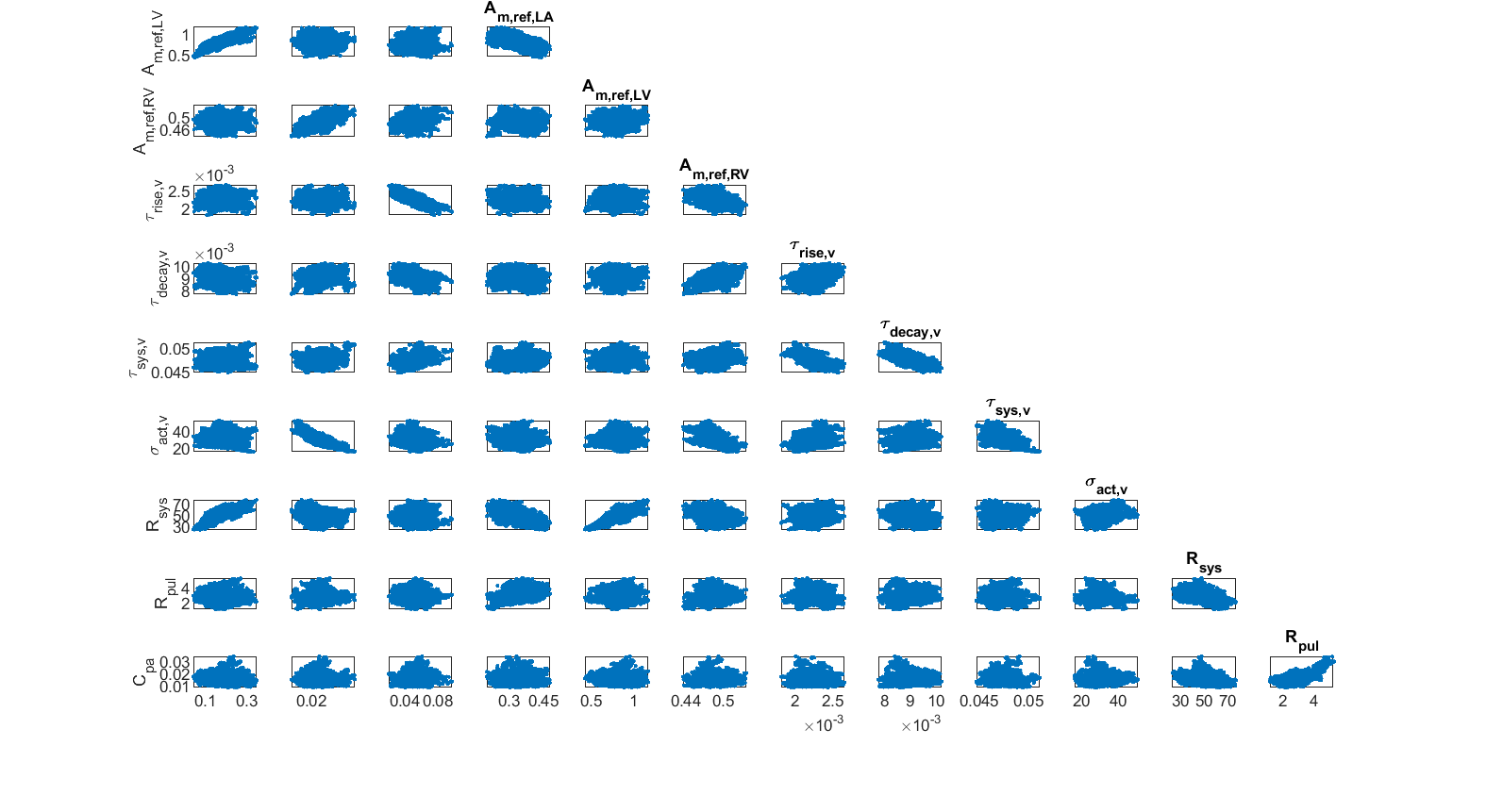

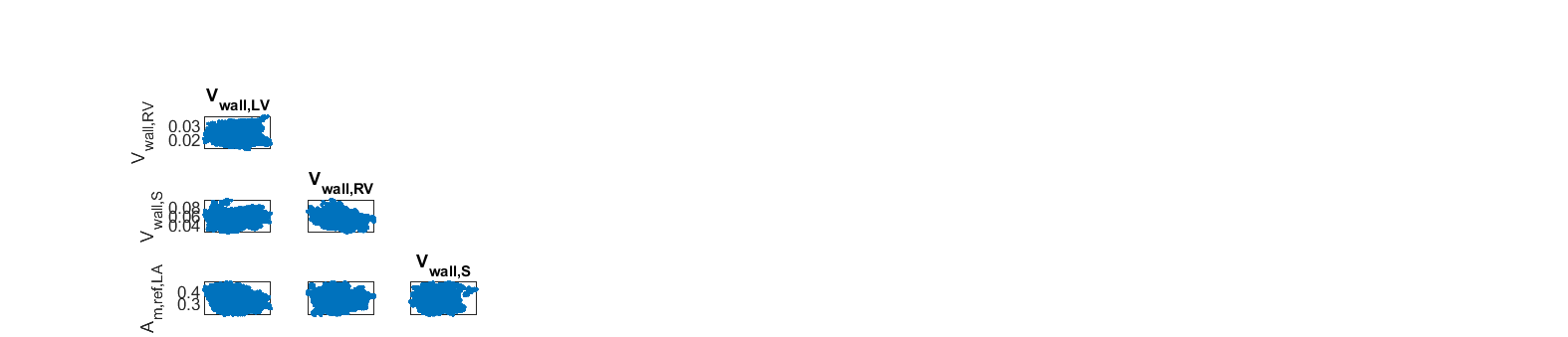


Figure S15: Residual 2 – Iteration 1


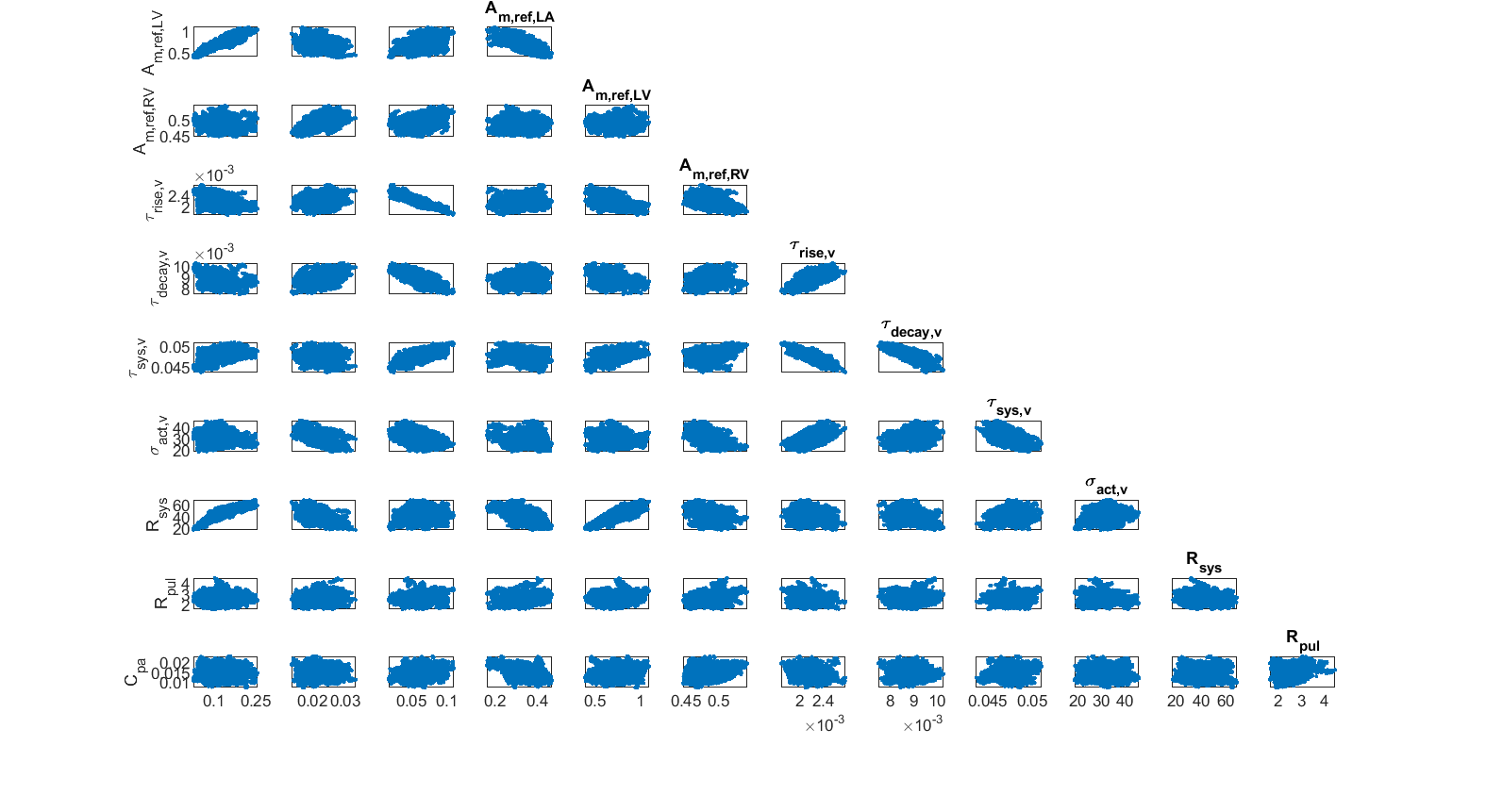

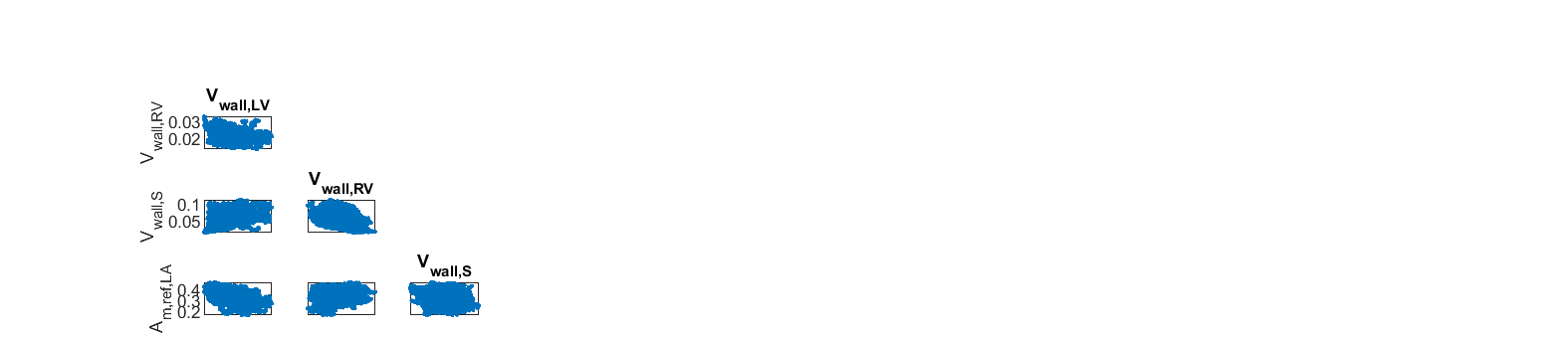


Figure S16: Residual 2 – Iteration 2


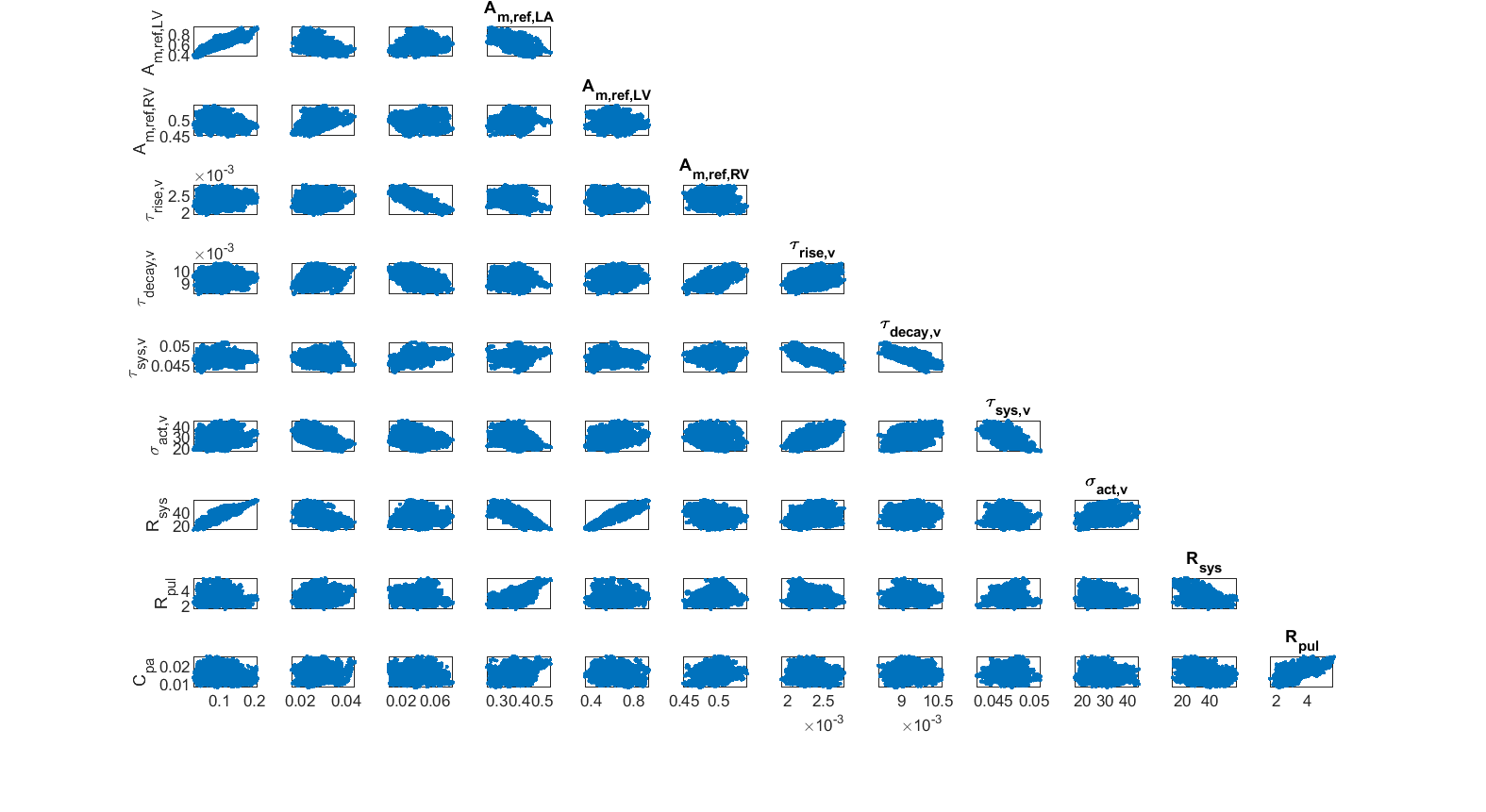

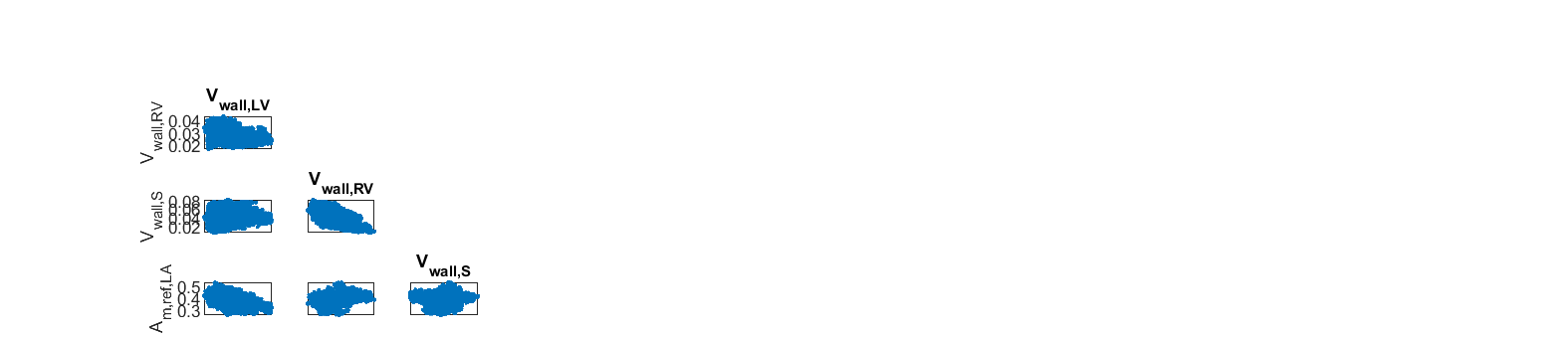


Figure S17: Residual 2 – Iteration 3


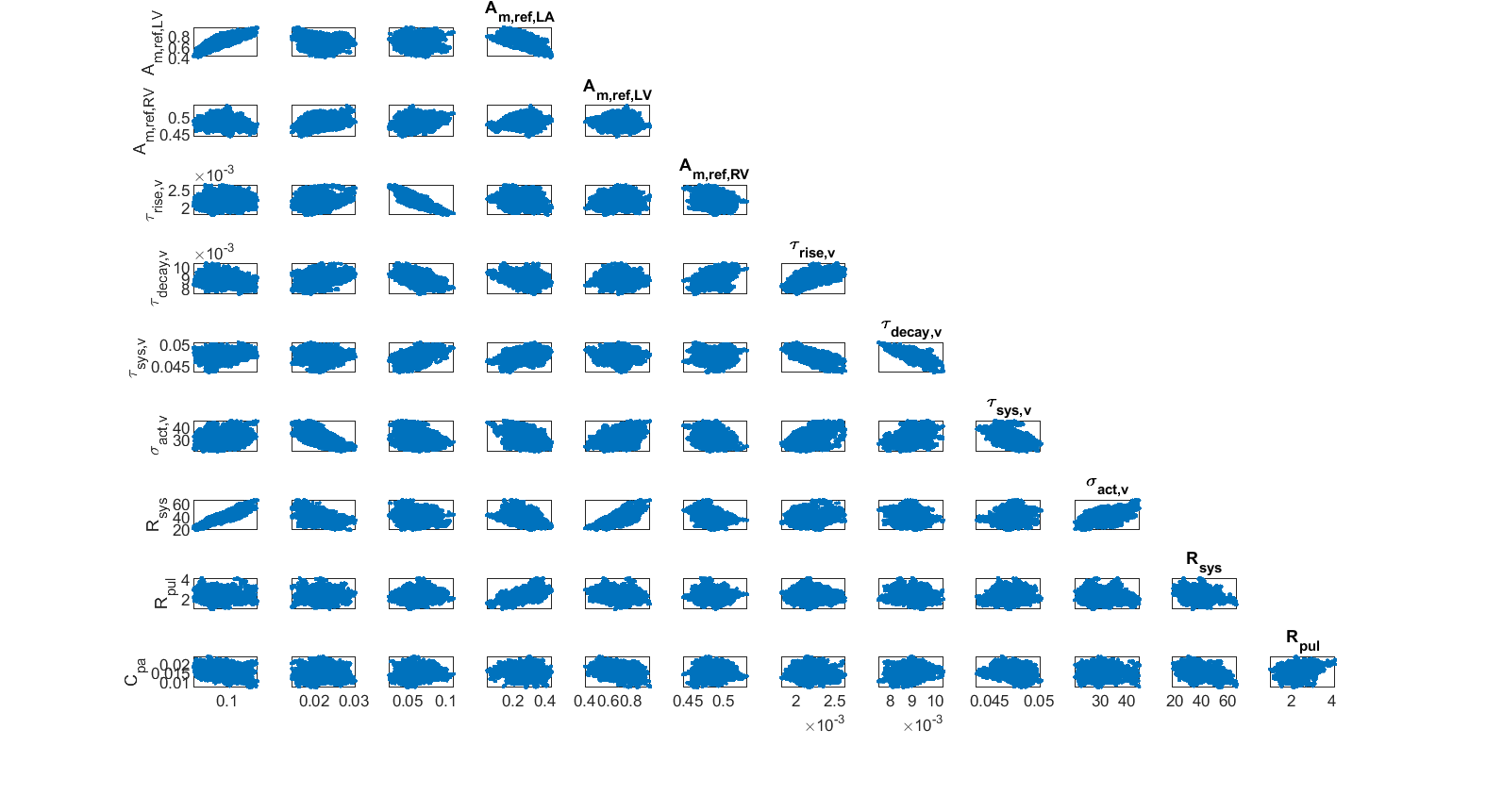

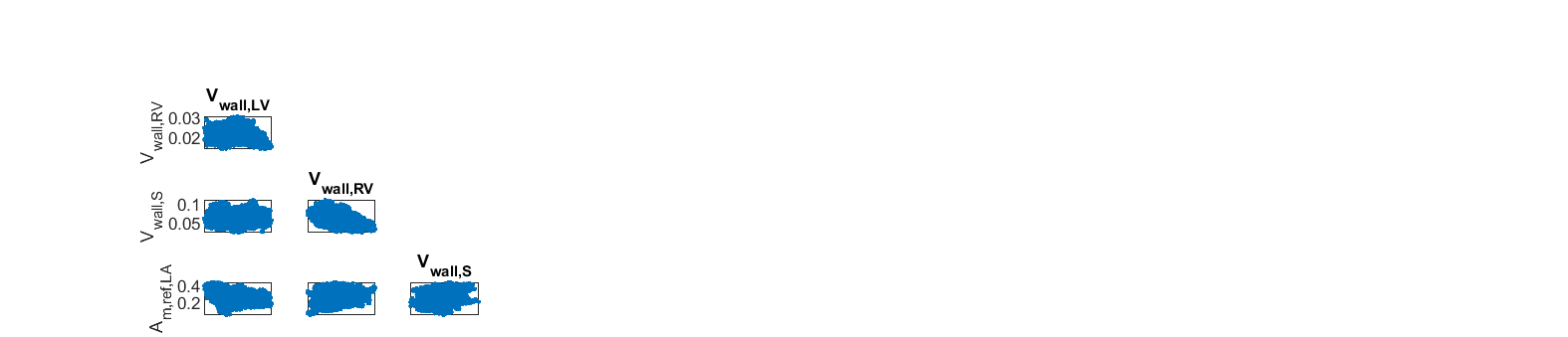


Figure S18: Residual 2 – Iteration 4


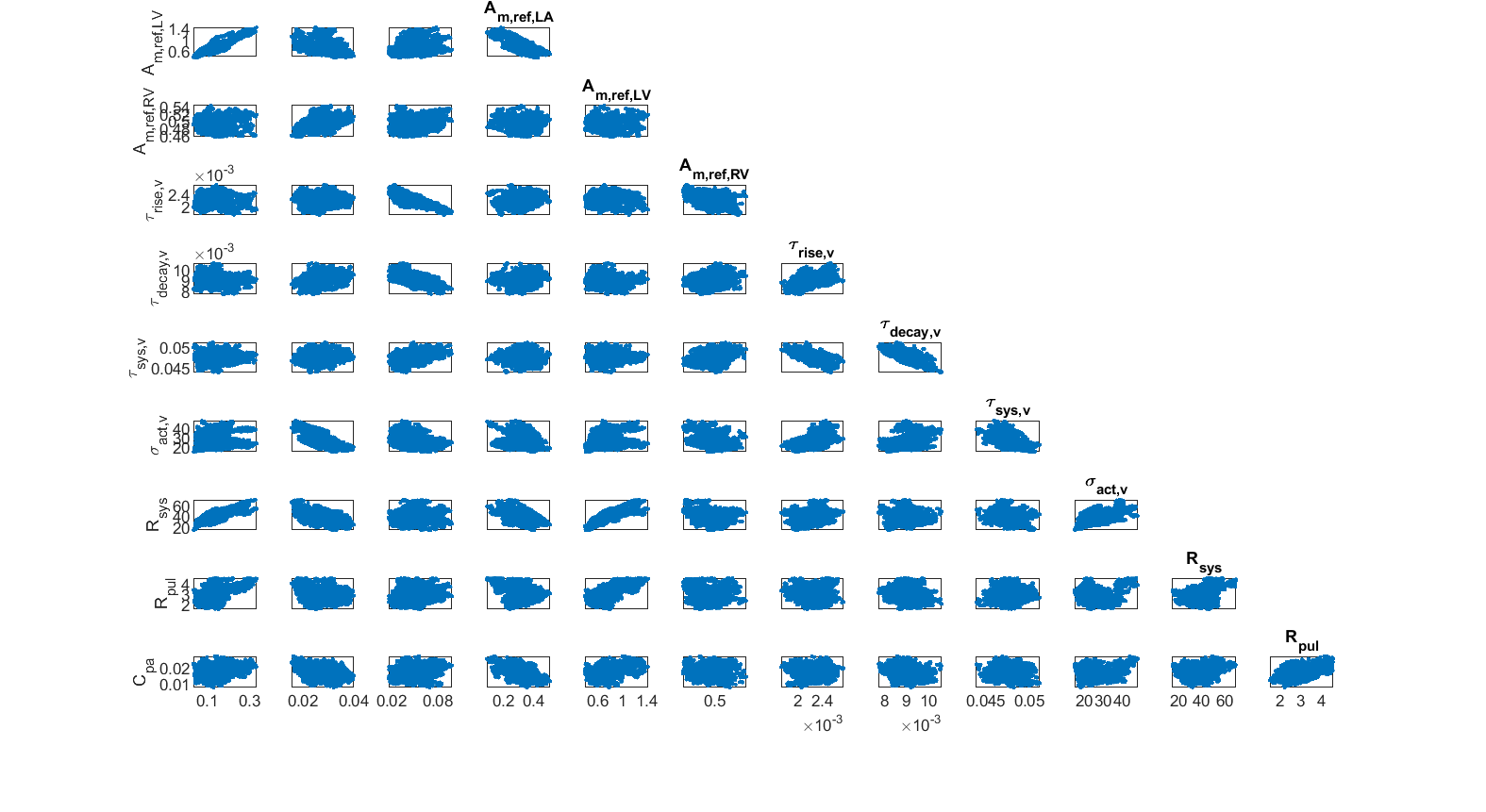

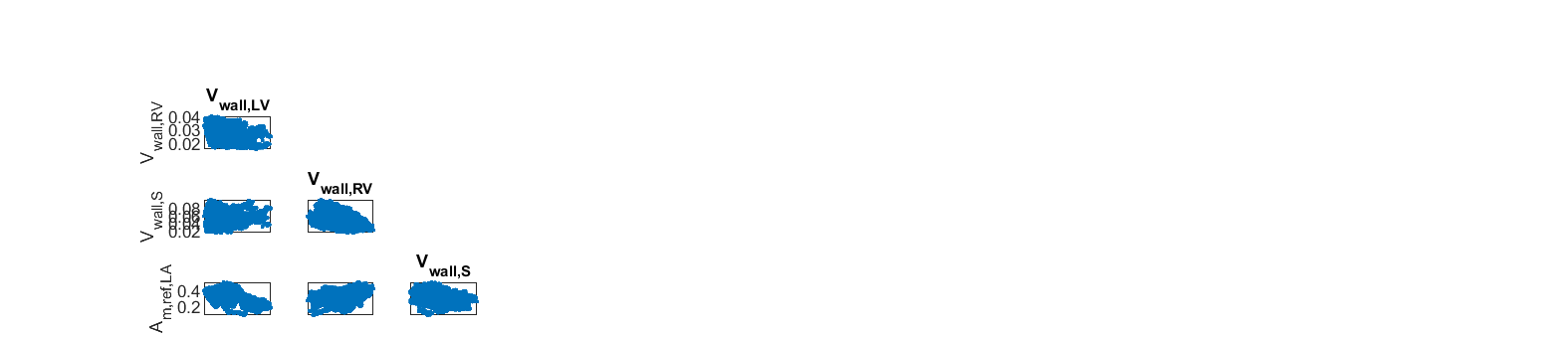


Figure S19: Residual 2 – Iteration 5


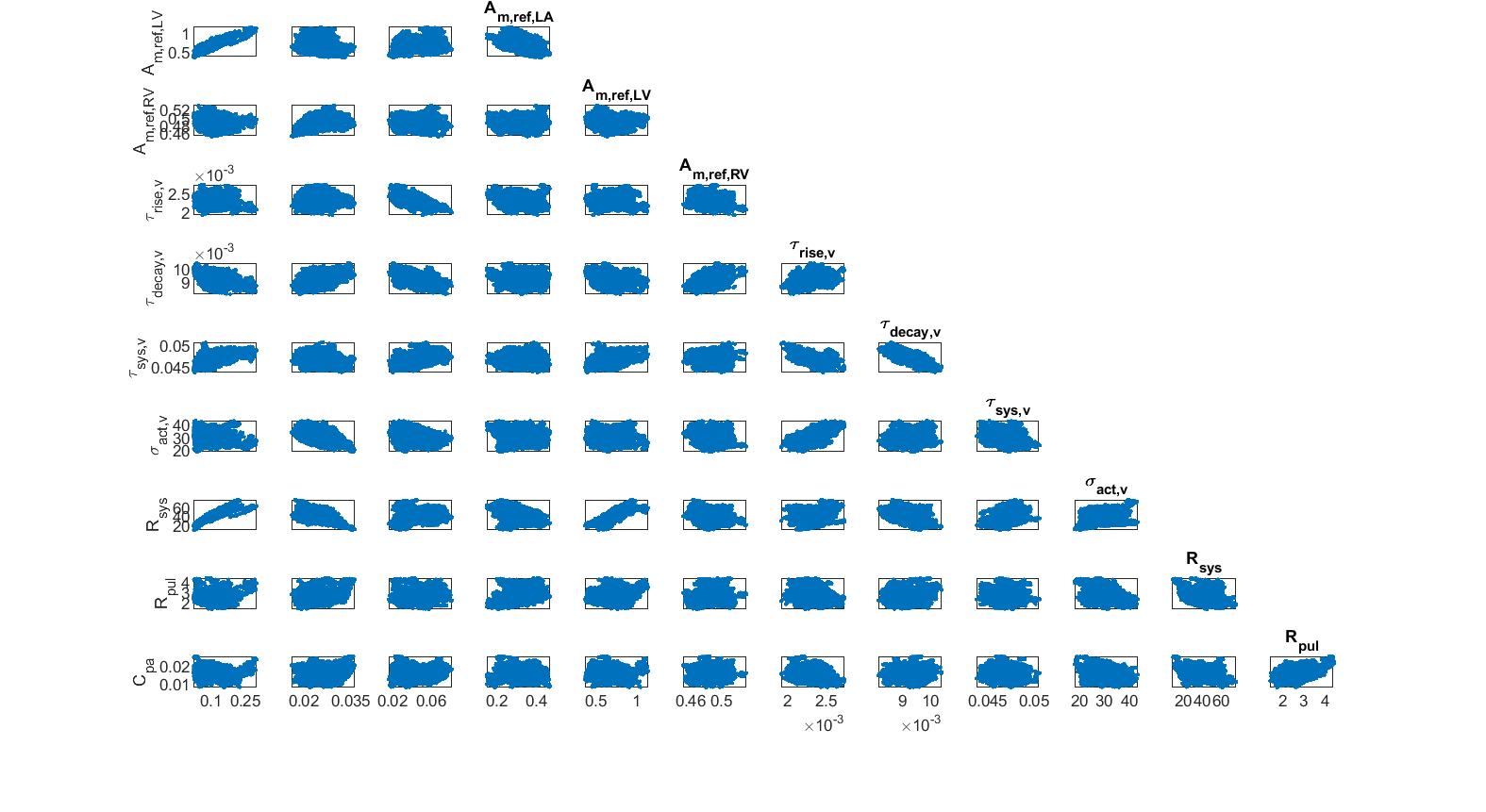

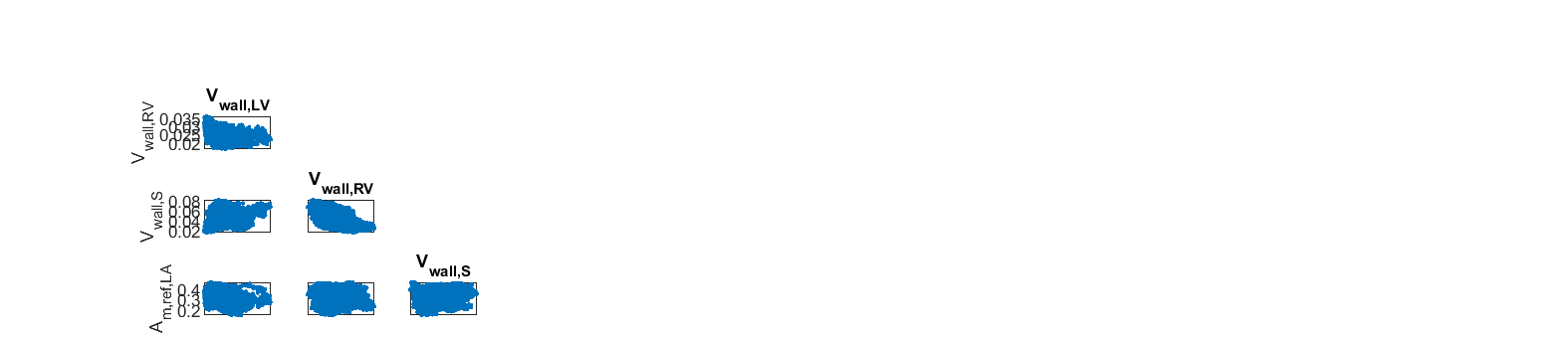


Figure S20: Residual 2 – Iteration 6

Figure S21: Residual 2 – Iteration 7


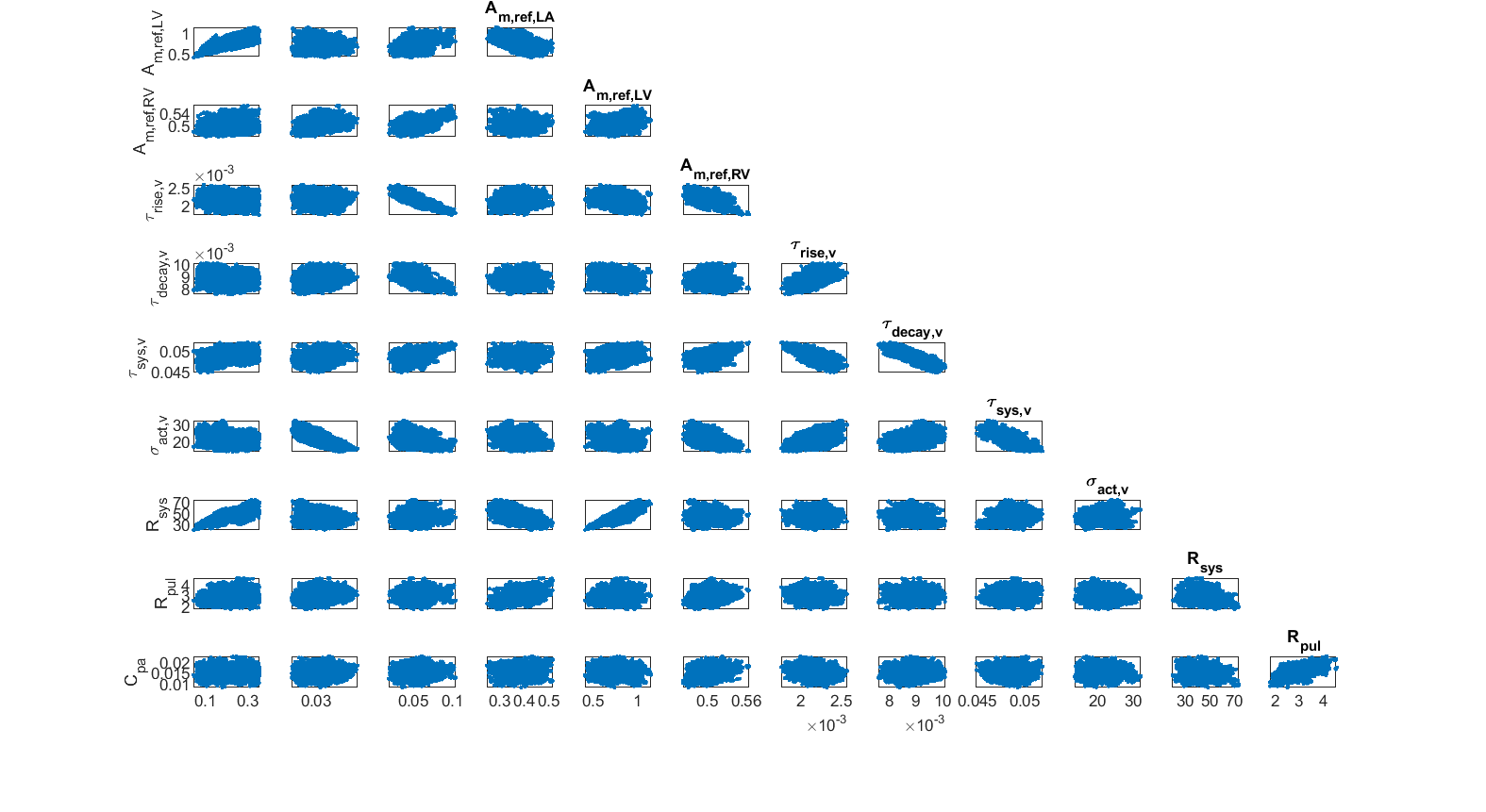

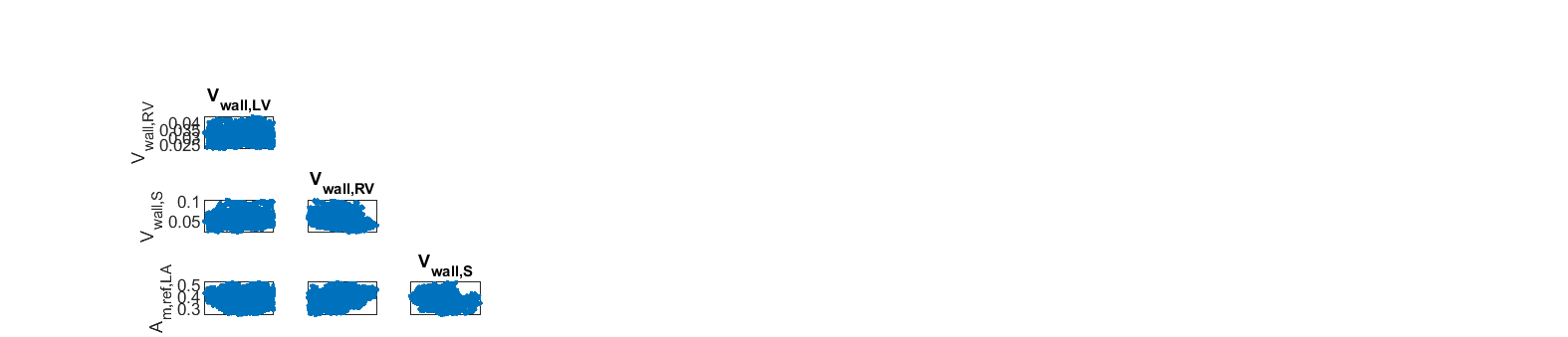

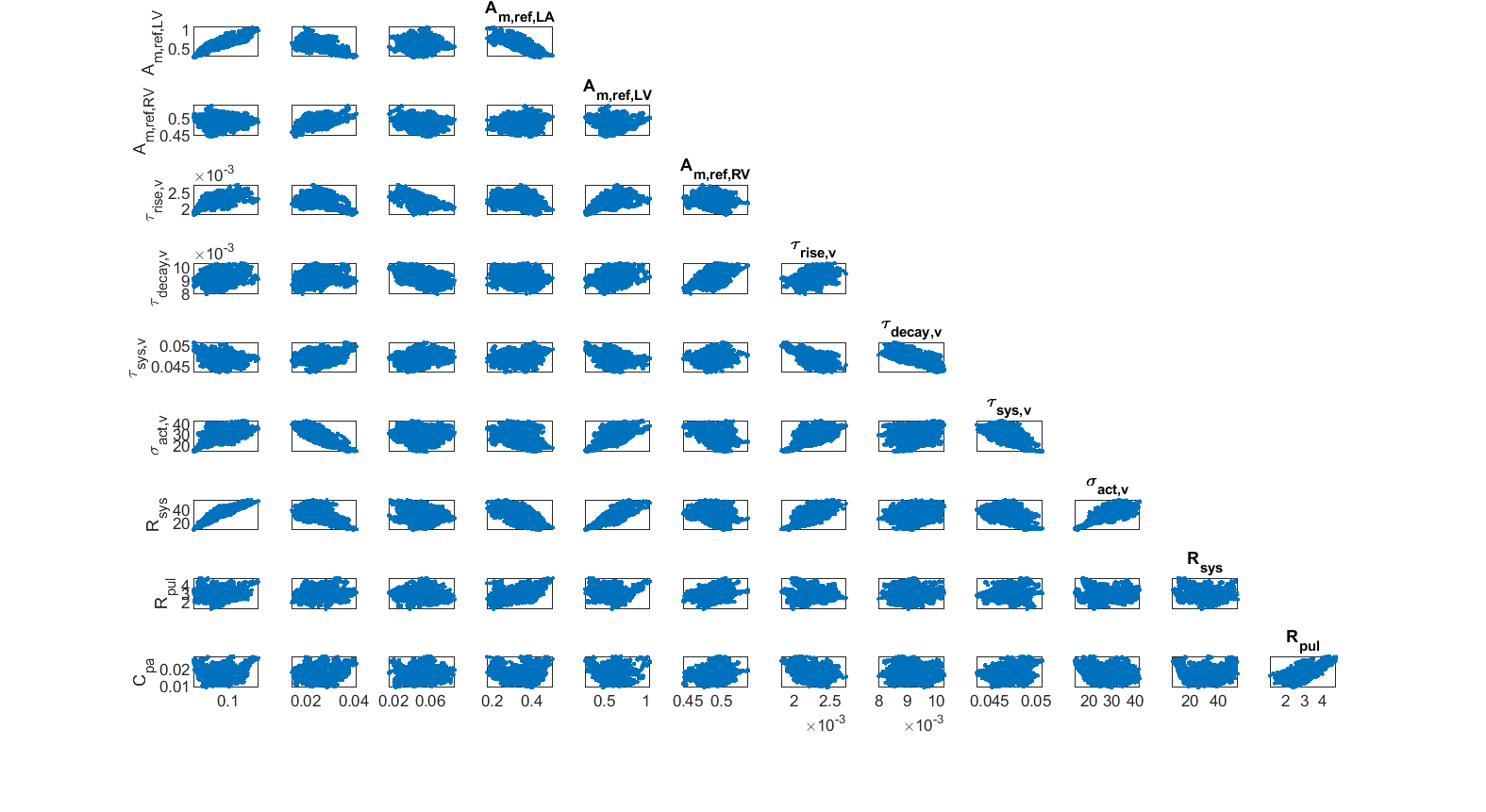

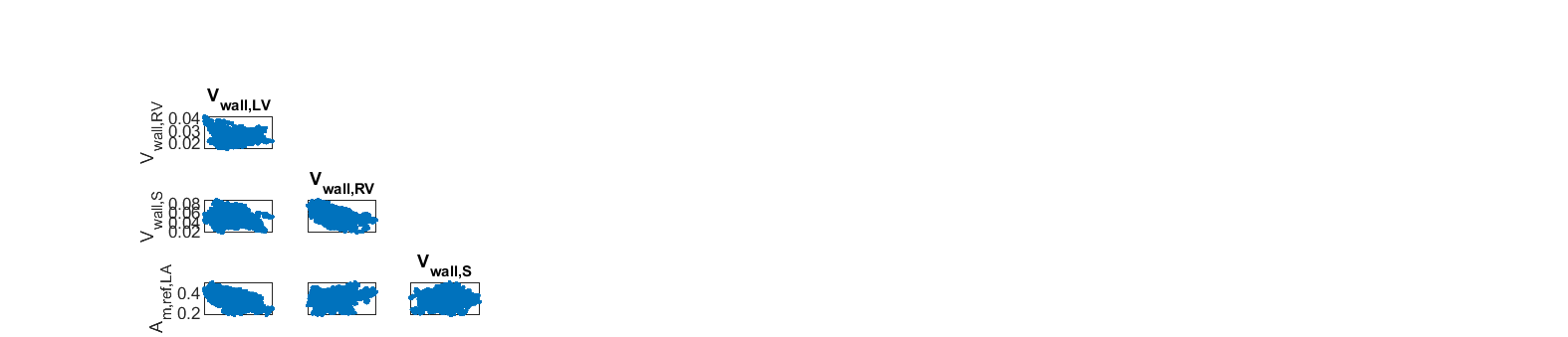


Figure S22: Residual 2 – Iteration 8


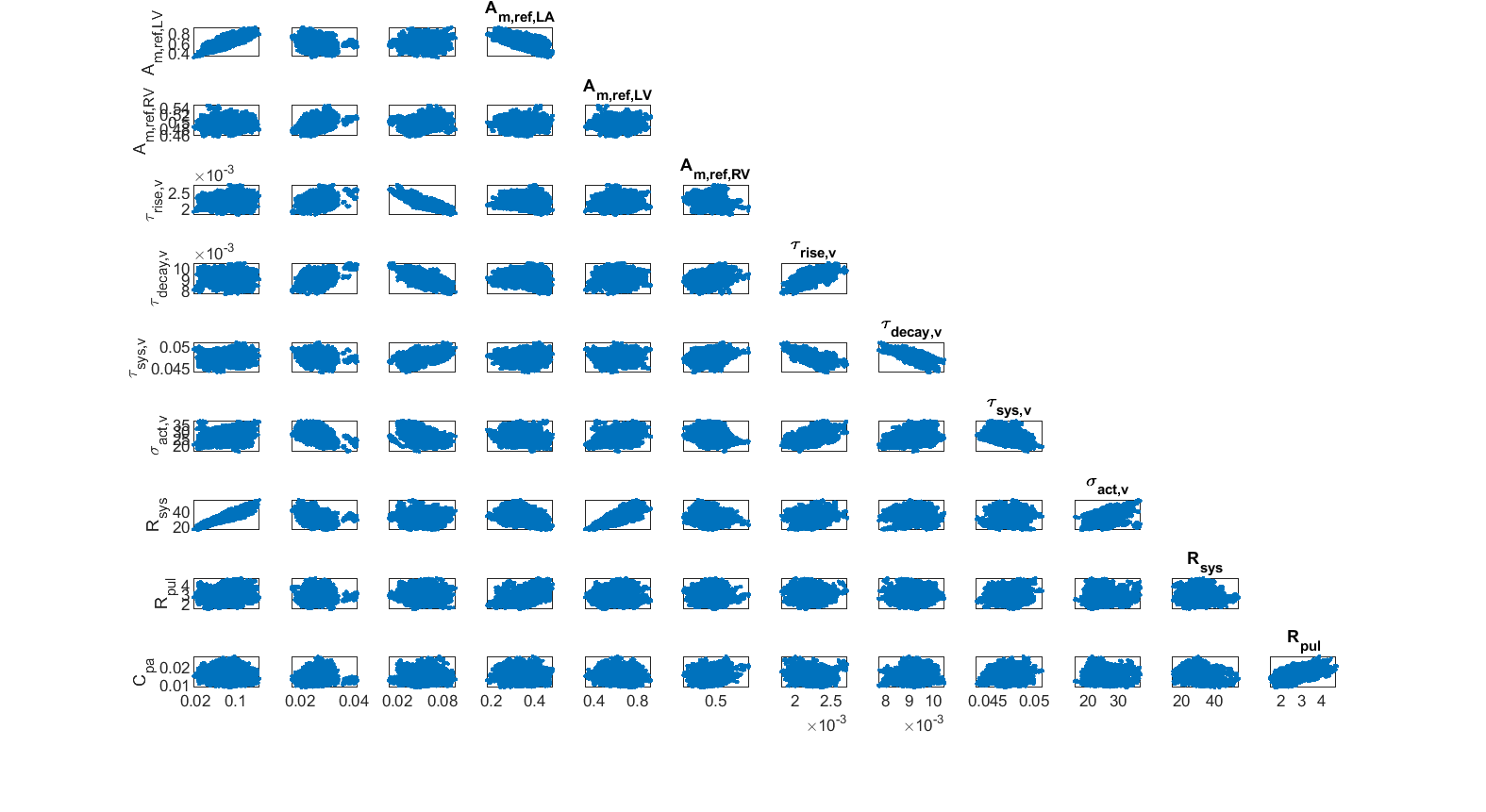

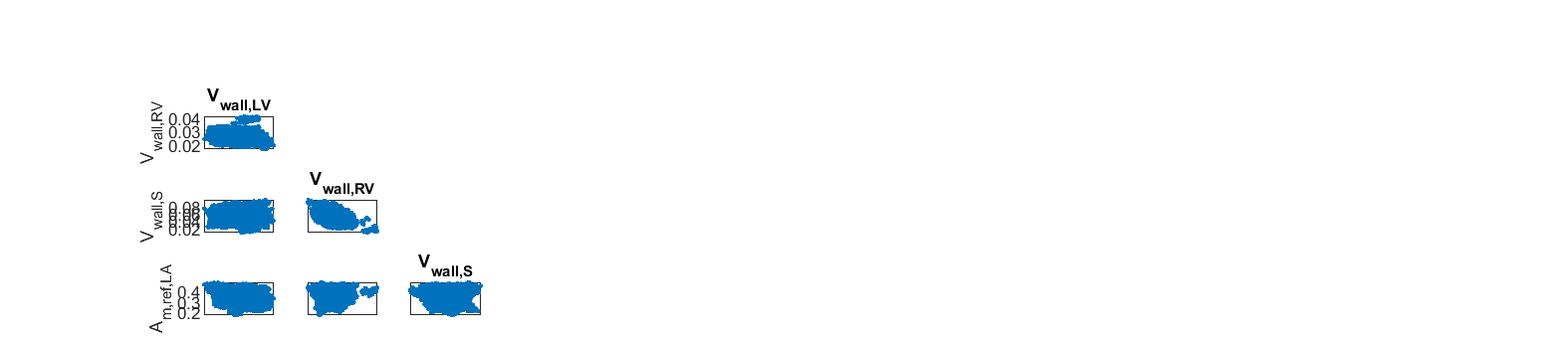


Figure S23: Residual 2 – Iteration 9


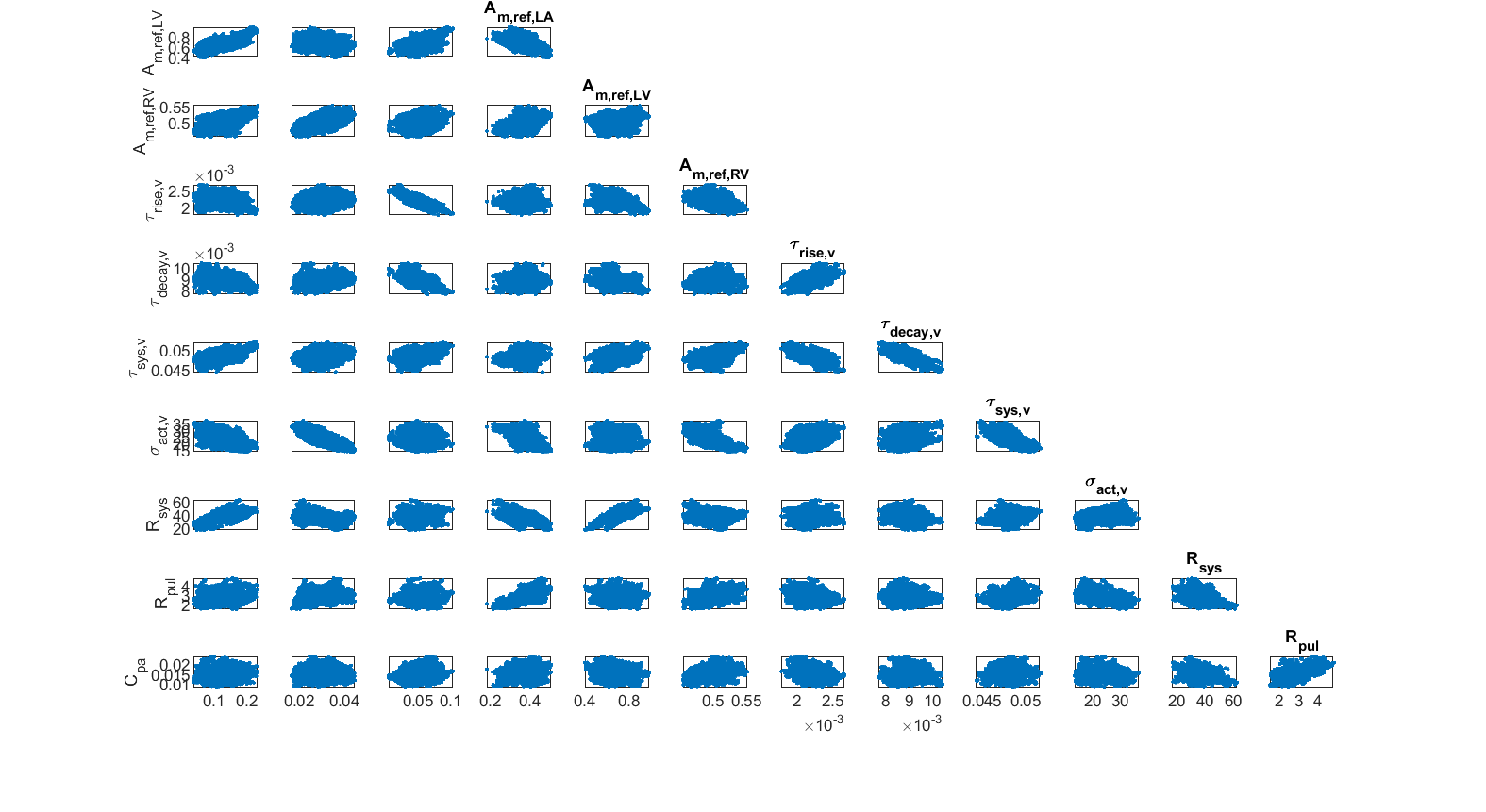

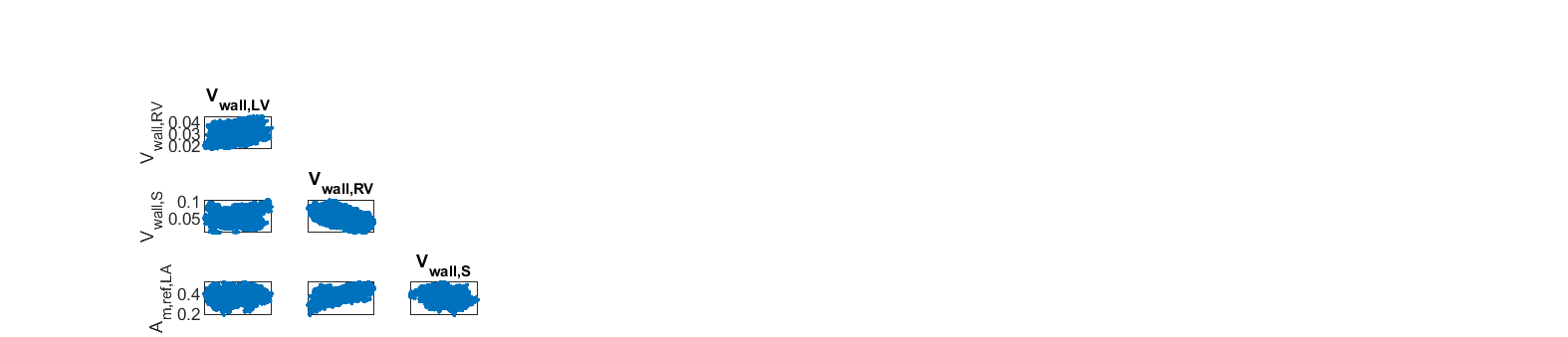


Figure S24: Residual 2 – Iteration 10


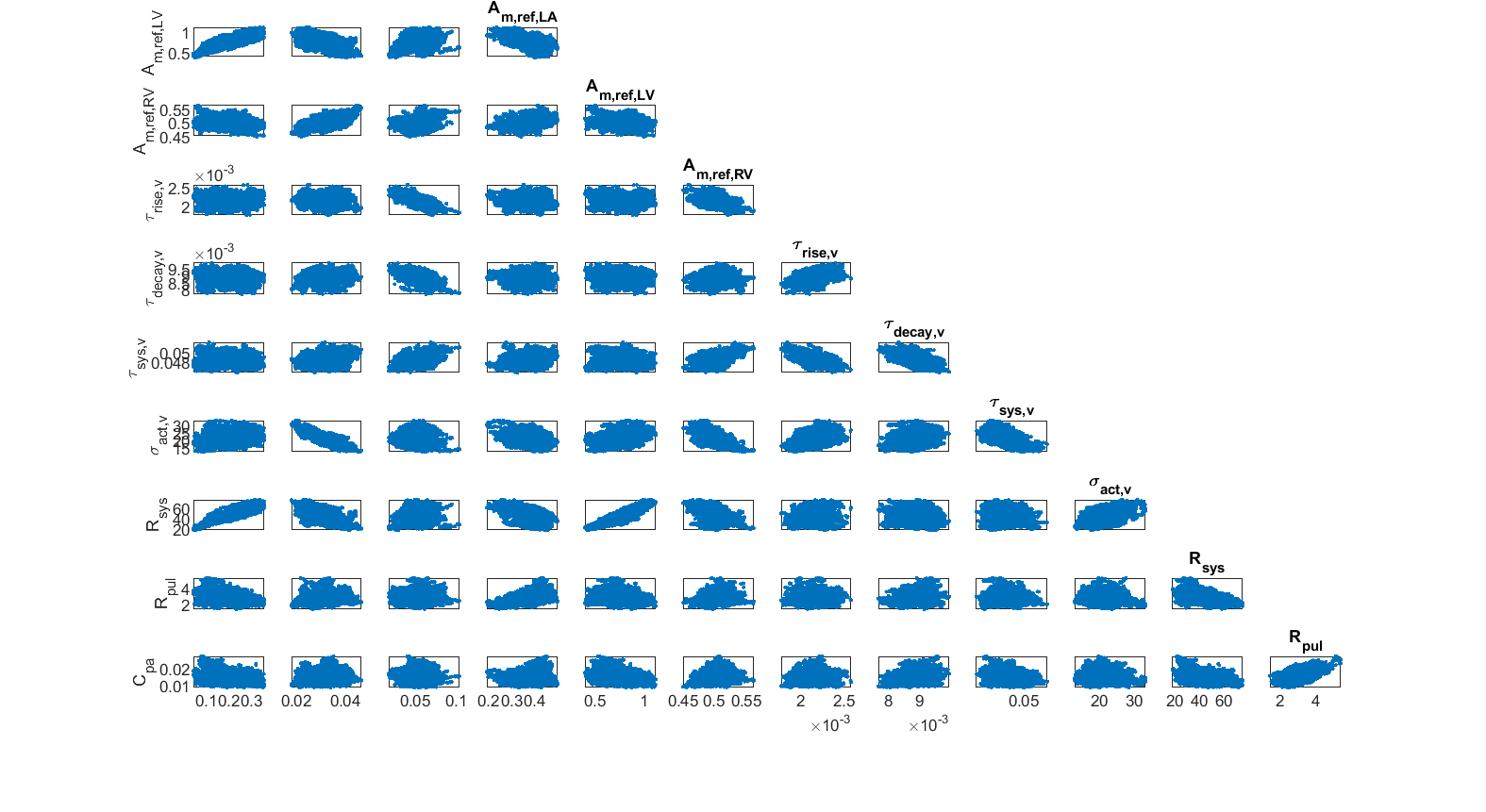

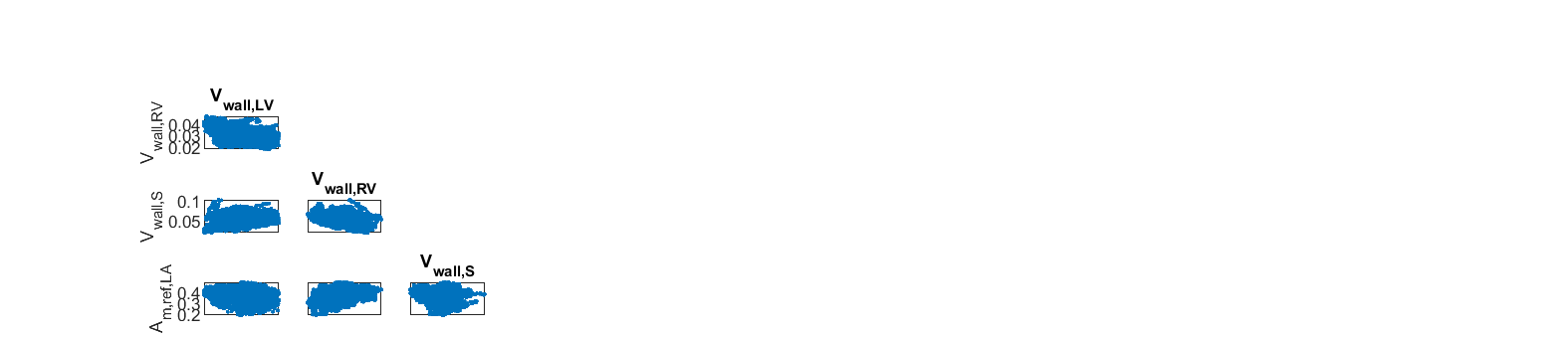


Figure S25: Residual 2 – Iteration 11


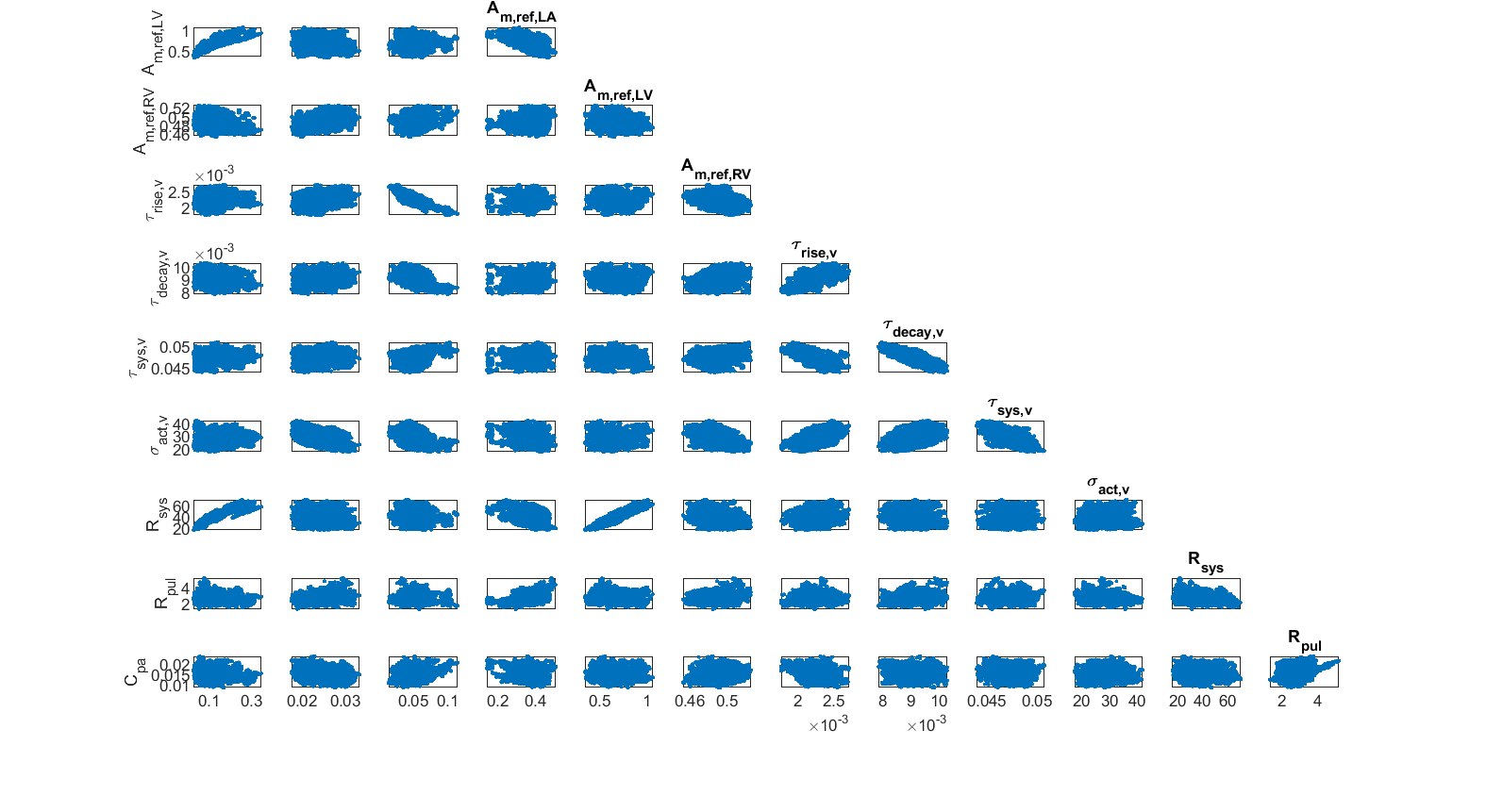

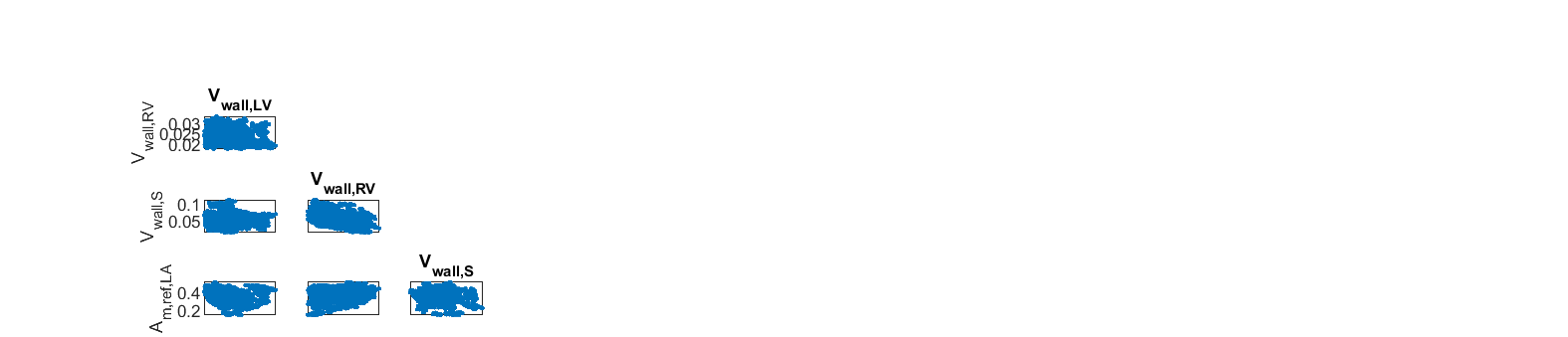


Figure S26: Residual 2 – Iteration 12


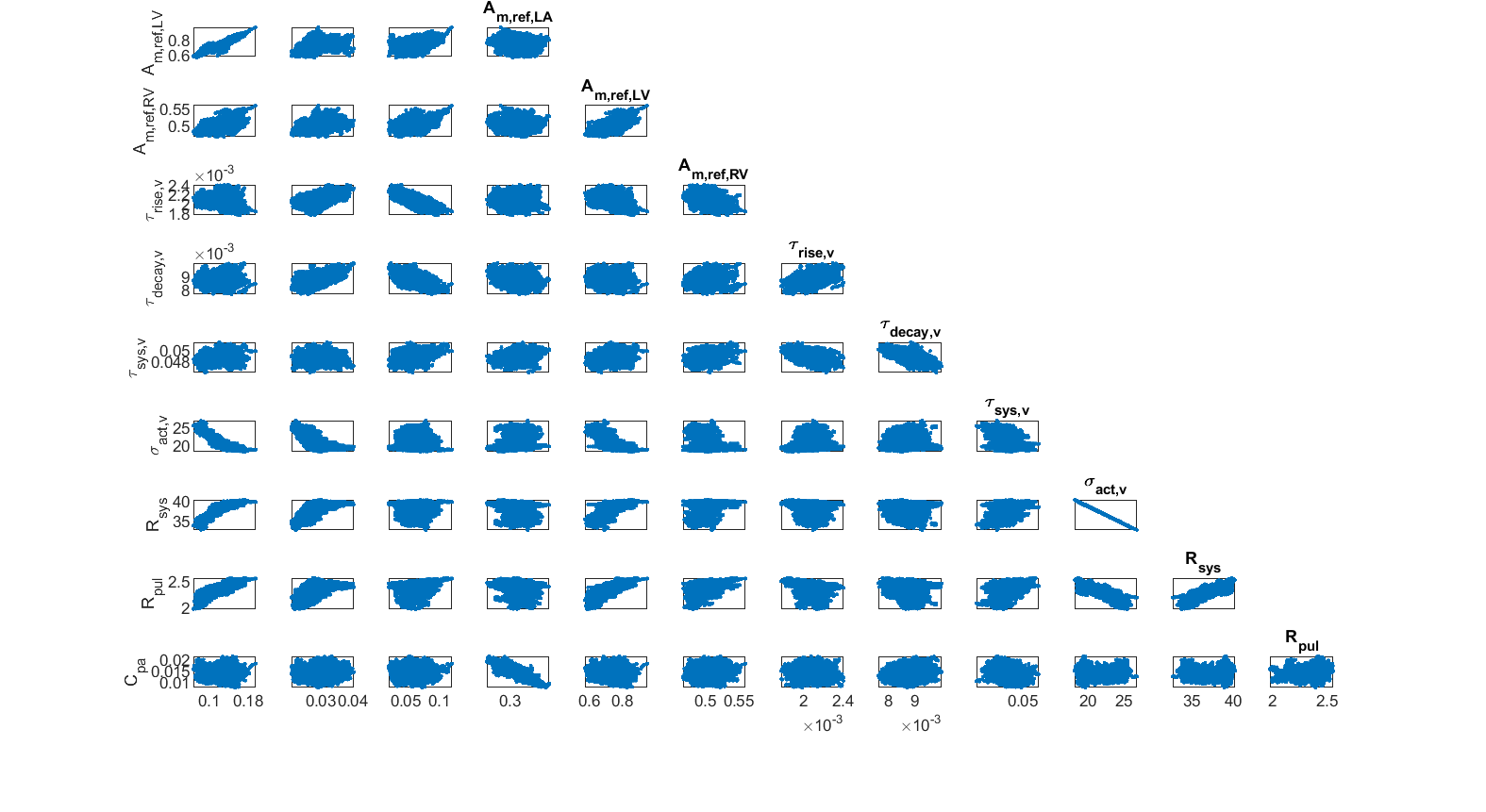

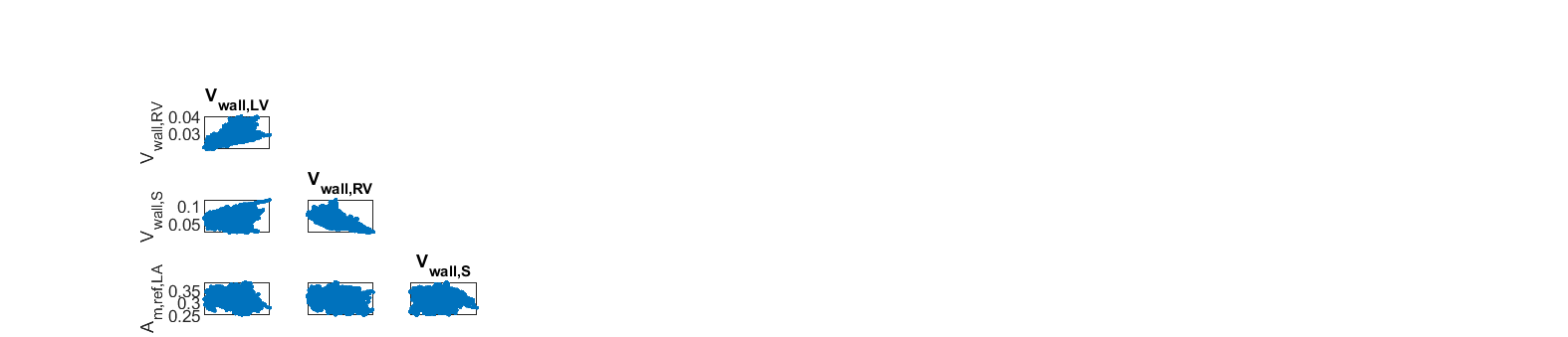


Figure S27: Residual 3 – Iteration 1


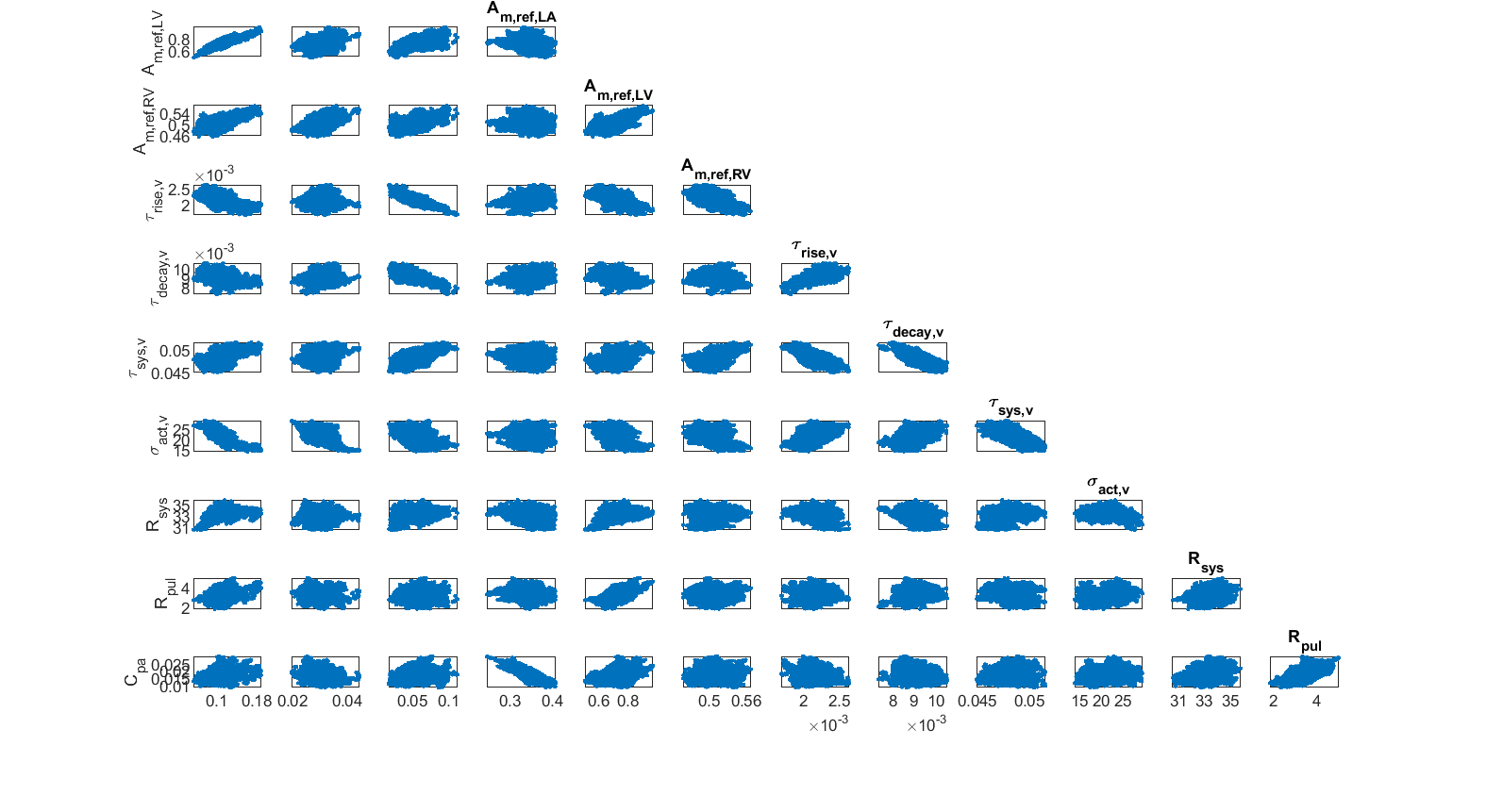

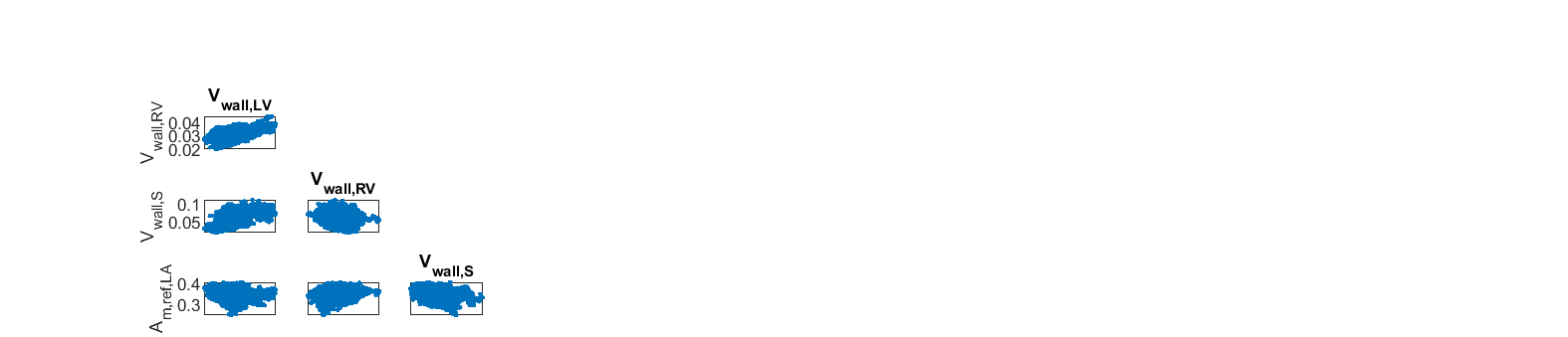


Figure S28: Residual 3 – Iteration 2


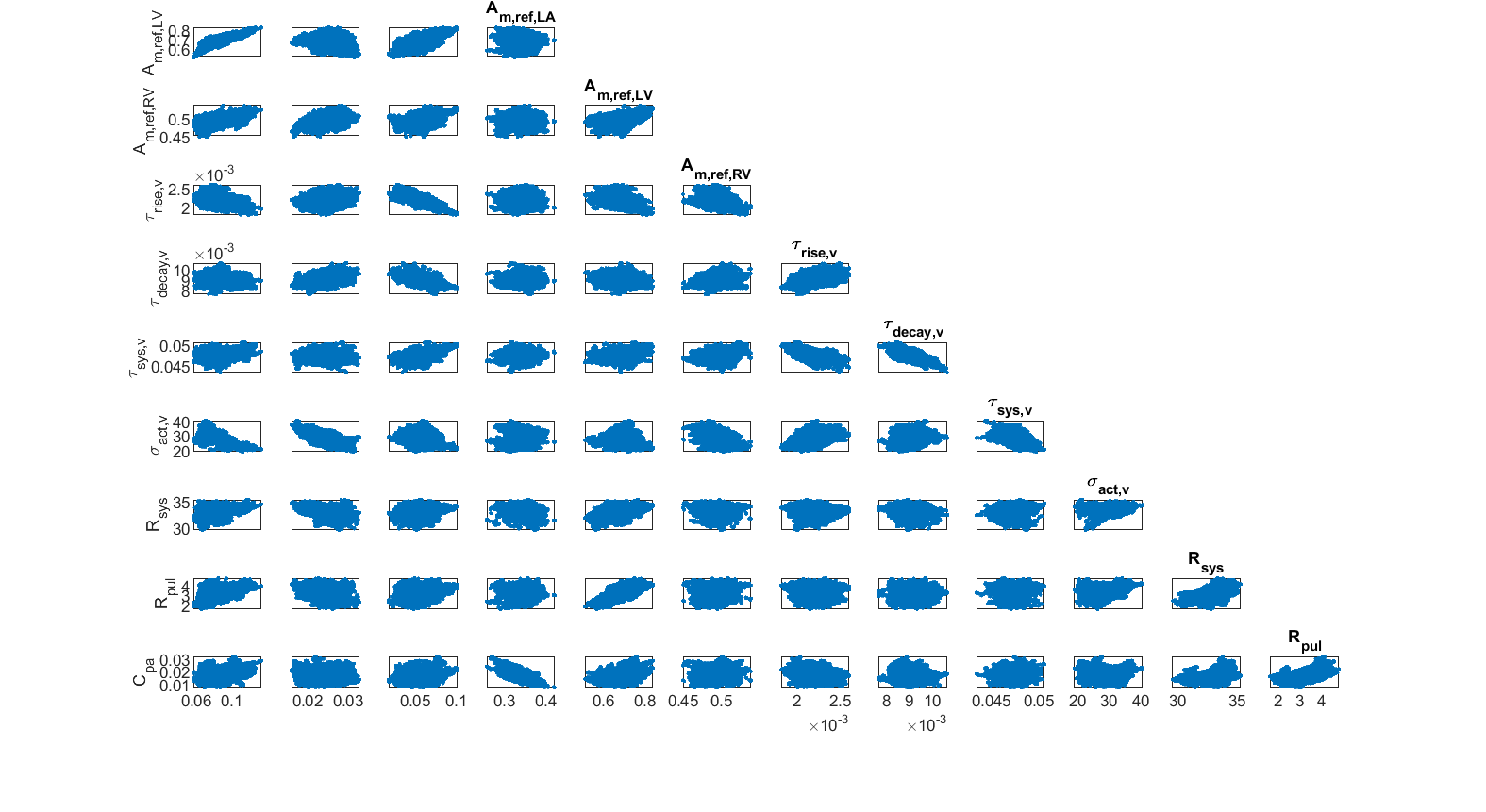

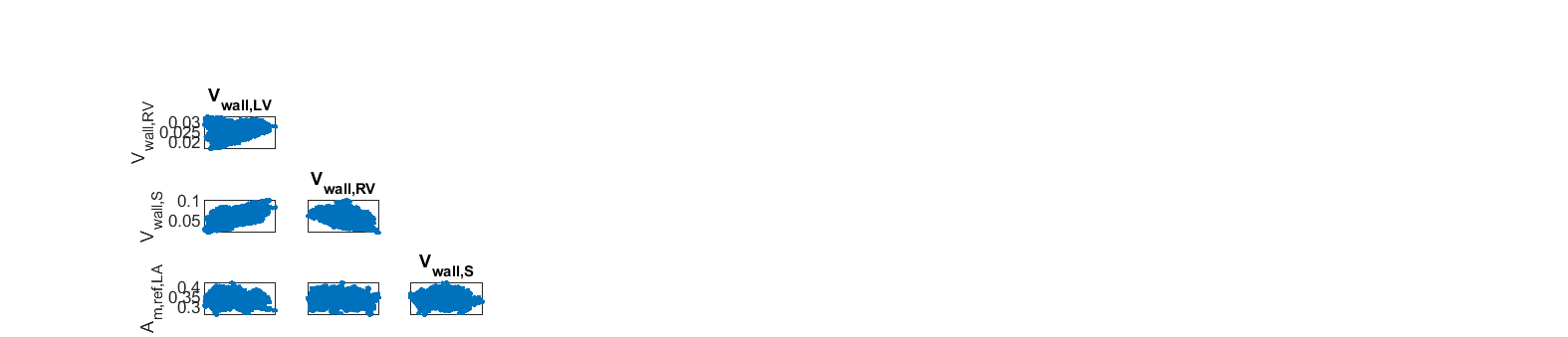


Figure S29: Residual 3 – Iteration 3


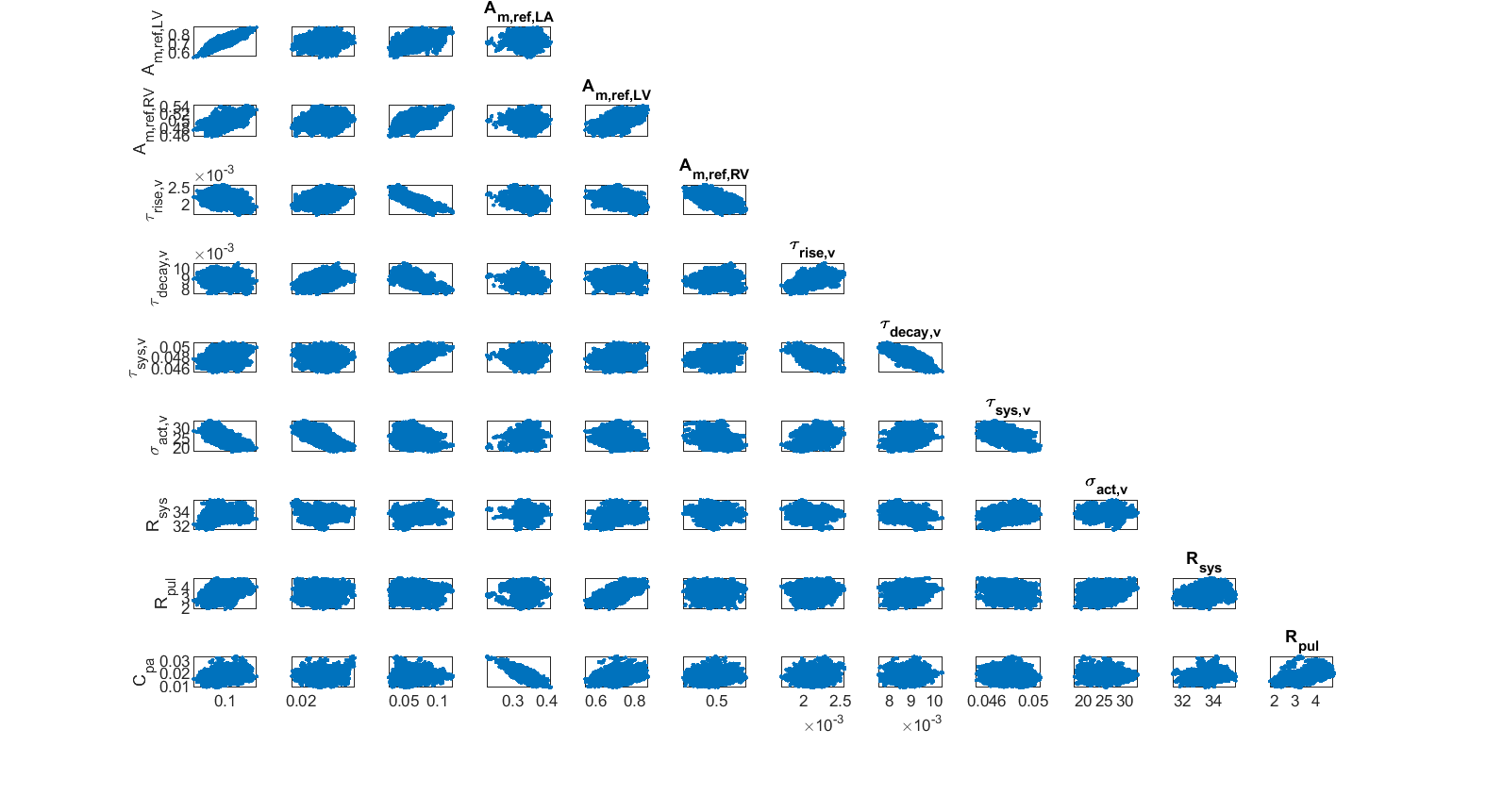

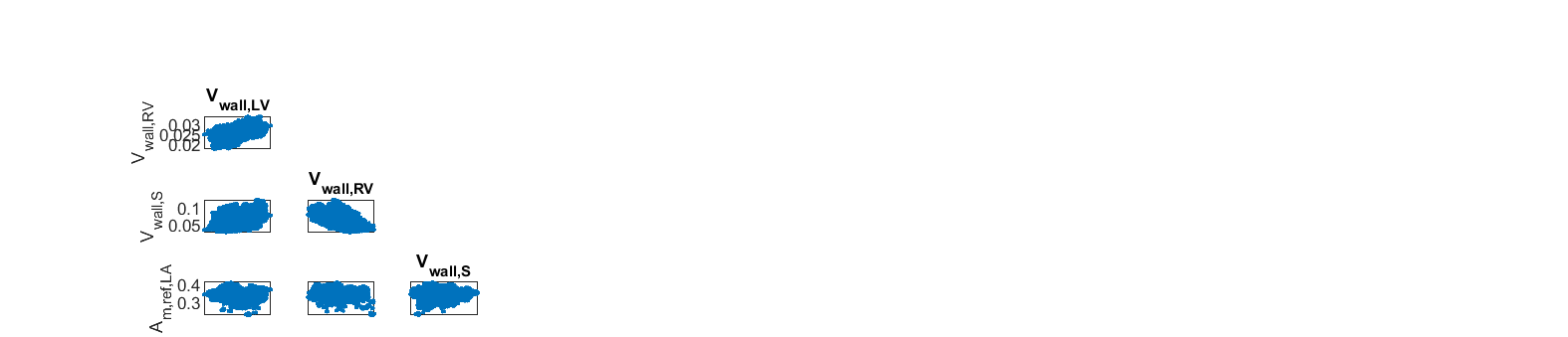


Figure S30: Residual 3 – Iteration 4


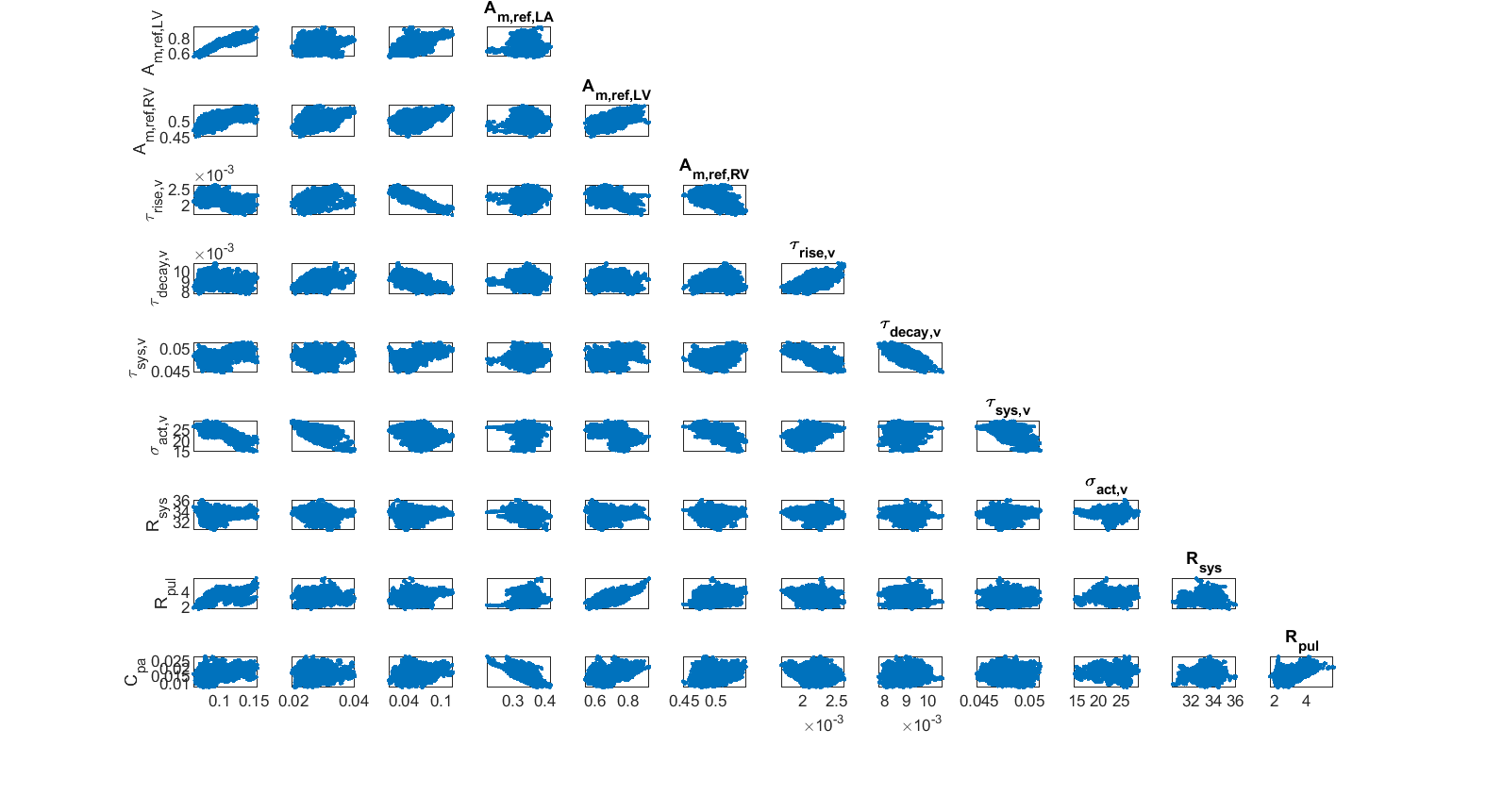

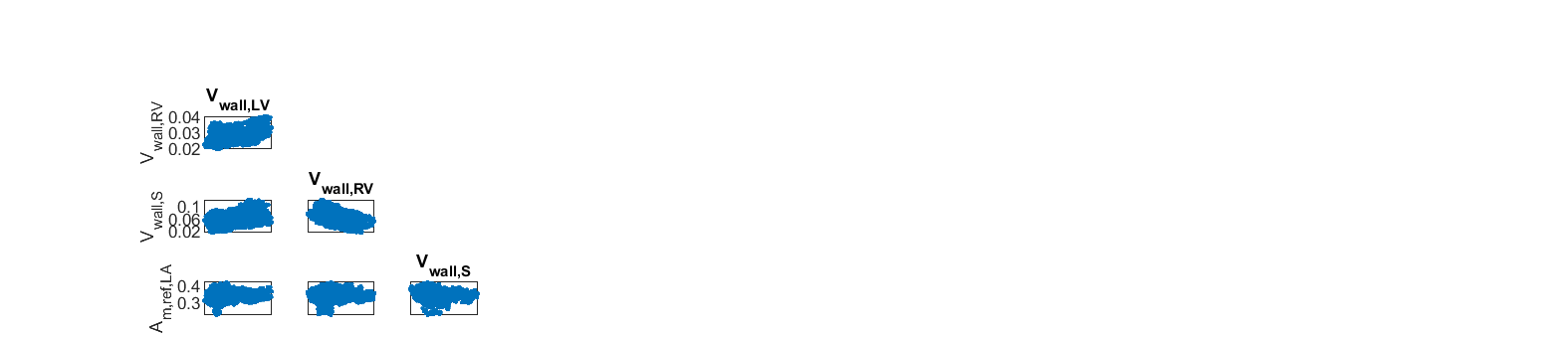


Figure S31: Residual 3 – Iteration 5


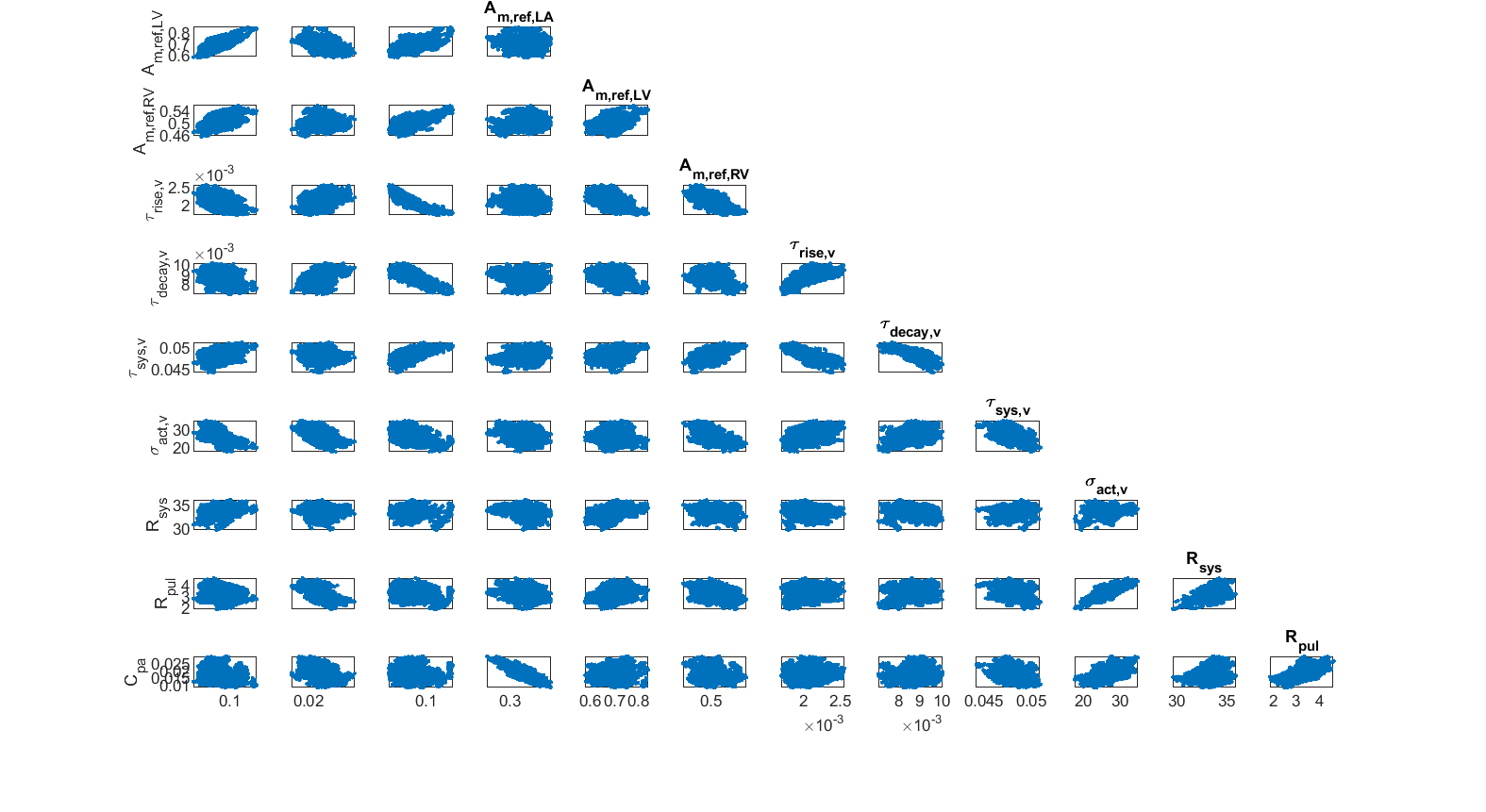

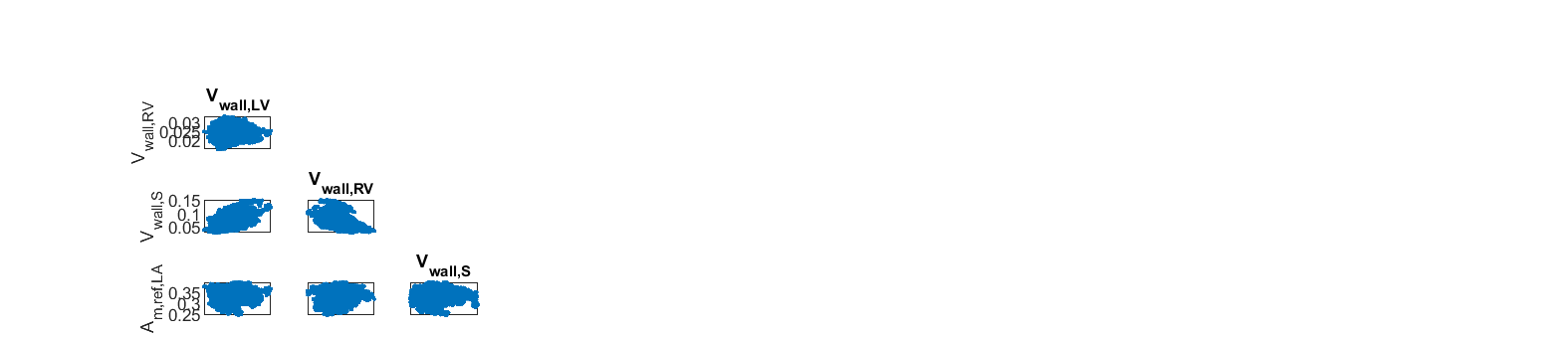


Figure S32: Residual 3 – Iteration 6


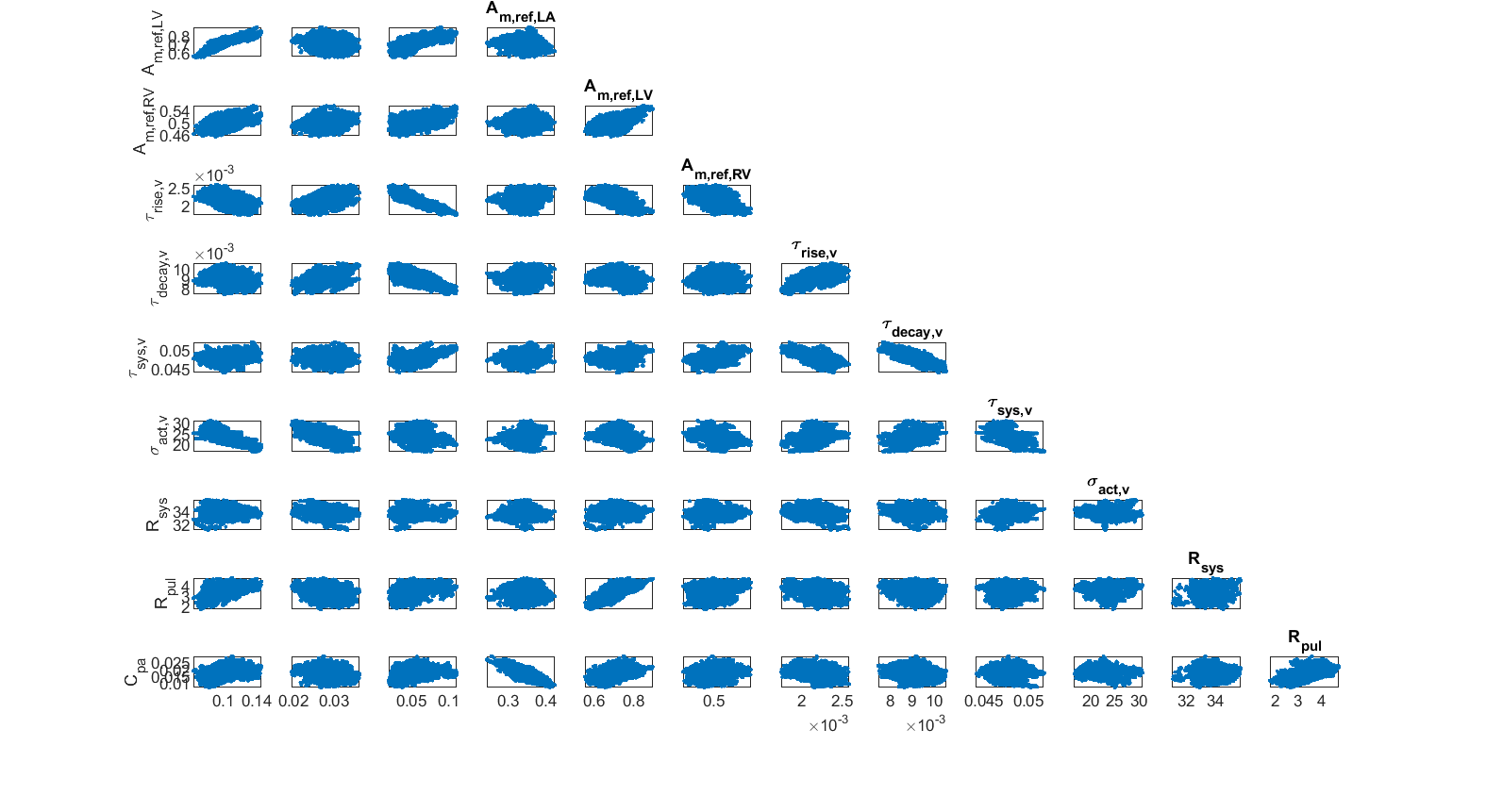

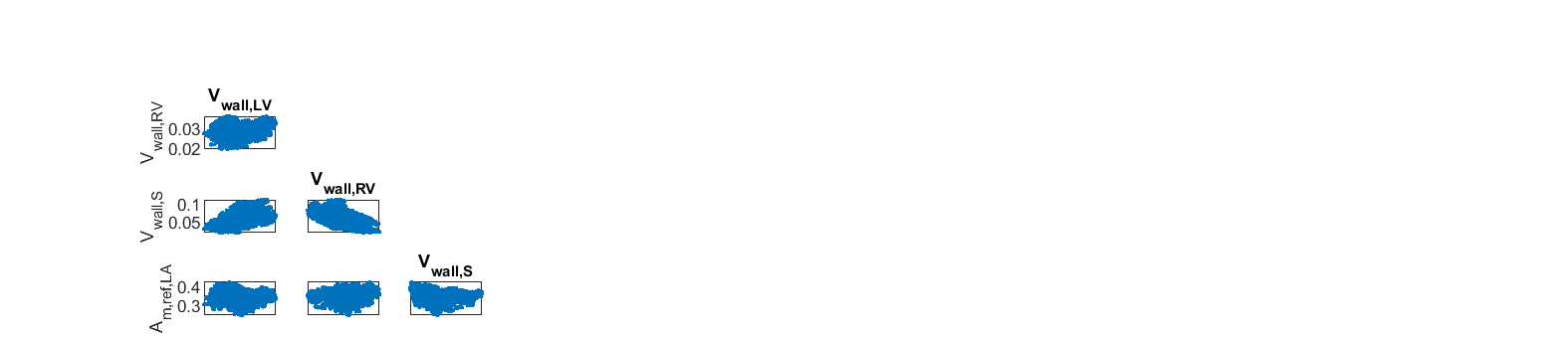


Figure S33: Residual 3 – Iteration 7


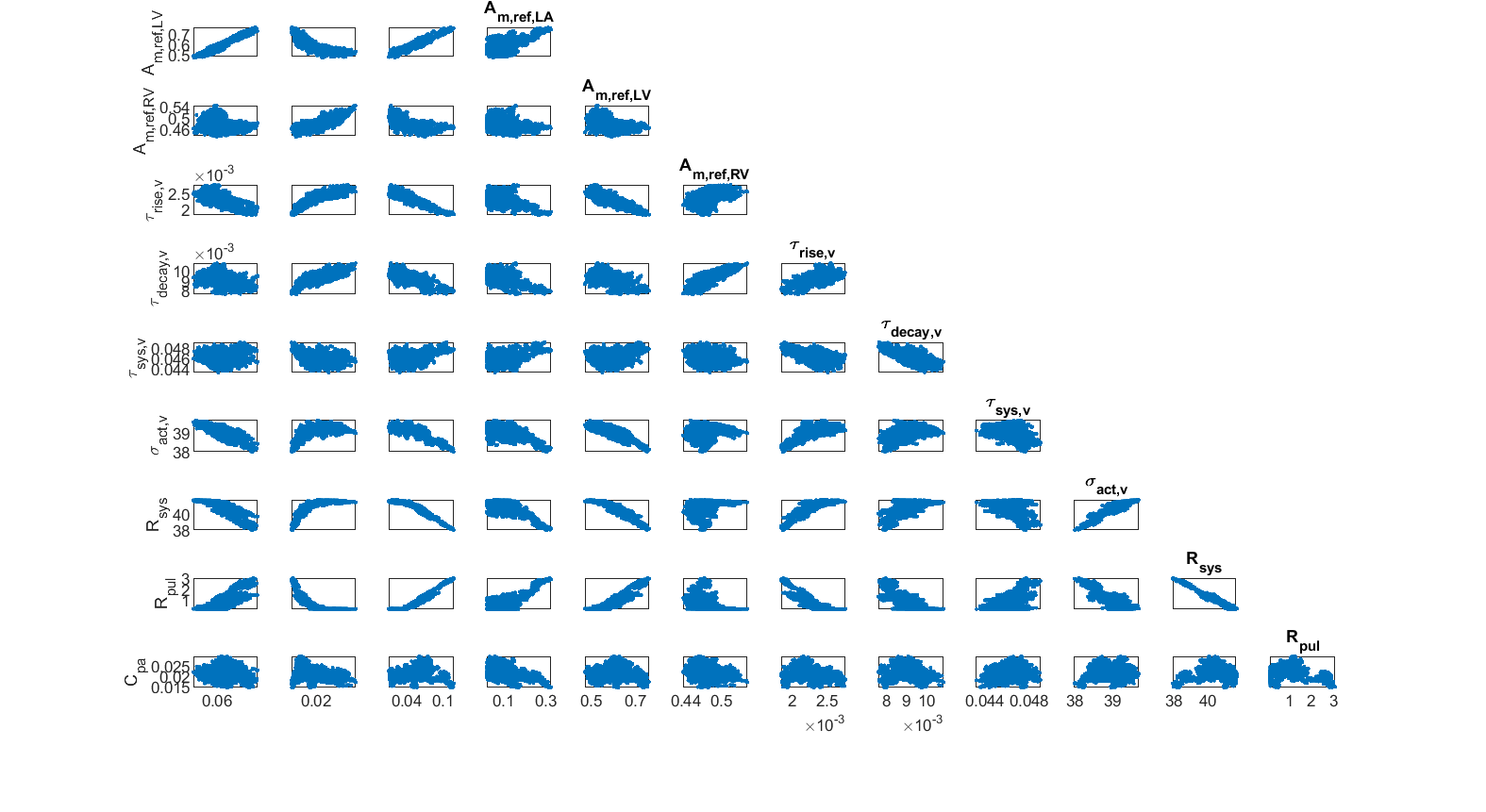

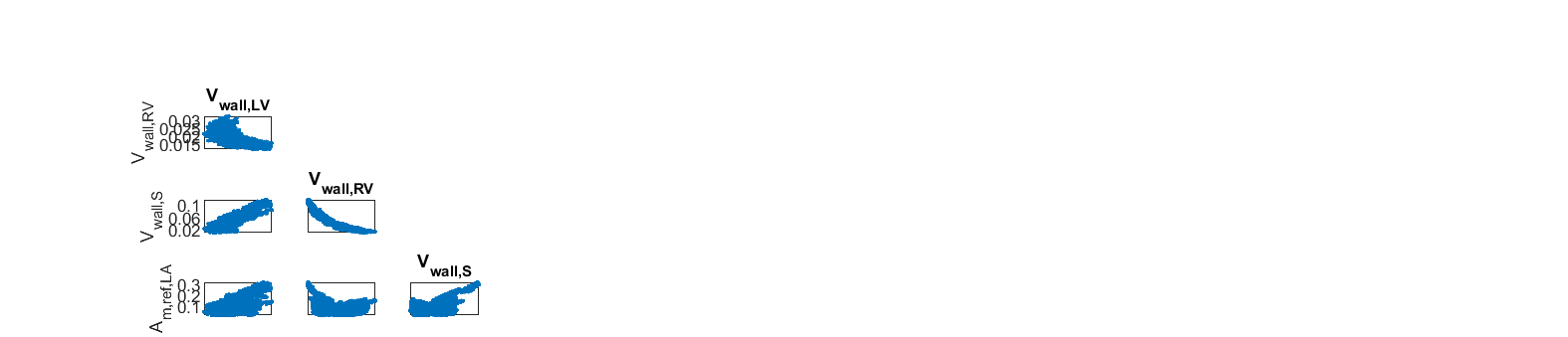


Figure S34: Residual 3 – Iteration 8


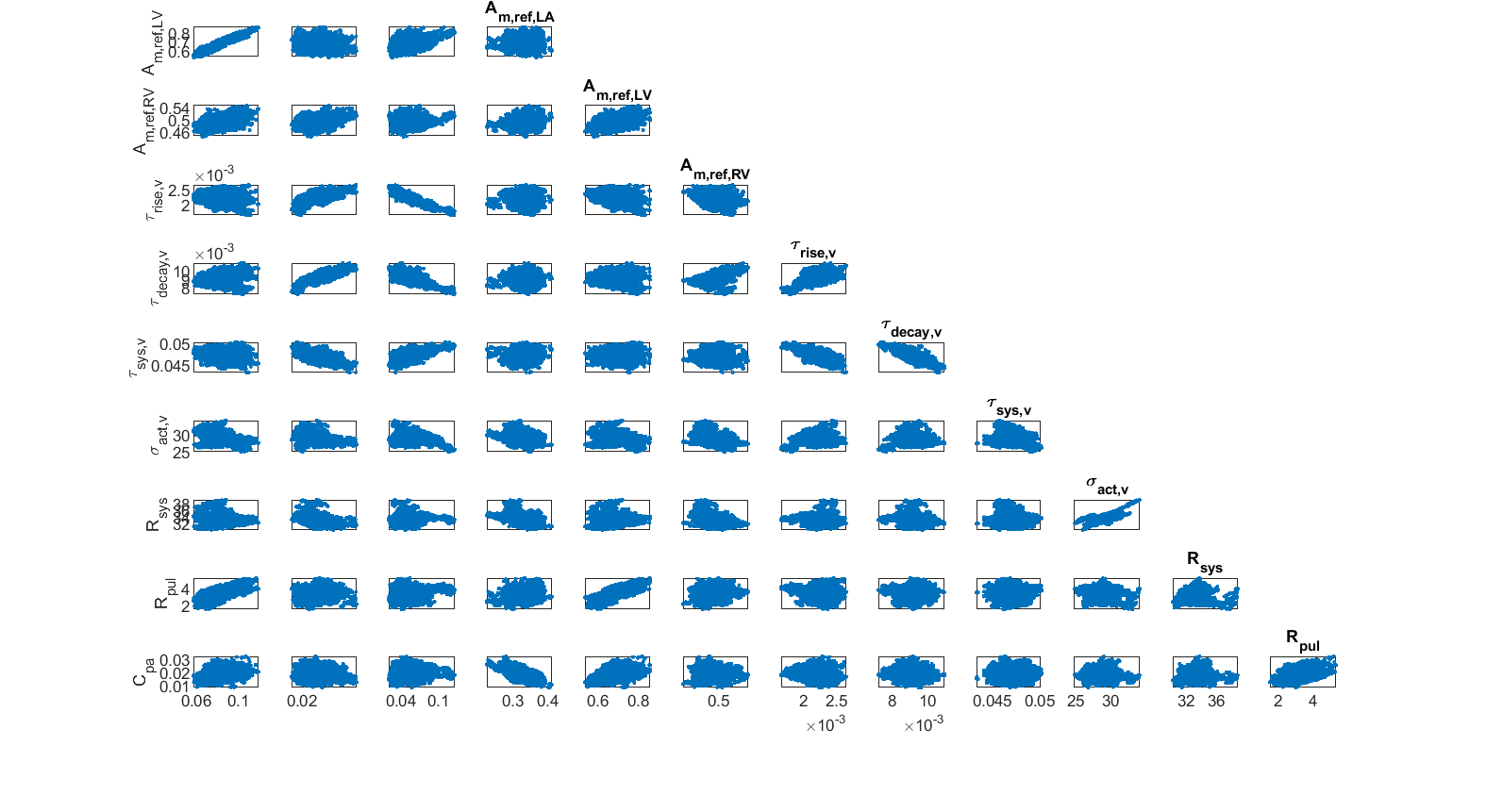

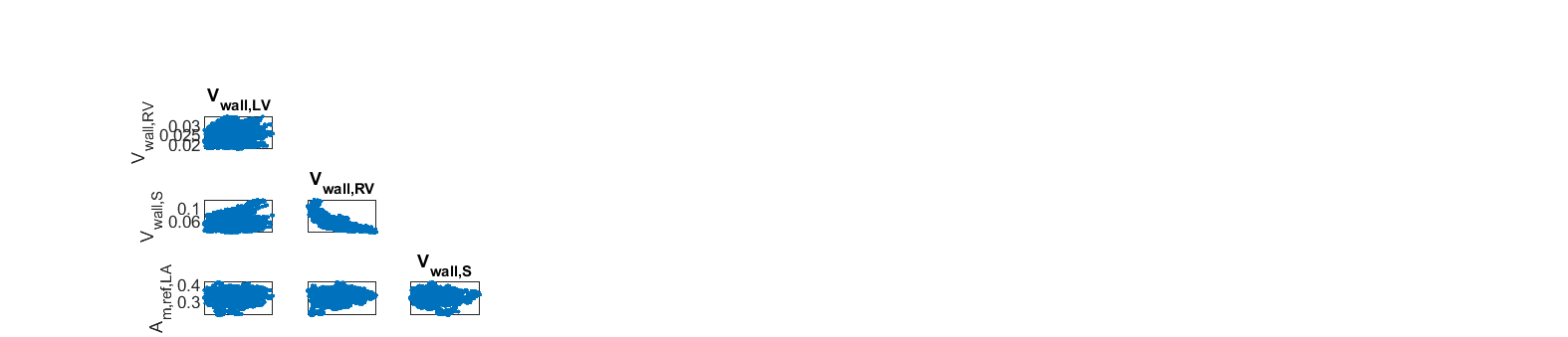


Figure S35: Residual 3 – Iteration 9


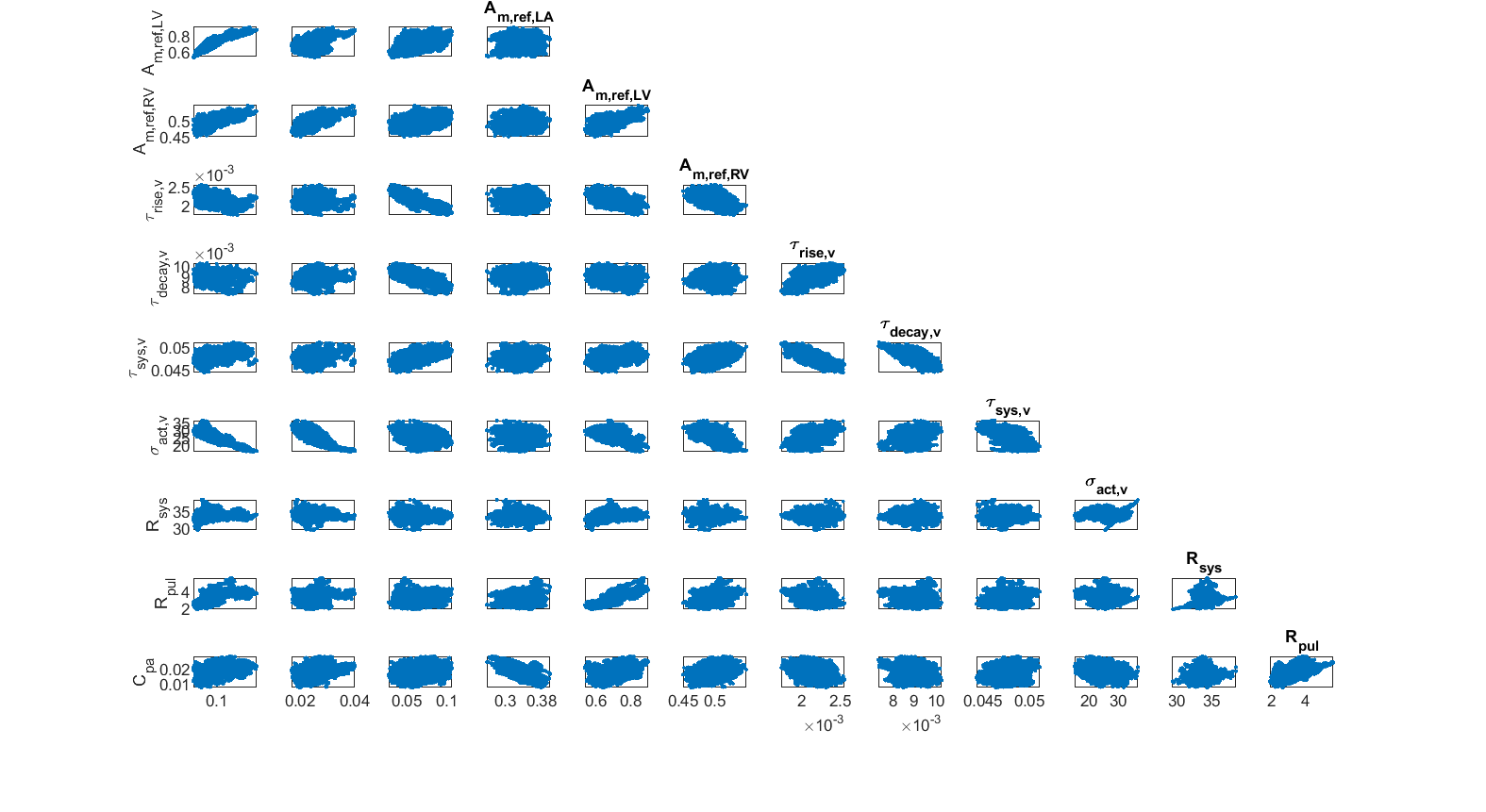

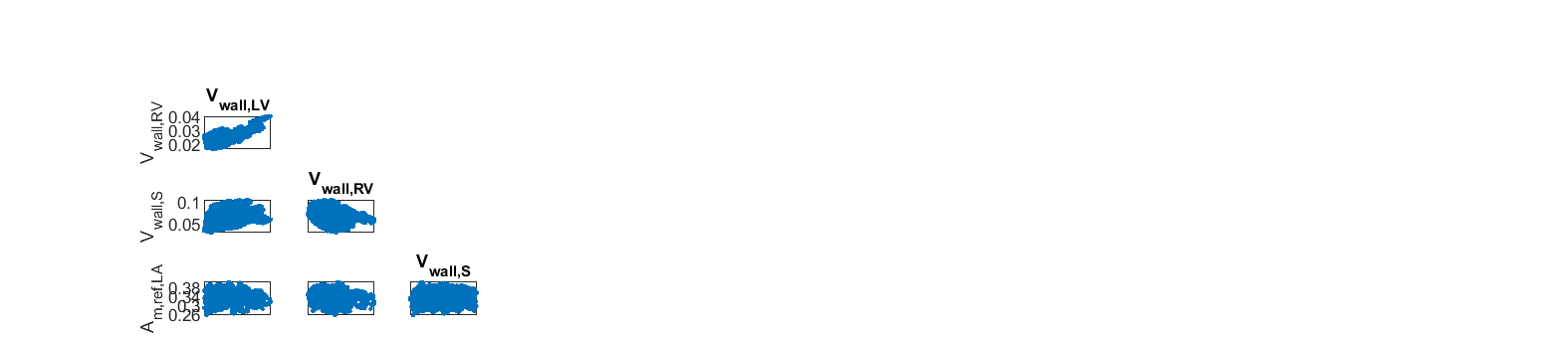


Figure S36: Residual 3 – Iteration 10


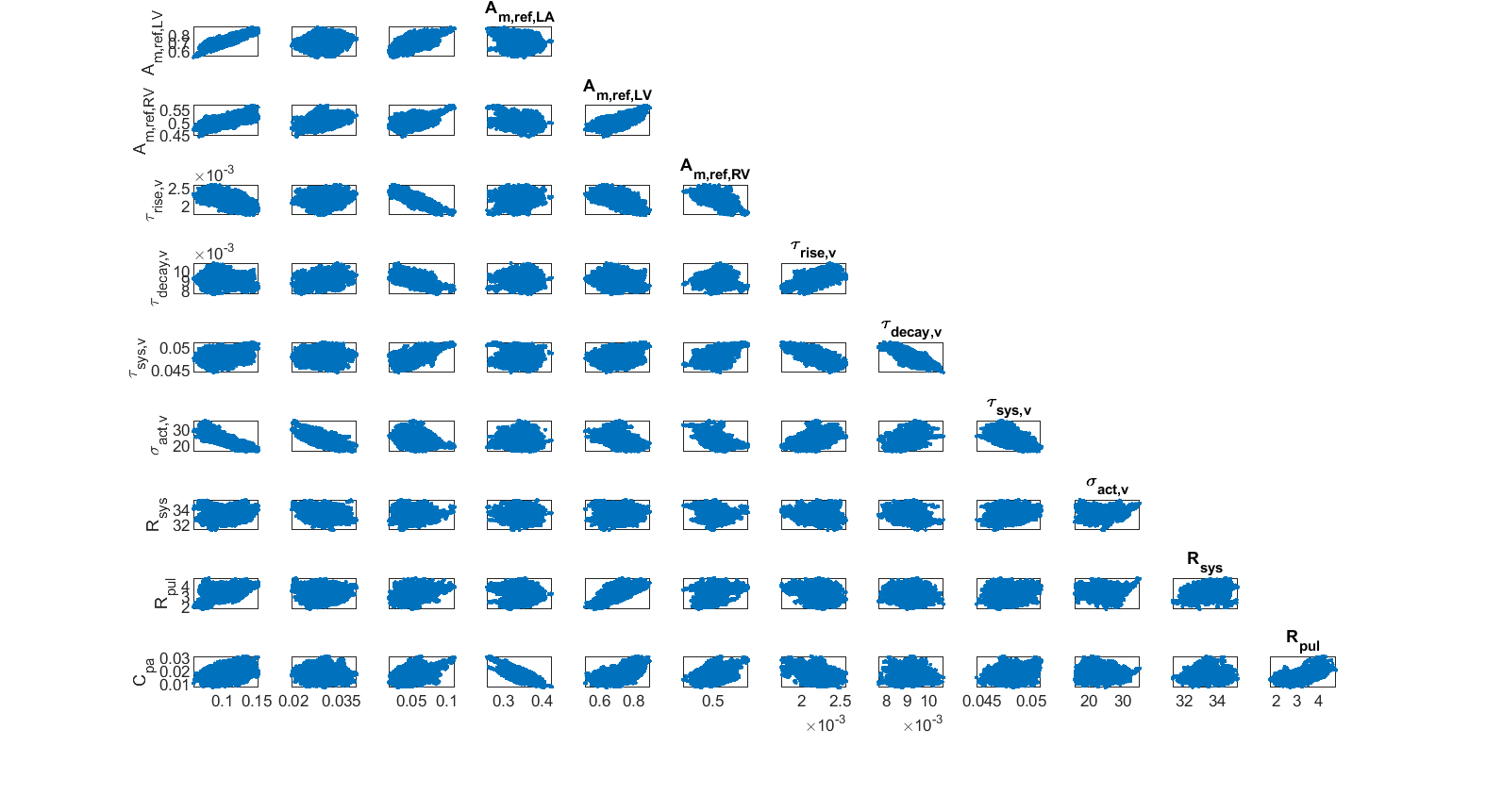

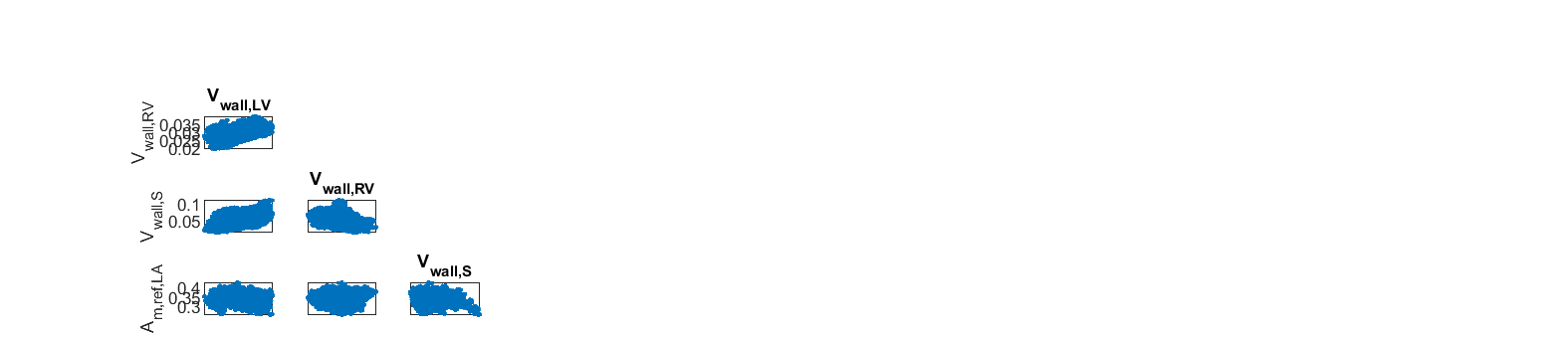


Figure S37: Residual 3 – Iteration 11


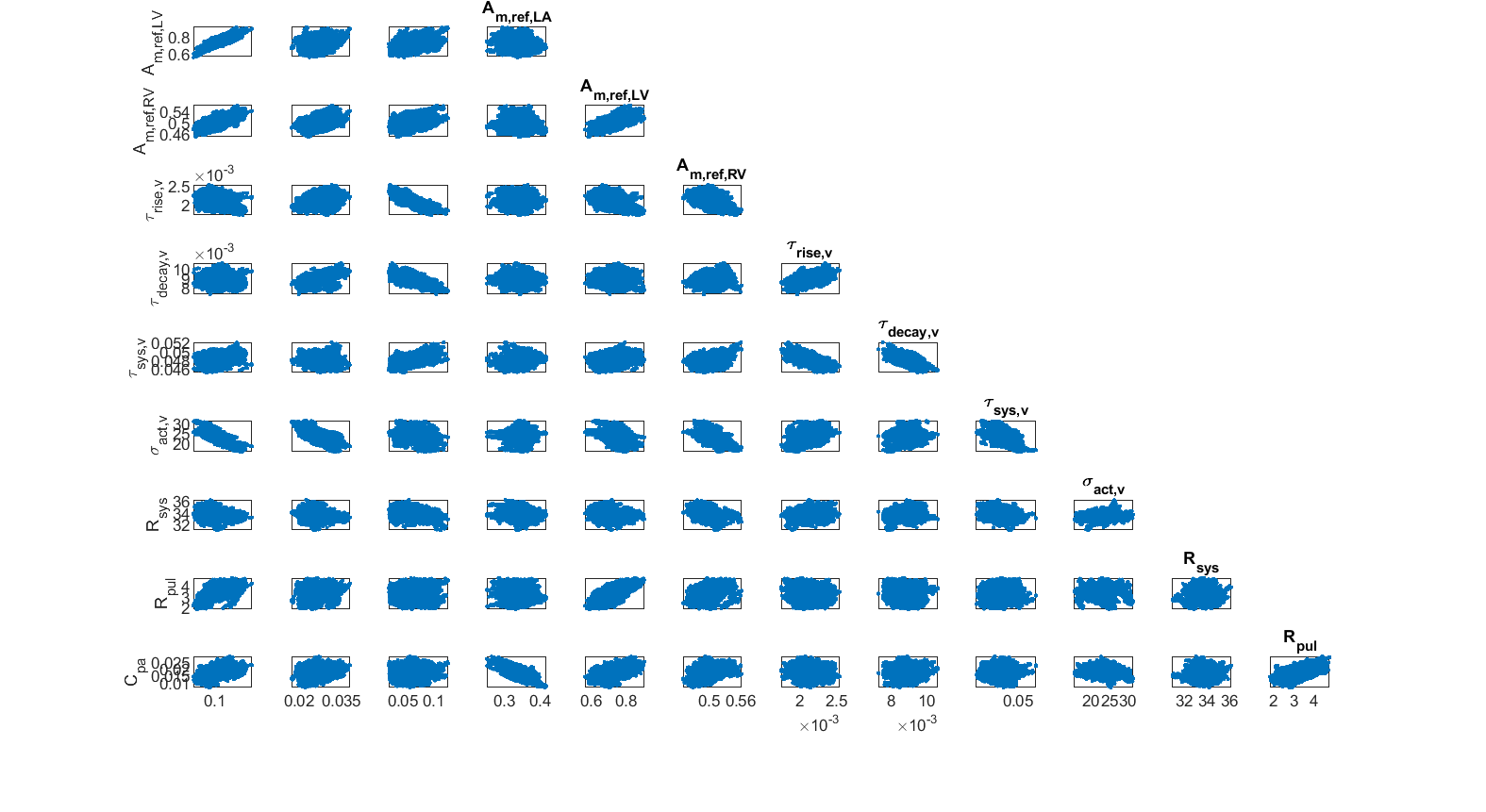

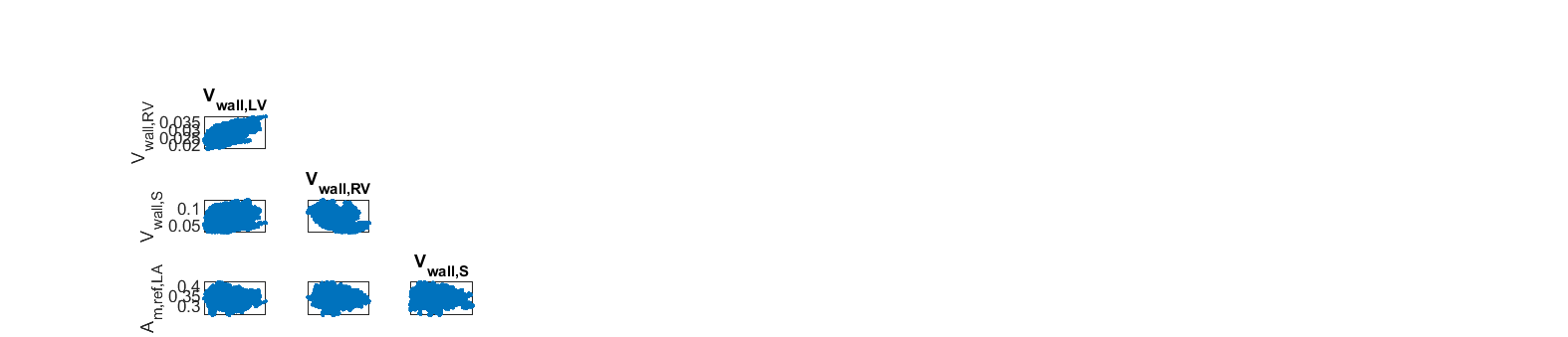


Figure S38: Residual 3 – Iteration 12


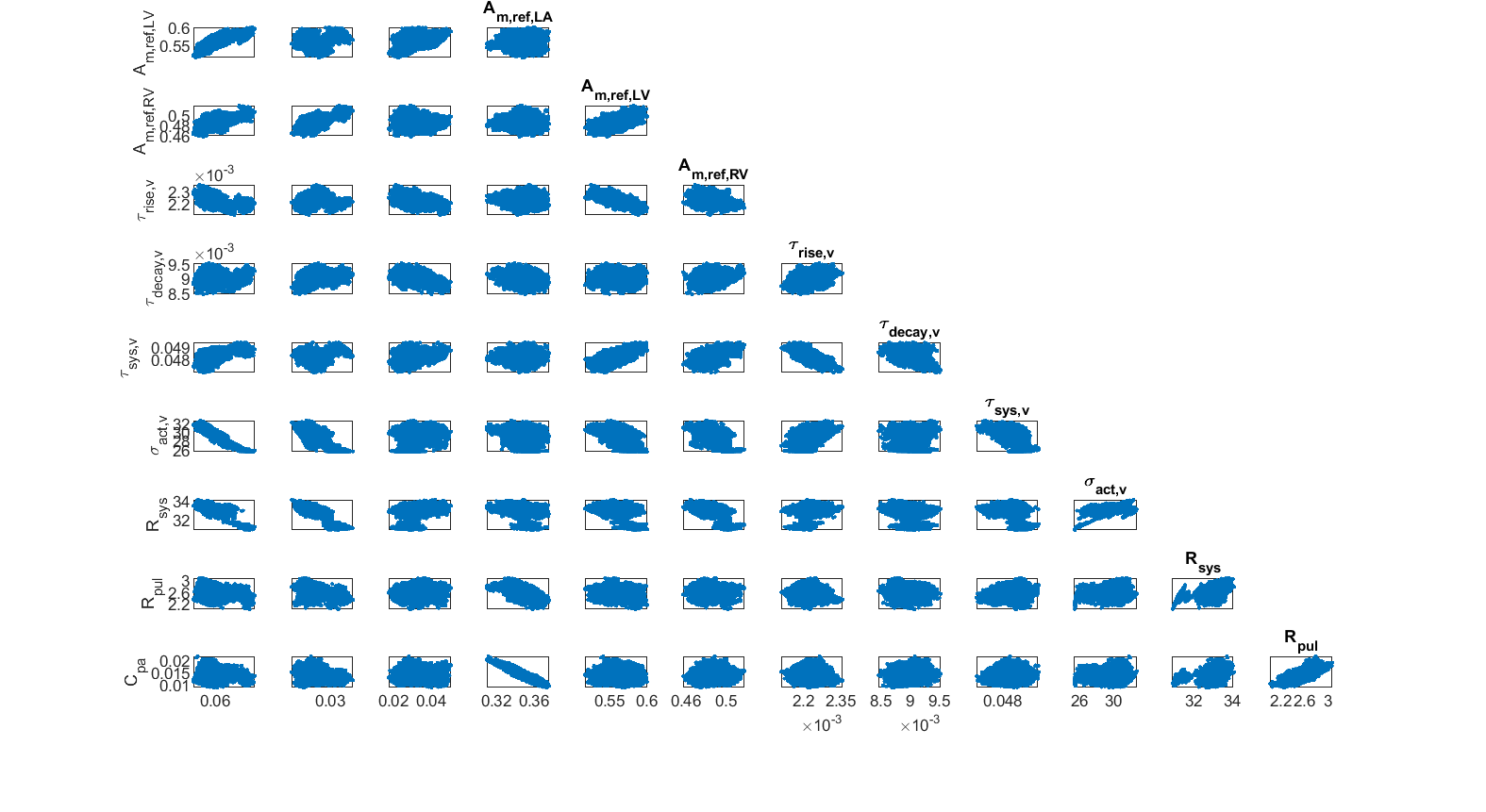

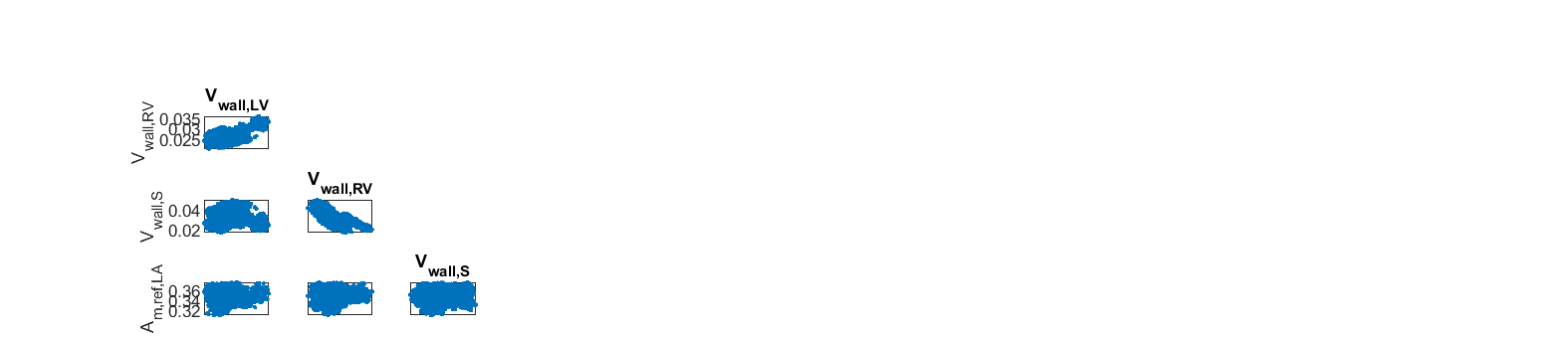


Figure S39: Residual 4 – Iteration 1


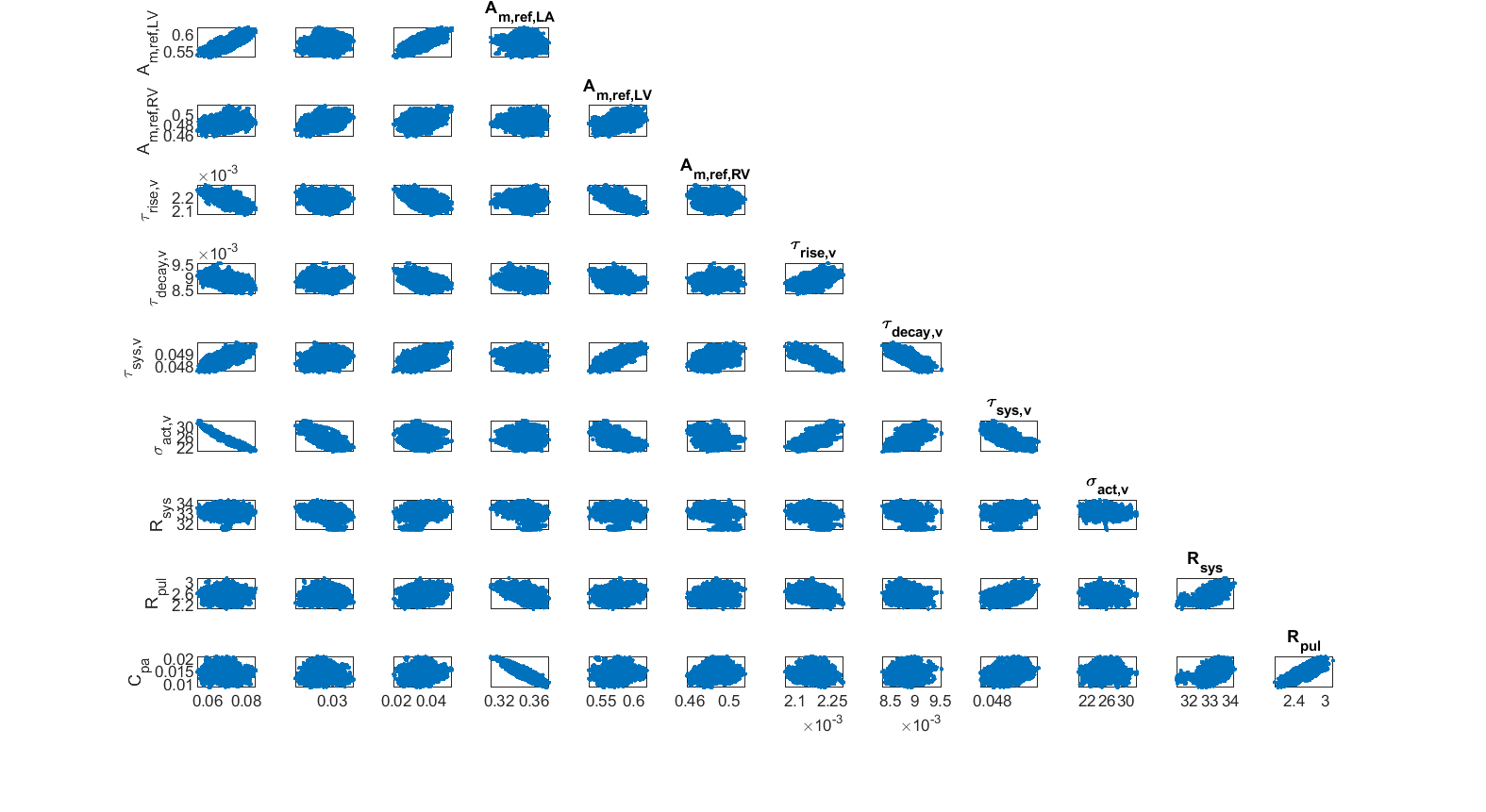

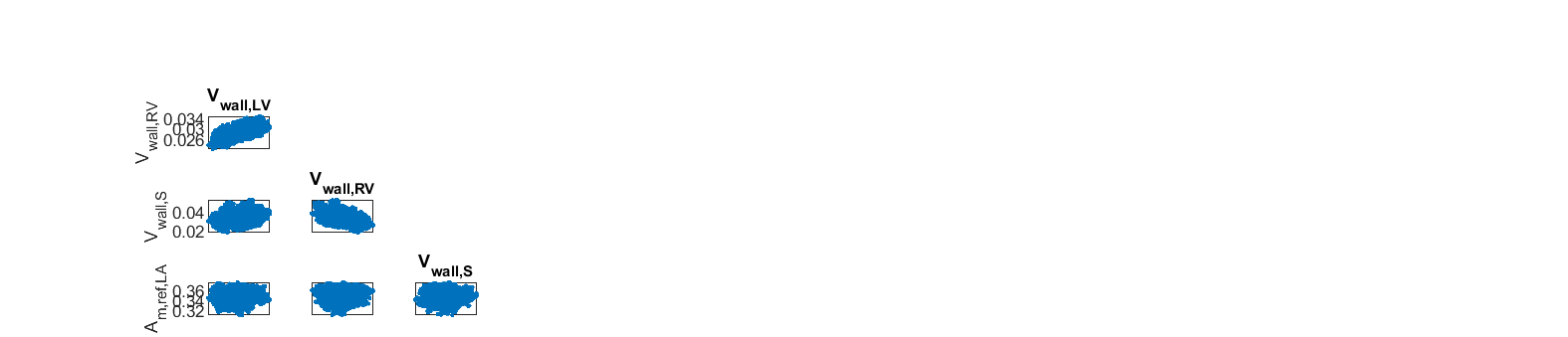


Figure S40: Residual 4 – Iteration 2


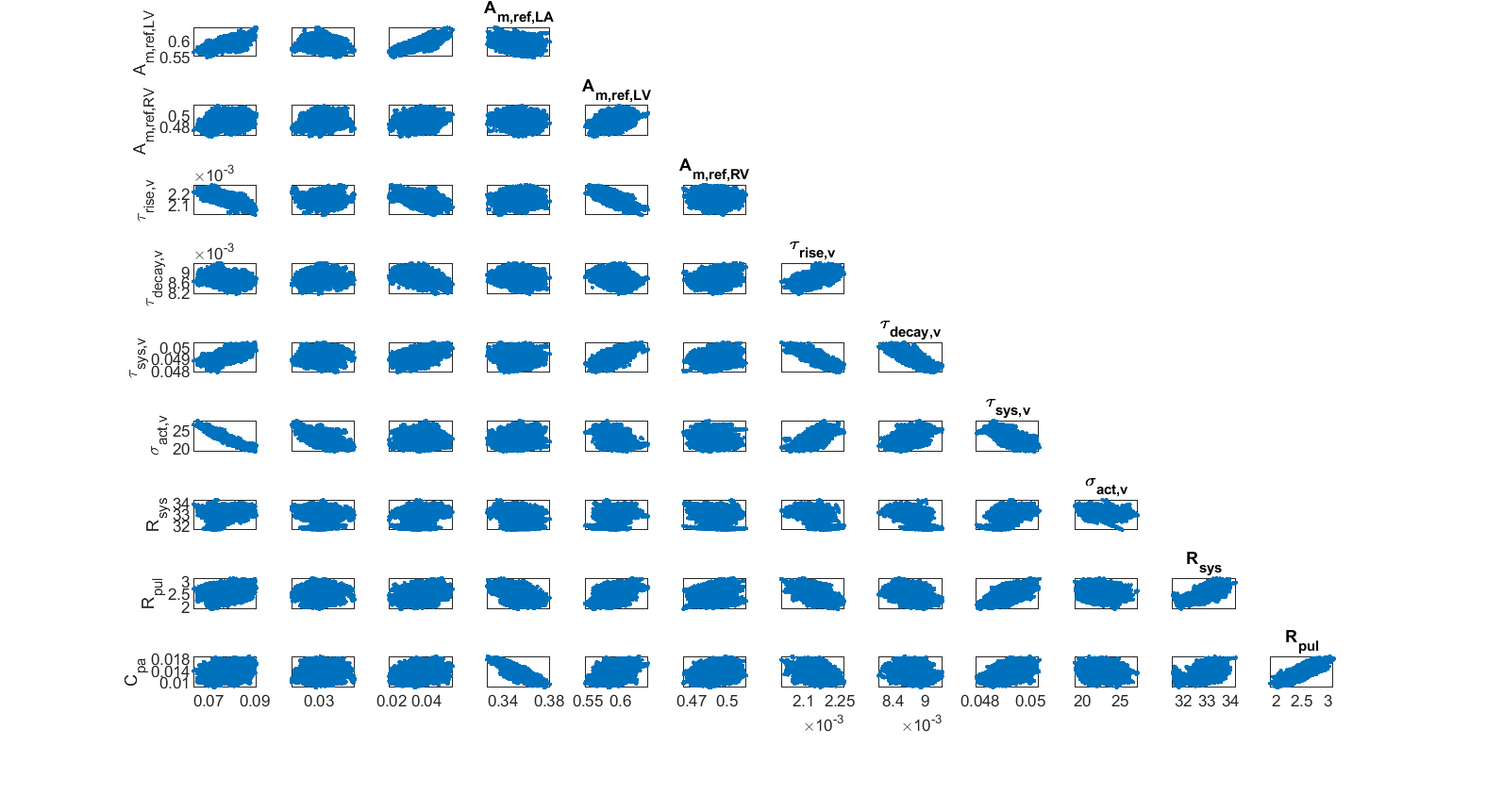

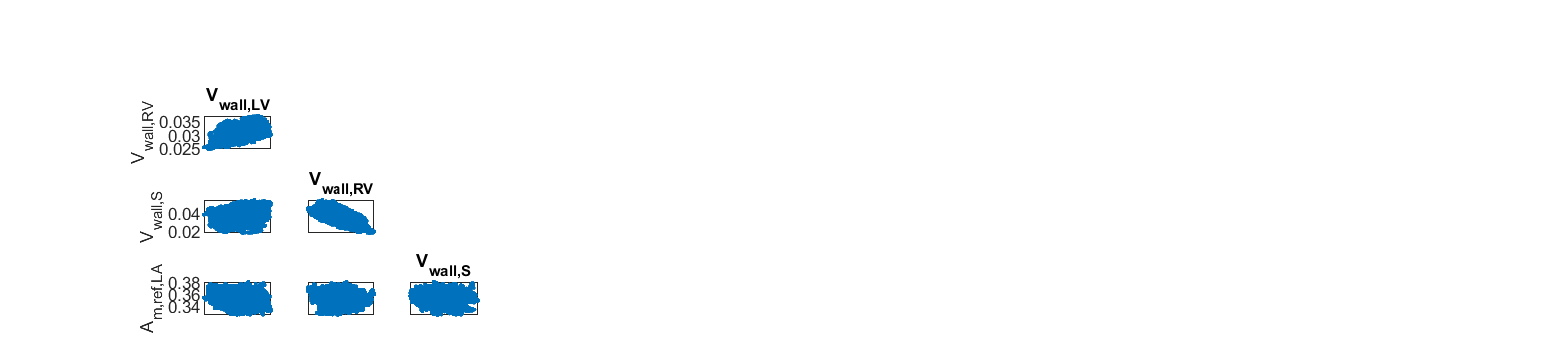


Figure S41: Residual 4 – Iteration 3


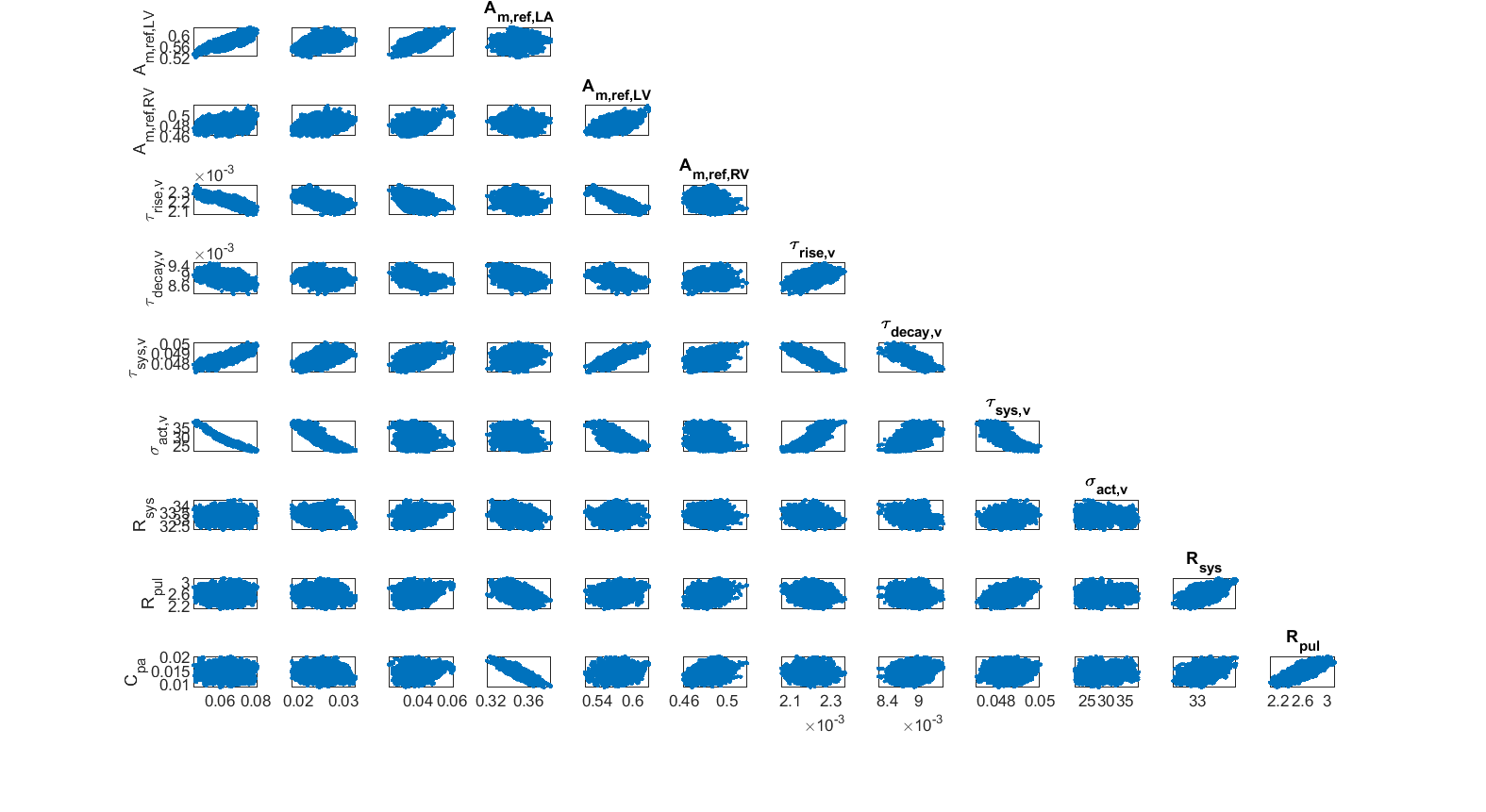

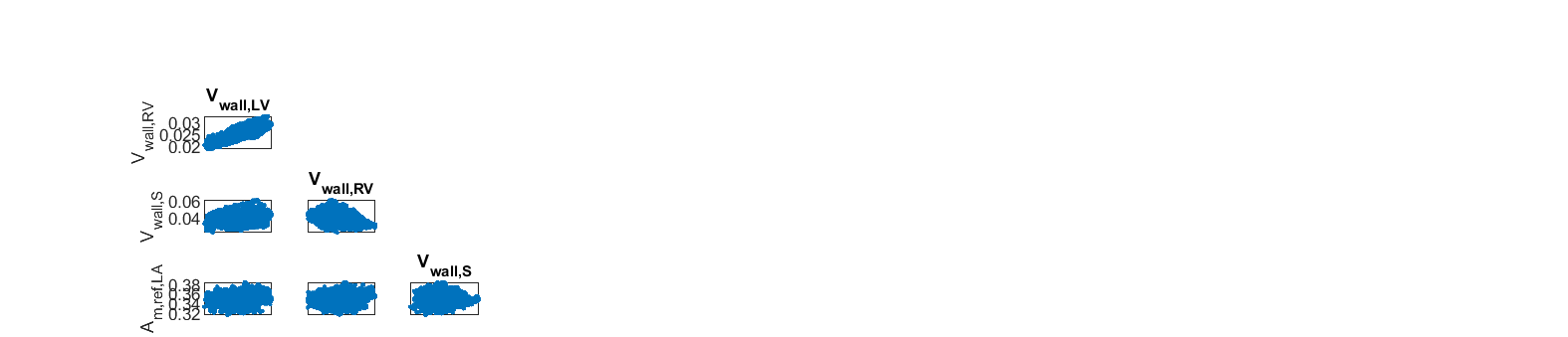


Figure S42: Residual 4 – Iteration 4


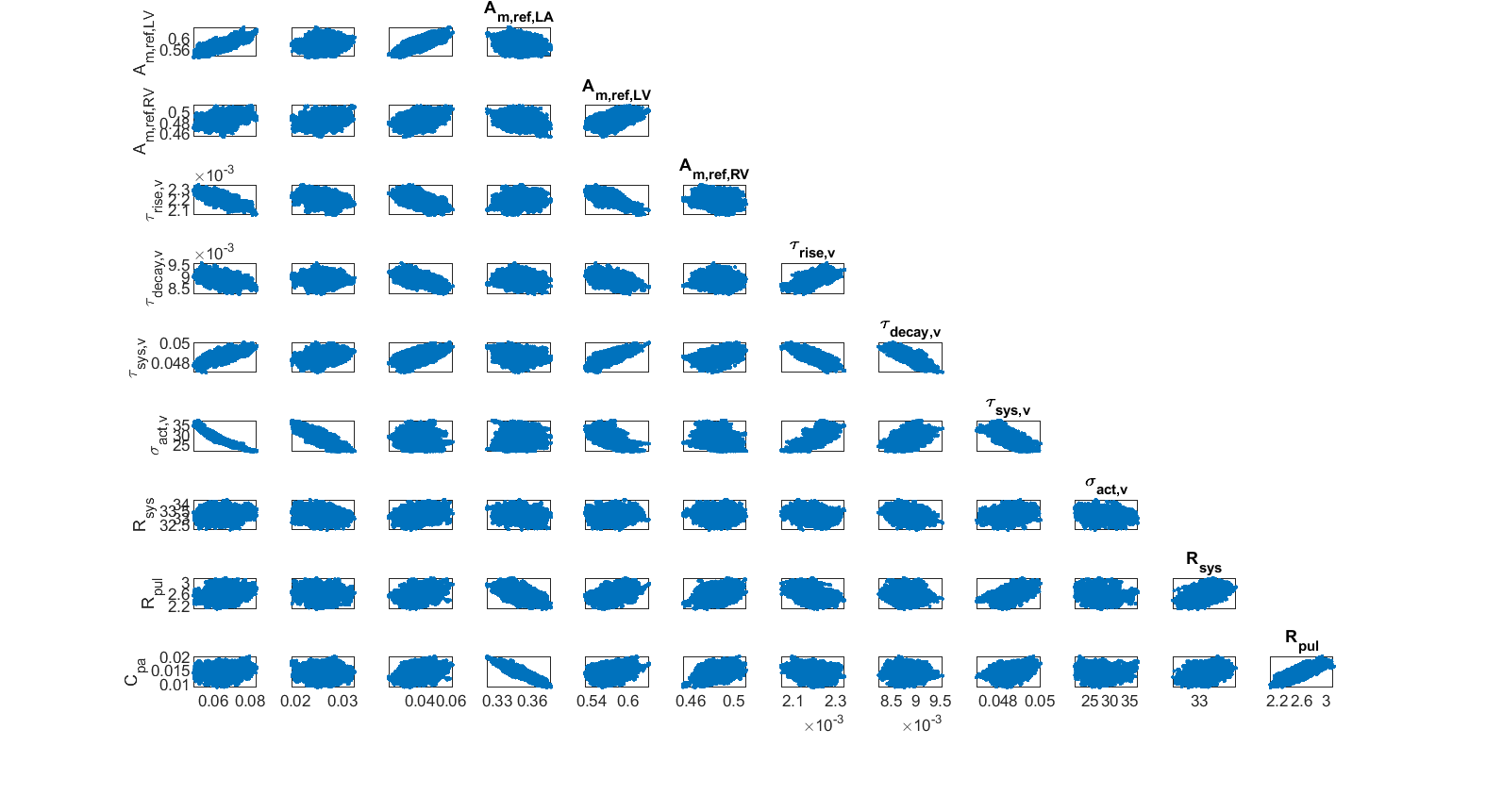

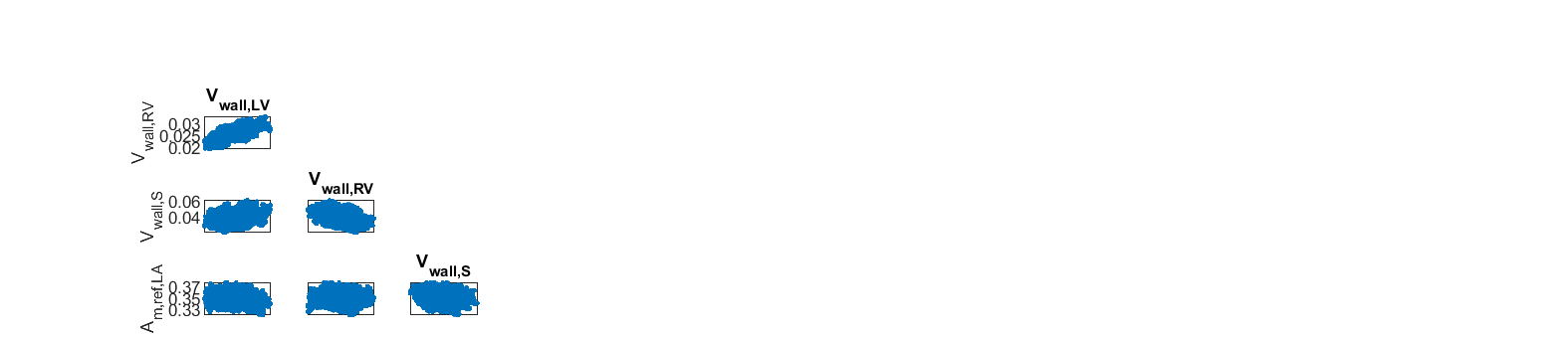


Figure S43: Residual 4 – Iteration 5


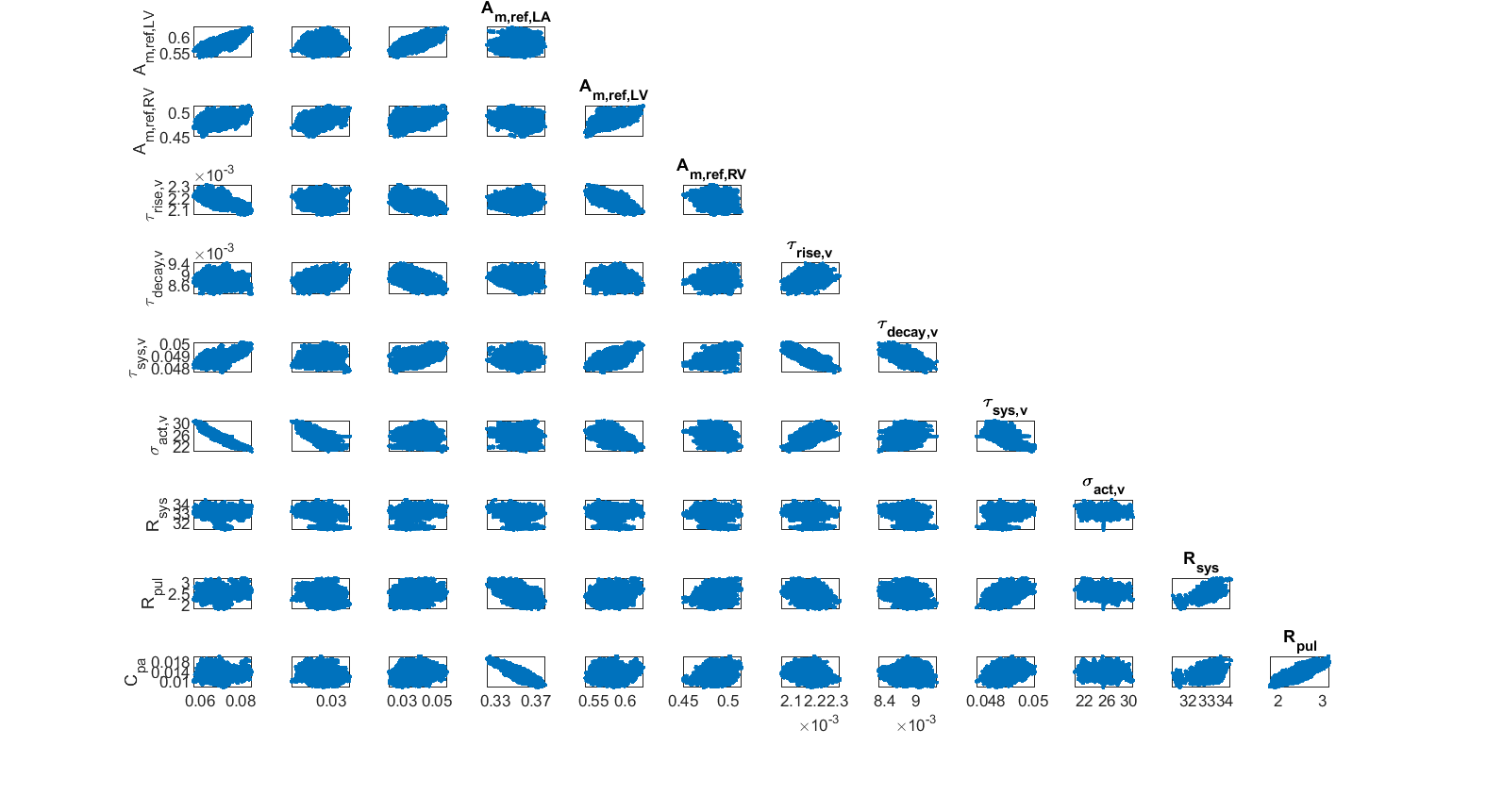

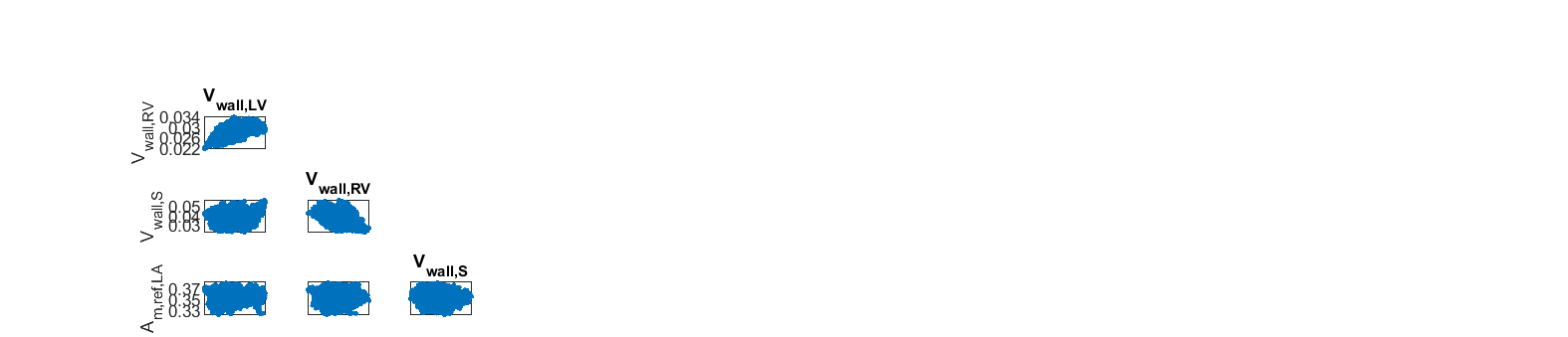


Figure S44: Residual 4 – Iteration 6


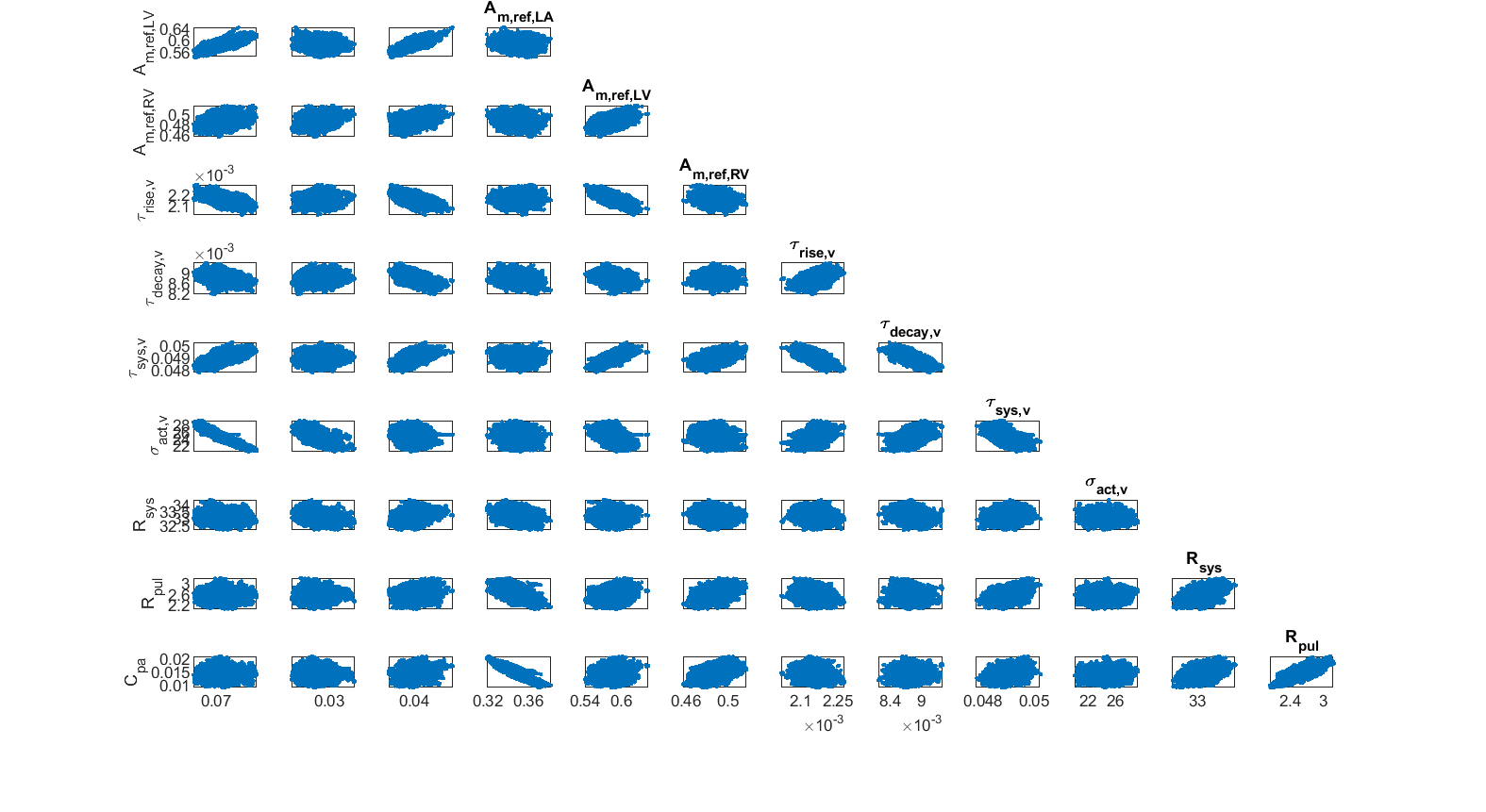

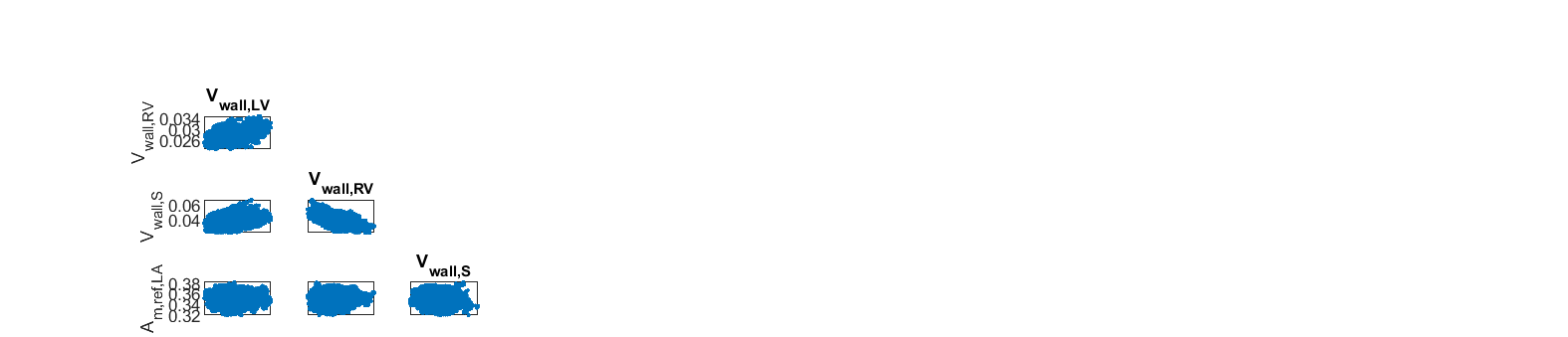


Figure S45: Residual 4 – Iteration 7


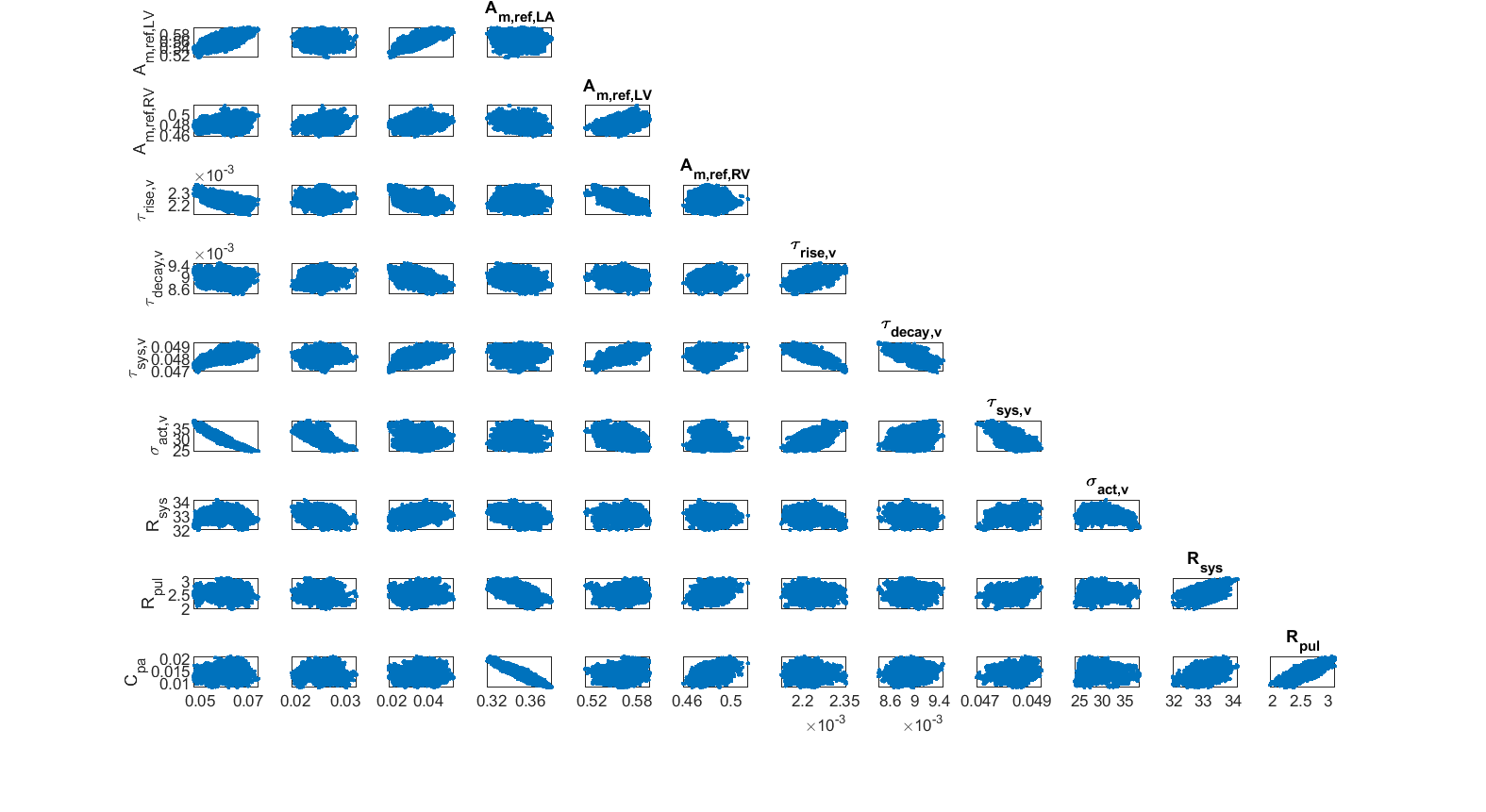

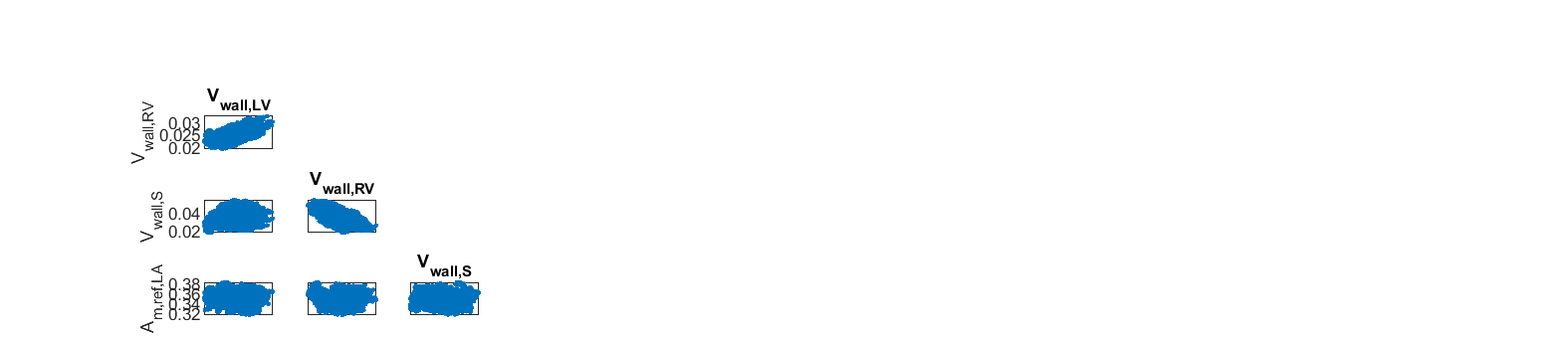


Figure S46: Residual 4 – Iteration 8


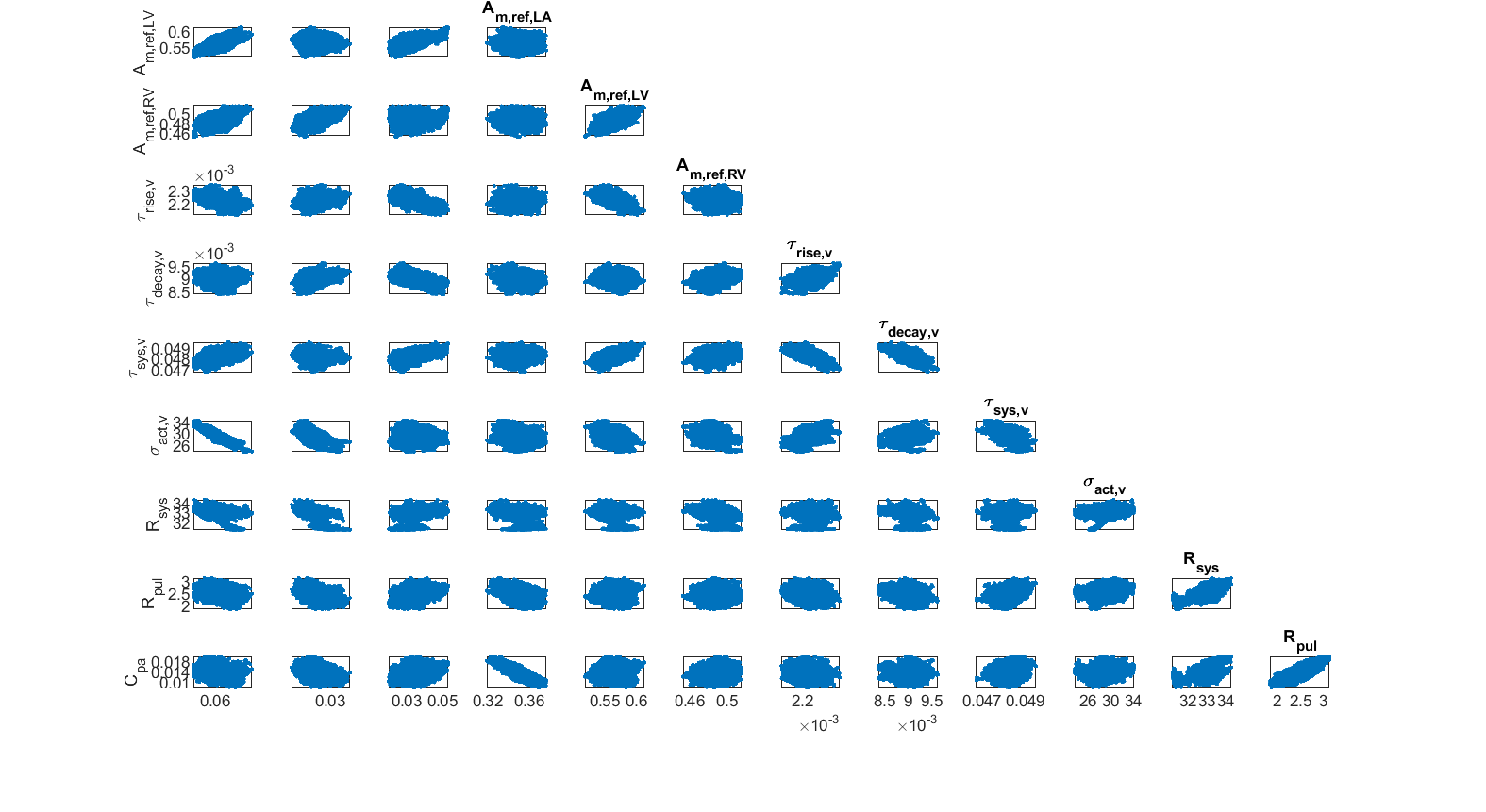

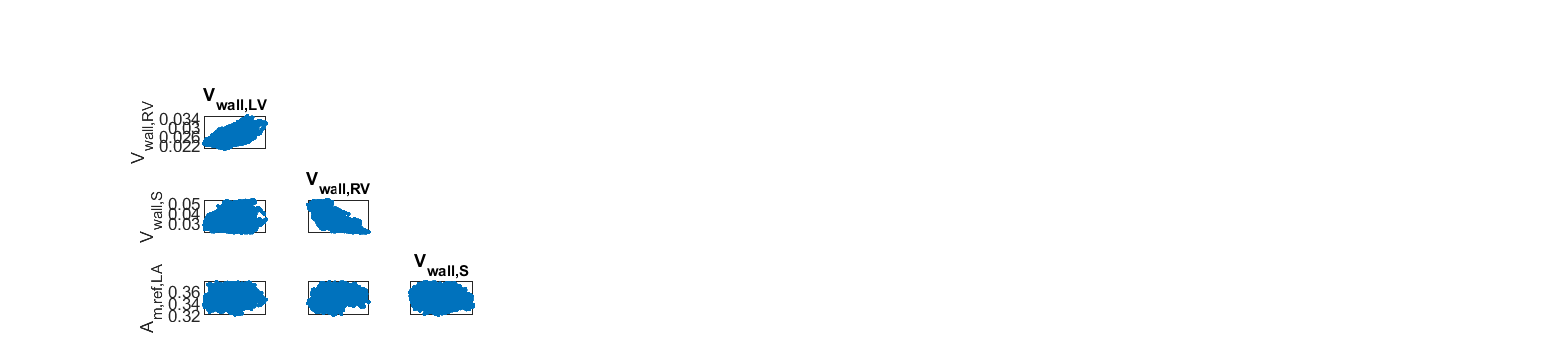


Figure S47: Residual 4 – Iteration 9


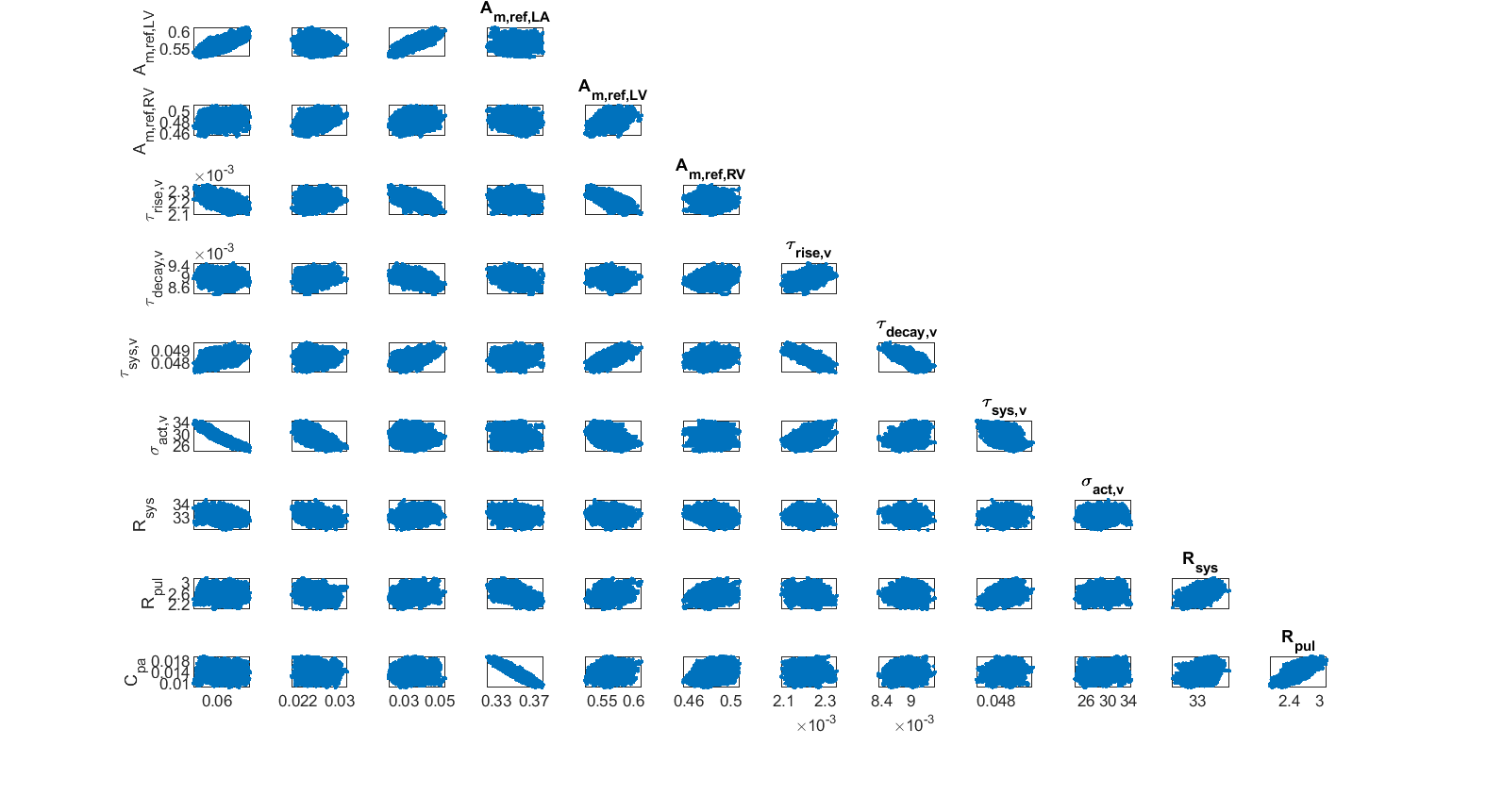

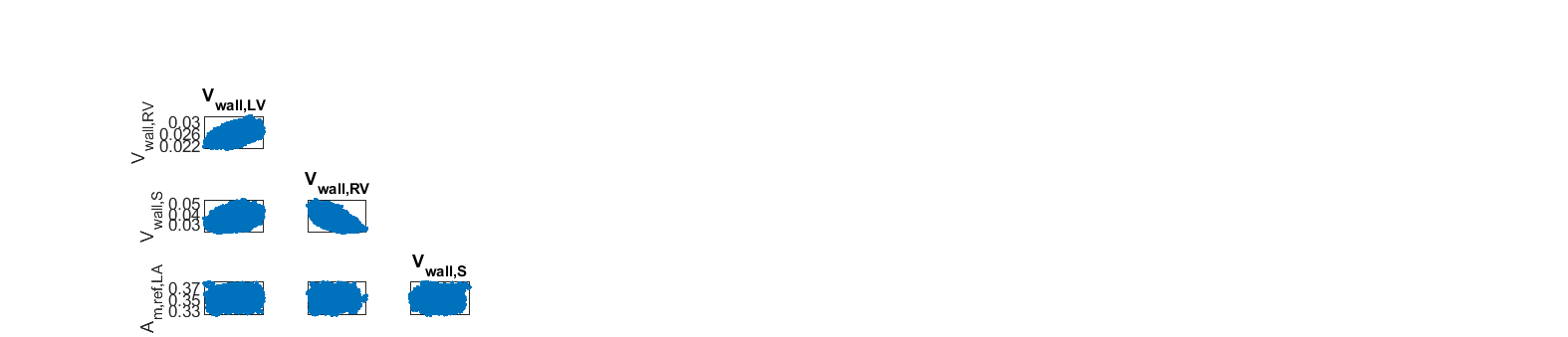


Figure S48: Residual 4 – Iteration 10


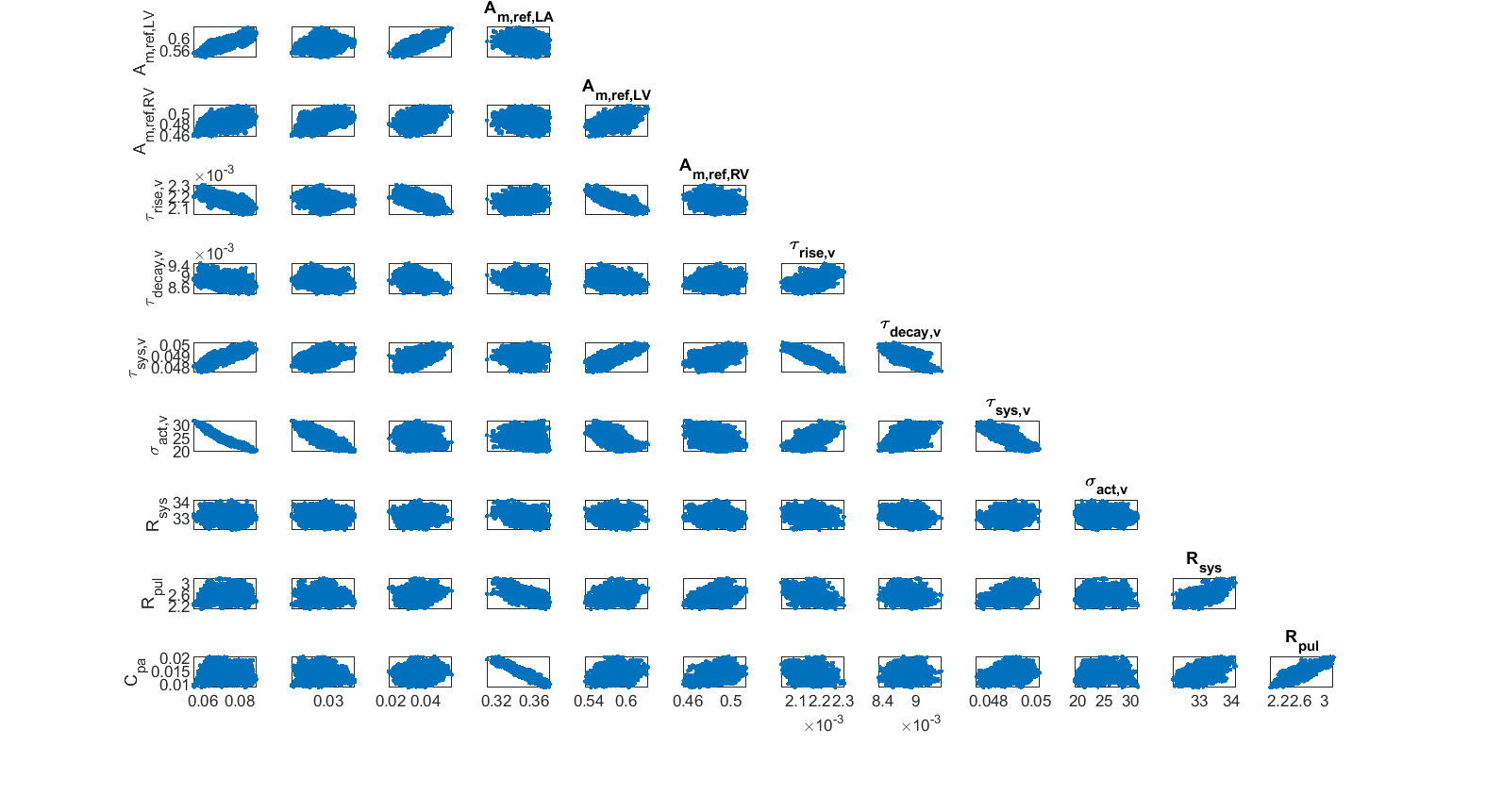

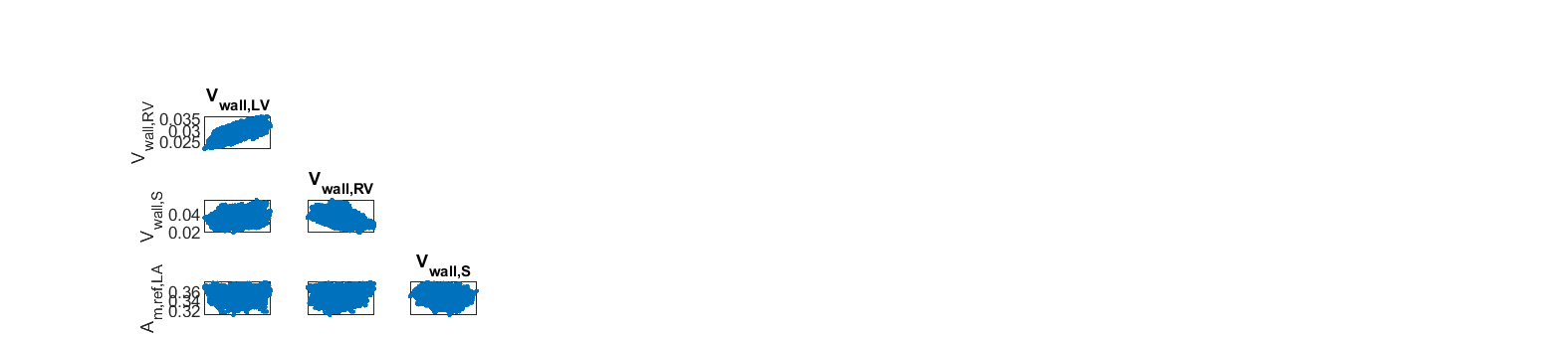


Figure S49: Residual 4 – Iteration 11

Figure S50: Residual 4 – Iteration 12


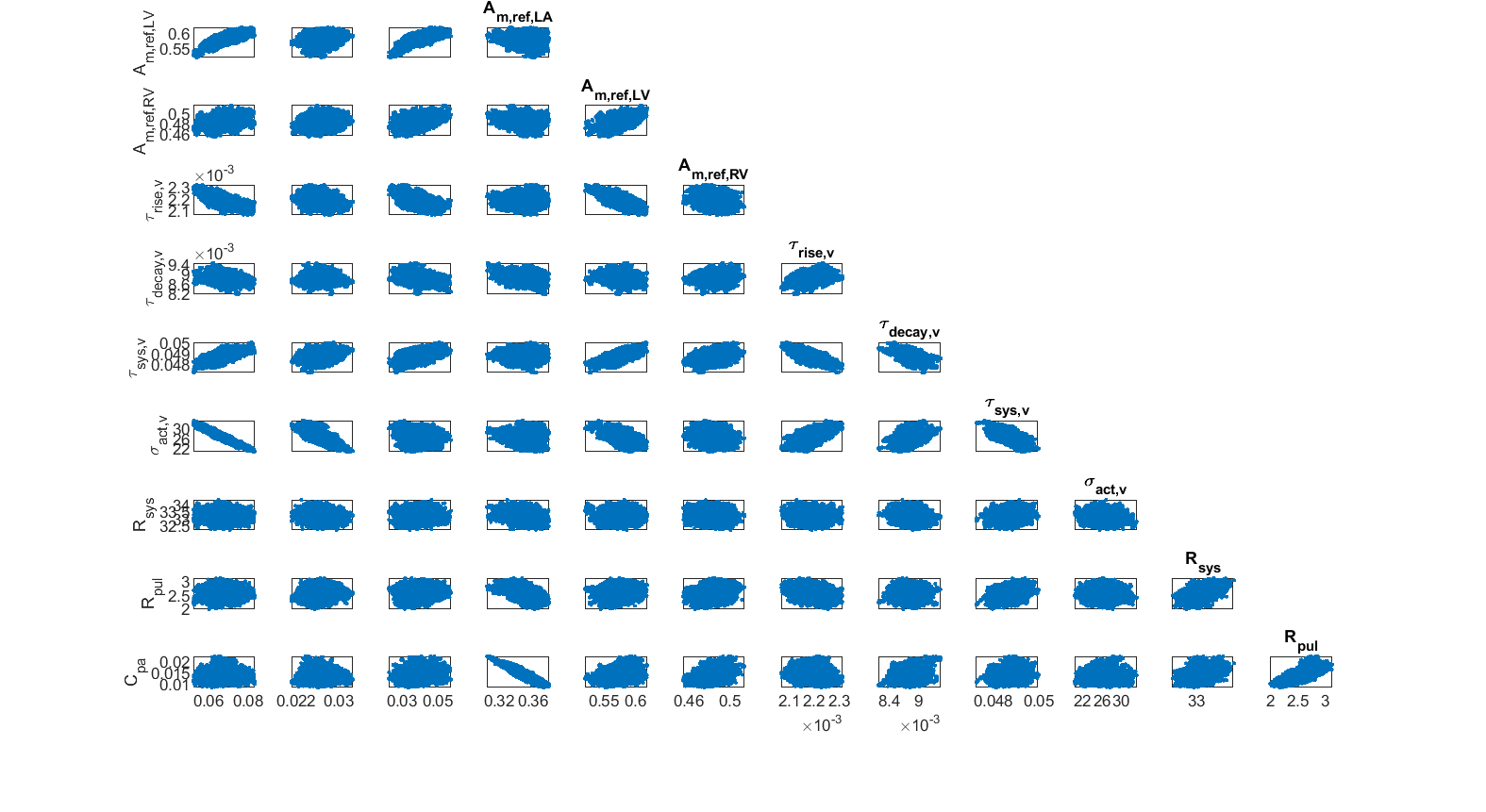

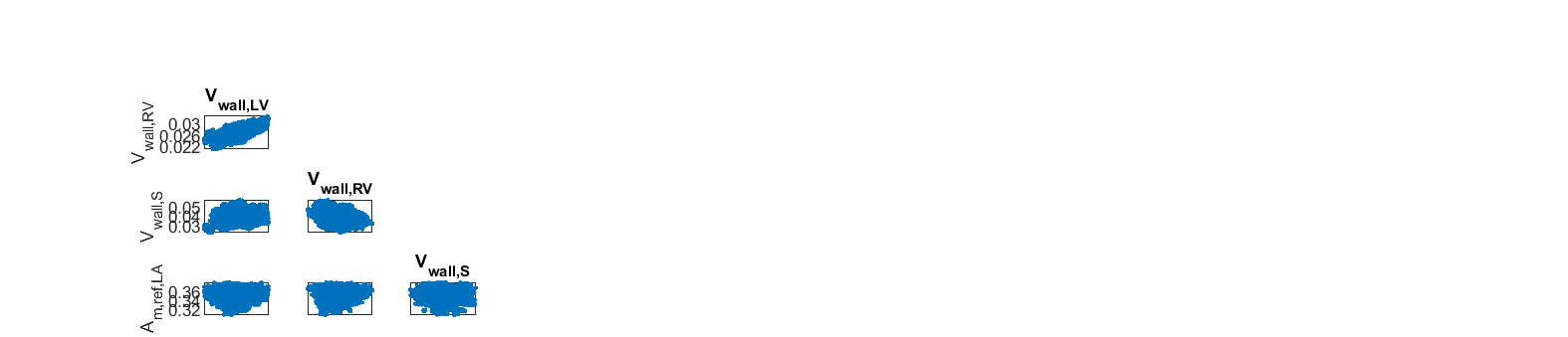

Supplement: S2 Text — (DOCX) [file pcbi.1010017.s002.docx]
